# Indirect identification of horizontal gene transfer

**DOI:** 10.1007/s00285-021-01631-0

**Published:** 2021-07-03

**Authors:** David Schaller, Manuel Lafond, Peter F. Stadler, Nicolas Wieseke, Marc Hellmuth

**Affiliations:** 1 Max Planck Institute for Mathematics in the Sciences, Inselstraße 22, 04109 Leipzig, Germany; 2 Bioinformatics Group, Department of Computer Science, Leipzig University, Härtelstraße 16-18, 04107 Leipzig, Germany; 3 Interdisciplinary Center of Bioinformatics, Leipzig University, Härtelstraße 16-18, 04107 Leipzig, Germany; 4 Department of Computer Science, Université de Sherbrooke, 2500 boul. de l’Université, Sherbrooke, QC J1K 2R1 Canada; 5 Swarm Intelligence and Complex Systems Group, Department of Computer Science, Leipzig University, Augustusplatz 10, 04109 Leipzig, Germany; 6 German Centre for Integrative Biodiversity Research (iDiv) Halle-Jena-Leipzig, Leipzig University, Härtelstraße 16-18, 04107 Leipzig, Germany; 7 Competence Center for Scalable Data Services and Solutions, Leipzig University, Härtelstraße 16-18, 04107 Leipzig, Germany; 8 Leipzig Research Center for Civilization Diseases, Leipzig University, Härtelstraße 16-18, 04107 Leipzig, Germany; 9 Max-Planck-Institute for Mathematics in the Sciences, Inselstraße 22, 04103 Leipzig, Germany; 10 Inst. f. Theoretical Chemistry, University of Vienna, Währingerstraße 17, 1090 Wien, Austria; 11 Facultad de Ciencias, Universidad National de Colombia, Sede Bogotá, Colombia; 12 Santa Fe Institute, 1399 Hyde Park Rd., Santa Fe, NM 87501 USA; 13 Department of Mathematics, Faculty of Science, Stockholm University, 106 91 Stockholm, Sweden

**Keywords:** Gene families, Xenology, Binary relation, Indirect phylogenetic methods, Horizontal gene transfer, Fitch graph, Later-divergence-time, Polynomial-time recognition algorithm, 92-08, 92D15, 68R01

## Abstract

Several implicit methods to infer horizontal gene transfer (HGT) focus on pairs of genes that have diverged only after the divergence of the two species in which the genes reside. This situation defines the edge set of a graph, the later-divergence-time (LDT) graph, whose vertices correspond to genes colored by their species. We investigate these graphs in the setting of relaxed scenarios, i.e., evolutionary scenarios that encompass all commonly used variants of duplication-transfer-loss scenarios in the literature. We characterize LDT graphs as a subclass of properly vertex-colored cographs, and provide a polynomial-time recognition algorithm as well as an algorithm to construct a relaxed scenario that explains a given LDT. An edge in an LDT graph implies that the two corresponding genes are separated by at least one HGT event. The converse is not true, however. We show that the complete xenology relation is described by an rs-Fitch graph, i.e., a complete multipartite graph satisfying constraints on the vertex coloring. This class of vertex-colored graphs is also recognizable in polynomial time. We finally address the question “how much information about all HGT events is contained in LDT graphs” with the help of simulations of evolutionary scenarios with a wide range of duplication, loss, and HGT events. In particular, we show that a simple greedy graph editing scheme can be used to efficiently detect HGT events that are implicitly contained in LDT graphs.

## Introduction

Horizontal gene transfer (HGT) laterally introduces foreign genetic material into a genome. The phenomenon is particularly frequent in prokaryotes (Soucy et al. 2015; Nelson-Sathi et al. 2015) but also contributed to shaping eukaryotic genomes (Keeling and Palmer 2008; Husnik and McCutcheon 2018; Acuña et al. 2012; Li et al. 2014; Moran and Jarvik 2010; Schönknecht et al. 2013). HGT may be additive, in which case its effect is similar to gene duplications, or lead to the replacement of a vertically inherited homolog. From a phylogenetic perspective, HGT leads to an incongruence of gene trees and species trees, thus complicating the analysis of gene family histories.

A broad spectrum of computational methods have been developed to identify horizontally transferred genes and/or HGT events, recently reviewed by Ravenhall et al. (2015). Parametric methods use genomic signatures, i.e., sequence features specific to a (group of) species identify horizontally inserted material. Genomic signatures include e.g. GC content, *k*-mer distributions, sequence autocorrelation, or DNA deformability (Dufraigne et al. 2005; Becq et al. 2010). Direct (or “explicit”) phylogenetic methods start from a given gene tree *T* and species tree *S* and compute a reconciliation, i.e., a mapping of the gene tree into the species tree. This problem first arose in the context of host/parasite assemblages (Page 1994; Charleston 1998) considering the equivalent problem of mapping a parasite tree *T* to a host phylogeny *S* such that the number of events such as host-switches, i.e., horizontal transfers, is minimized. For a review of the early literature we refer to Charleston and Perkins (2006). A major difficulty is to enforce time consistency in the presence of multiple horizontal transfer events, which renders the problem of finding optimal reconciliations NP-hard (Hallett and Lagergren 2001; Ovadia et al. 2011; Tofigh et al. 2011; Hasić and Tannier 2019). Nevertheless several practical approaches have become available, see e.g. Tofigh et al. (2011), Chen et al. (2012) and Ma et al. (2018).

Indirect (or “implicit”) phylogenetic methods forego the reconstruction of trees and start from sequence similarity or evolutionary distances and use unexpectedly small or large distances between genes as indicators of HGT. While indirect methods have been used successfully in the past, reviewed by Ravenhall et al. (2015), they have received very little attention from a more formal point of view. In this contribution, we focus on a particular type of implicit phylogenetic information, following the ideas of Novichkov et al. (2004). The basic idea is that the evolutionary distance between orthologous genes is approximately proportional to the distances between their species. Xenologous gene pairs as well as duplicate genes thus appear as outliers (Lawrence and Hartl 1992; Clarke et al. 2002; Novichkov et al. 2004; Dessimoz et al. 2008). More precisely, consider a family of homologous genes in a set of species and plot the phylogenetic distance of pairs of most similar homologs as a function of the phylogenetic distances between the species in which they reside. Since distances between orthologous genes can be expected to be approximately proportional to the distances between the species, orthologous pairs fall onto a regression line that defines equal divergence time for the last common ancestor of corresponding gene and species pairs. The gene pairs with “later divergence times”, i.e., those that are more closely related than expected from their species, fall below the regression line (Novichkov et al. 2004). Kanhere and Vingron (2009) complemented this idea with a statistical test based on the Cook distance to identify xenologous pairs in a statistically sound manner. For the mathematical analysis we assume that we can perfectly identify all pairs of genes *a* and *b* that are more closely related than expected from the phylogenetic distance of their respective genomes. Naturally, this defines a graph $(G,\sigma )$, whose vertices *x* (the genes) are colored by the species $\sigma (x)$ in which they appear. Here, we are interested in two questions:

1. What are the mathematical properties that characterize these “*later-divergence-time*” (*LDT*) graphs?
2. What kind of information about HGT events, the gene and species tree, and the reconciliation map between them is contained implicitly in an LDT graph?

In Sect. 6 we will briefly consider the situation that later-divergence-time information is fraught with experimental errors.

These questions are motivated by a series of recent publications that characterized the mathematical structure of orthology (Hellmuth et al. 2013; Lafond and El-Mabrouk 2014), the xenology relation *sensu* Fitch (Geiß et al. 2018; Hellmuth et al. 2018; Hellmuth and Seemann 2019), and the (reciprocal) best match relation (Geiß et al. 2019, 2020b; Schaller et al. 2021a, b). Each of these relations satisfies stringent mathematical conditions that—at least in principle—can be used to correct empirical estimates and thus serve as a potential means of noise reduction (Hellmuth et al. 2015; Stadler et al. 2020). This approach has also lead to efficient algorithms to extract gene trees, species trees, and reconciliations from the relation data. Although the resulting representations of gene family histories are usually not fully resolved, they can provide important constraints for subsequent refinements. The advantage of the relation-based approach is primarily robustness. While the inference of phylogenetic trees relies on detailed probability models or the additivity of distance metrics, our approach starts from yes/no answers to simple, pairwise comparisons. These data can therefore be represented as edges in a graph, possibly augmented by a measure of confidence. Noise and inaccuracies in the initial estimates then translate into violations of the required mathematical properties of the graphs in question. Graph editing approaches can therefore be harnessed as a means of noise reduction (Hellmuth et al. 2015; Dondi et al. 2017; Lafond and El-Mabrouk 2014; Lafond et al. 2016; Hellmuth et al. 2020b, a; Schaller et al. 2021c).

Previous work following this paradigm has largely been confined to duplication-loss (DL) scenarios, excluding horizontal transfer. As shown in Hellmuth (2017), it is possible to partition a gene set into HGT-free classes separated by HGTs. Within each class, the reconstruction problems then simplify to the much easier DL scenarios. It is of utmost interest, therefore, to find robust methods to infer this partition directly from (dis)similarity data. Here, we explore the usefulness and limitations of LDT graphs for this purpose.

This contribution is organized as follows. After introducing the necessary notation, we introduce *relaxed scenarios*, a very general framework to describe evolutionary scenarios that emphasizes time consistency of reconciliation rather than particular types of evolutionary events. In Sect. 4, LDT graphs are defined formally and characterized as those properly colored cographs for which a set of accompanying rooted triples is consistent (Theorem 3). The proof is constructive and provides a method (Algorithm 1) to compute a relaxed scenario for a given LDT graph. Section 5 defines HGT events, shows that every edge in a LDT graph corresponds to an HGT event, and characterizes those LDT graphs that already capture all HGT events. In addition, we provide a characterization of “rs-Fitch graphs” (general vertex-colored graphs that capture all HGT events) in terms of their coloring. These properties can be verified in polynomial time. Since LDT graphs do not usually capture all HGT events, we discuss in “Appendix C” several ways to obtain a plausible set of HGT candidates from LDT graphs. In Sect. 7, we address the question “how much information about all HGT events is contained in LDT graphs” with the help of simulations of evolutionary scenarios with a wide range of duplication, loss, and HGT events. We find that LDT graphs cover roughly a third of xenologous pairs, while a simple greedy graph editing scheme can more than double the recall at moderate false positive rates. This greedy approach already yields a median accuracy of $89 \%$, and in $99.8 \%$ of the cases produces biologically feasible solutions in the sense that the inferred graphs are rs-Fitch graphs. We close with a discussion of several open problems and directions for future research in Sect. 8.

The material of this contribution is extensive and contains several lengthy, very technical proofs. We therefore divided the presentation into a Narrative Part that contains only those mathematical results that contribute to our main conclusions, and a Technical Part providing additional results and all proofs. To facilitate cross-referencing between the two parts, the same numbering of Definitions, Lemmas, Theorems, etc., is used. Appendices A, B, and C contain the technical material corresponding to Sects. 4, 5, and 6, respectively.

## Notation

*Graphs* We consider undirected graphs $G=(V,E)$ with vertex set $V(G):=V$ and edge set $E(G):=E$, and denote edges connecting vertices $x,y\in V$ by *xy*. The graphs $K_1$ and $K_2$ denote the complete graphs on one and two vertices, respectively. The graph $K_2+K_1$ is the disjoint union of a $K_2$ and a $K_1$.

The join $G\triangledown H$ of two graphs $G=(V,E)$ and $H=(W,F)$ is the graph with vertex set  and edge set . We write $H\subseteq G$ if $V(H)\subseteq V(G)$ and $E(H)\subseteq E(G)$, in which case *H* is called a *subgraph of* *G*. Given a graph $G=(V,E)$, we write *G*[*W*] for the graph induced by $W\subseteq V$. A *connected component* *C* of *G* is an inclusion-maximal vertex set such that *G*[*C*] is connected. A *(maximal) clique* *C* in an undirected graph *G* is an (inclusion-maximal) vertex set such that, for all vertices $x,y\in C$, it holds that $xy\in E(G)$, i.e., *G*[*C*] is *complete*. A subset $W\subseteq V$ is a *(maximal) independent set* if *G*[*W*] is edgeless (and *W* is maximal w.r.t. inclusion). A graph $G = (V,E)$ is *complete multipartite* if *V* consists of $k\ge 1$ pairwise disjoint independent sets $I_1,\dots , I_k$ and $xy\in E$ if and only if $x\in I_i$ and $y\in I_j$ with $i\ne j$.

A graph *G* together with a vertex coloring $\sigma$, denoted by $(G,\sigma )$, is *properly colored* if $uv \in E(G)$ implies $\sigma (u)\ne \sigma (v)$. For a coloring $\sigma :V\rightarrow M$ and a subset $W\subseteq V$, we write $\sigma (W) :=\{\sigma (w)\mid w\in W\}$ for the set of colors that appear on the vertices in *W*. Throughout, we will need restrictions of the coloring map $\sigma$.

### Definition 1

Let $\sigma :L\rightarrow M$ be a map, $L'\subseteq L$ and $\sigma (L') \subseteq M' \subseteq M$. Then, the map $\sigma _{|L',M'}:L'\rightarrow M'$ is defined by putting $\sigma _{|L',M'}(v) = \sigma (v)$ for all $v\in L'$. If we only restrict the domain of $\sigma$, we just write $\sigma _{|L'}$ instead of $\sigma _{|L',M}$.

We do neither assume that $\sigma$ nor that its restriction $\sigma _{|L',M'}$ is surjective.

*Rooted trees* All trees appearing in this contribution are rooted in one of their vertices. We write $x \preceq _{T} y$ if *y* lies on the unique path from the root to *x*, in which case *y* is called an ancestor of *x*, and *x* is called a descendant of *y*. We may also write $y \succeq _{T} x$ instead of $x \preceq _{T} y$. We use $x \prec _T y$ for $x \preceq _{T} y$ and $x \ne y$. In the latter case, *y* is a *strict ancestor* of *x*. If $x \preceq _{T} y$ or $y \preceq _{T} x$, the vertices *x* and *y* are *comparable* and, otherwise, *incomparable*. We write *L*(*T*) for the set of leaves of the tree *T*, i.e., the $\preceq _T$-minimal vertices and say that *T* is a tree *on L(T)*. We write *T*(*u*) for the subtree of *T* rooted in *u*. The *last common ancestor* of a vertex set $W\subseteq V(T)$ is the $\preceq _T$-minimal vertex $u:={{\,\mathrm{lca}\,}}_T(W)$ for which $w\preceq _T u$ for all $w\in W$. For brevity we write ${{\,\mathrm{lca}\,}}_T(x,y)={{\,\mathrm{lca}\,}}_T(\{x,y\})$.

We employ the convention that edges (*x*, *y*) in a tree are always written such that $y \preceq _{T} x$ is satisfied. If (*x*, *y*) is an edge in *T*, then ${{\,\mathrm{par}\,}}(y):=x$ is the *parent* of *y*, and *y* the *child* of *x*. We denote with ${{\,\mathrm{child}\,}}_T(x)$ the set of all children of *x* in *T*. It will be convenient for the discussion below to extend the ancestor relation $\preceq _T$ on *V* to the union of the edge and vertex sets of *T*. More precisely, for a vertex $x\in V(T)$ and an edge $e=(u,v)\in E(T)$ we put $x \prec _T e$ if and only if $x\preceq _T v$; and $e \prec _T x$ if and only if $u\preceq _T x$. In addition, for edges $e=(u,v)$ and $f=(a,b)$ in *T* we put $e\preceq _T f$ if and only if $v \preceq _T b$.

A rooted tree is *phylogenetic* if all vertices that are adjacent to at least two vertices have at least two children. A rooted tree *T* is planted if its root has degree 1. In this case, we denote the “planted root” by $0_T$. In planted phylogenetic trees there is a unique “planted edge” $(0_T,\rho _T)$ where $\rho _T:={{\,\mathrm{lca}\,}}_T(L(T))$. Note that by definition $0_T\notin L(T)$.

*Throughout, we will assume that all trees are rooted and phylogenetic unless explicitly stated otherwise. Whenever there is no danger of confusion, we will refer also to planted phylogenetic trees simply as trees.*

The set of *inner vertices* is given by $V^0(T):=V(T){\setminus } (L(T)\cup \{0_T\})$. An edge (*u*, *v*) is an *inner* edge if both vertices *u* and *v* are inner vertices and, otherwise, an *outer* edge. The restriction of *T* to a subset $L'\subseteq L(T)$ of leaves, denoted by $T_{|L'}$ is obtained by identifying the (unique) minimal subtree of *T* that connects all leaves in $L'$, and suppressing all vertices with degree two except possibly the root $\rho _{T_{L'}}={{\,\mathrm{lca}\,}}_T(L')$. *T* *displays* a tree $T'$, in symbols $T'\le T$, if $T'$ can be obtained from a restriction $T_{|L'}$ of *T* by a series of inner edge contractions (Bryant and Steel 1995). If, in addition, $L(T)=L(T')$, then *T* is a *refinement* of $T'$. Throughout this contribution, we will consider leaf-colored trees $(T,\sigma )$ with $\sigma$ being defined for *L*(*T*) only.

*Rooted triples* A rooted triple is a tree *T* on three leaves and two internal vertices. We write *ab*|*c* for the triple with ${{\,\mathrm{lca}\,}}_T(a,b)\prec {{\,\mathrm{lca}\,}}_T(a,c)={{\,\mathrm{lca}\,}}_T(b,c)$. For a set ${\mathcal {R}}$ of triples we write $L({\mathcal {R}}):=\bigcup _{{\mathsf {t}}\in {\mathcal {R}}}L({\mathsf {t}})$. The set ${\mathcal {R}}$ is *compatible* if there is a tree *T* with $L({\mathcal {R}}) \subseteq L(T)$ that displays every triple ${\mathsf {t}}\in {\mathcal {R}}$. The construction of such a tree *T* from a triple set ${\mathcal {R}}$ on *L* makes use of an auxiliary graph that will play a prominent role in this contribution.

### Definition 2

(Aho et al. 1981) Let ${\mathcal {R}}$ be a set of rooted triples on the vertex set *L*. The *Aho graph* $[{\mathcal {R}},L]$ has vertex set *L* and edge set $\{ xy \mid \exists z\in L:\, xy|z \in {\mathcal {R}}\}$.

The algorithm BUILD (Aho et al. 1981) uses Aho graphs in a top-down recursion starting from a given set of triples ${\mathcal {R}}$ and returns for compatible triple sets ${\mathcal {R}}$ on *L* an unambiguously defined tree ${{\,\mathrm{Aho}\,}}({\mathcal {R}}, L)$ on *L*, which is known as the *Aho tree*. BUILD runs in polynomial time. The key property of the Aho graph that ensures the correctness of BUILD can be stated as follows:

### Proposition 1

(Aho et al. 1981; Bryant and Steel 1995) A set of triples ${\mathcal {R}}$ is compatible if and only if for each subset $L\subseteq L({\mathcal {R}})$ with $|L|>1$ the graph $[{\mathcal {R}},L]$ is disconnected.

*Cographs* are recursively defined as undirected graphs that can be generated as joins or disjoint unions of cographs, starting from single-vertex graphs $K_1$. The recursive construction defines a rooted tree (*T*, *t*), called *cotree*, whose leaves are the vertices of the cograph *G*, i.e., the $K_1$s, while each of its inner vertices *u* of *T* represent the join or disjoint union operations, labeled as $t(u)=1$ and $t(u)=0$, respectively. Hence, for a given cograph *G* and its cotree (*T*, *t*), we have $xy\in E(G)$ if and only if $t({{\,\mathrm{lca}\,}}_T(x,y))=1$. Contraction of all tree edges $(u,v)\in E(T)$ with $t(u)=t(v)$ results in the *discriminating cotree* $(T_G,{{\hat{t}}})$ of *G* with cotree-labeling ${{\hat{t}}}$ such that ${{\hat{t}}}(u)\ne {{\hat{t}}}(v)$ for any two adjacent interior vertices of $T_G$. The discriminating cotree $(T_G,{{\hat{t}}})$ is uniquely determined by *G* (Corneil et al. 1981a). Cographs have a large number of equivalent characterizations. In this contribution, we will need the following classical results:

### Proposition 2

(Corneil et al. 1981a) Given an undirected graph *G*, the following statements are equivalent:

1. *G* is a cograph.
2. *G* does not contain a $P_4$, i.e., a path on four vertices, as an induced subgraph.
3. $\mathrm {diam}(H) \le 2$ for all connected induced subgraphs *H* of *G*.
4. Every induced subgraph *H* of *G* is a cograph.

## Relaxed reconciliation maps and relaxed scenarios

Tofigh et al. (2011) and Bansal et al. (2012) define “Duplication-Transfer-Loss” (DTL) scenarios in terms of a vertex-only map $\gamma :V(T)\rightarrow V(S)$. The H-trees introduced by Górecki (2010) and Górecki and Tiuryn (2012) formalize the same concept in a very different manner. A definition of a DTL-like class of scenarios in terms of a reconciliation map $\mu : V(T)\rightarrow V(S)\cup E(S)$ was analyzed by Nøjgaard et al. (2018). For binary trees, the two definitions are equivalent; for non-binary trees, however, the DTL-scenarios are a proper subset, see Nøjgaard et al. (2018, Fig. 1) for an example. Several other mathematical frameworks have been used in the literature to specify evolutionary scenarios. Examples include the DLS-trees of Górecki and Tiuryn (2006), which can be seen as event-labeled gene trees with leaves denoting both surviving genes and loss-events, maps $g:V(S')\rightarrow 2^{V(T)}$ from a suitable subdivision $S'$ of the species tree *S* to the gene tree as used by Hallett and Lagergren (2001), and associations of edges, i.e., subsets of $E(T)\times E(S)$ (Wieseke et al. 2013).

In the presence of HGT, the relationships of gene trees and species are not only constrained by local conditions corresponding to the admissible local evolutionary events (duplication, speciation, gene loss, and HGT) but also by the global condition that the HGT events within each lineage admit a temporal order (Merkle and Middendorf 2005; Gorbunov and Lyubetsky 2009; Tofigh et al. 2011). In order to capture time consistency from the outset and to establish the mathematical framework, we consider here trees with explicit timing information (Merkle and Middendorf 2005).

### Definition 3

(*Time Map*) The map $\tau _{T}: V(T) \rightarrow {\mathbb {R}}$ is a time map for a tree *T* if $x\prec _T y$ implies $\tau _{T}(x)<\tau _{T}(y)$ for all $x,y\in V(T)$.

It is important to note that only *qualitative*, relative timing information will be used in practice, i.e., we will never need the actual value of time maps but only information on whether an event pre-dates, post-dates, or is concurrent with another. Definition 3 ensures that the ancestor relation $\preceq _T$ and the timing of the vertices are not in conflict. For later reference, we provide the following simple result.

### Lemma 1

Given a tree *T*, a time map $\tau _{T}$ for *T* satisfying $\tau _{T}(x)=\tau _0(x)$ with arbitrary choices of $\tau _0(x)$ for all $x\in L(T)$ can be constructed in linear time.

### Proof

We traverse *T* in postorder. If *x* is a leaf, we set $\tau _{T}(x)=\tau _0(x)$, and otherwise compute $t:=\max _{u\in {{\,\mathrm{child}\,}}(x)} \tau _{T}(u)$ and set $\tau _{T}(x)=t'$ with an arbitrary value $t'> t$. Clearly the total effort is $O(|V(T)|+|E(T)|)$, and thus also linear in the number of leaves *L*(*T*). $\square$

Lemma 1 will be useful for the construction of time maps as it, in particular, allows us to put $\tau _{T}(x)=\tau _{T}(y)$ for all $x,y\in L(T)$.

### Definition 4

(*Time consistency*) Let *T* and *S* be two trees. A map $\mu :V(T) \rightarrow V(S) \cup E(S)$ is called *time-consistent* if there are time maps $\tau _{T}$ for *T* and $\tau _{S}$ for *S* satisfying the following conditions for all $u \in V(T)$:

1. If $$\mu (u) \in V(S)$$, then $$\tau _{T}(u) = \tau _{S}(\mu (u))$$.
2. Else, if $\mu (u) = (x,y) \in E(S)$, then $\tau _{S}(y)<\tau _{T}(u)<\tau _{S}(x)$.

Conditions (C1) and (C2) ensure that the reconciliation map $\mu$ preserves time in the following sense: If vertex *u* of the gene tree is mapped to a vertex $\mu (u)=v$ in the species tree, then *u* and *v* receive the same time stamp by Condition (C1). If *u* is mapped to an edge $\mu (u) = (x,y)$, then the time stamp of *u* falls within the time range $[\tau _{S}(x),\tau _{S}(y)]$ of the edge *xy* in the species tree. The following definition of reconciliation is designed (1) to be general enough to encompass the notions of reconciliation that have been studied in the literature, and (2) to separate the mapping between gene tree and species tree from specific types of events. Event types such as duplication or horizontal transfer therefore are considered here as a matter of *interpreting* scenarios, not as part of their definition.

### Definition 5

(*Relaxed reconciliation map*) Let *T* and *S* be two planted trees with leaf sets *L*(*T*) and *L*(*S*), respectively and let $\sigma :L(T)\rightarrow L(S)$ be a map. A map $\mu :V(T)\rightarrow V(S)\cup E(S)$ is a *relaxed reconciliation map* for $(T,S,\sigma )$ if the following conditions are satisfied:

(G0) *Root Constraint.* $\mu (x) = 0_{S}$ if and only if $x = 0_{T}$
(G1) *Leaf Constraint.* $\mu (x)=\sigma (x)$ if and only if $x\in L(T)$.
(G2) *Time Consistency Constraint.* The map $\mu$ is time-consistent for some time maps $\tau _{T}$ for *T* and $\tau _{S}$ for *S*.

Condition (G0) is used to map the respective planted roots. (G1) ensures that genes are mapped to the species in which they reside. (G2) enforces time consistency. The reconciliation maps most commonly used in the literature, see e.g. (Tofigh et al. 2011; Bansal et al. 2012), usually not only satisfy (G0)–(G2) but also impose additional conditions. We therefore call the map $\mu$ defined here “relaxed”.

### Definition 6

(*relaxed Scenario*) The 6-tuple ${\mathcal {S}}= (T,S,\sigma ,\mu ,\tau _{T},\tau _{S})$ is a *relaxed scenario* if $\mu$ is a relaxed reconciliation map for $(T,S,\sigma )$ that satisfies (G2) w.r.t. the time maps $\tau _{T}$ and $\tau _{S}$.

By definition, relaxed reconciliation maps are time-consistent. Moreover, $\tau _{T}(x)=\tau _{S}(\sigma (x))$ for all $x \in L(T)$ by Definitions 4(C1) and 5(G1,G2). In the following we will refer to the map $\sigma :L(T)\rightarrow L(S)$ as the *coloring of* ${\mathcal {S}}$.

## Later-divergence-time graphs

### LDT graphs and $\mu$-free scenarios

In the absence of horizontal gene transfer, the last common ancestor of two species *A* and *B* should mark the latest possible time point at which two genes *a* and *b* residing in $\sigma (a)=A$ and $\sigma (b)=B$, respectively, may have diverged. Situations in which this constraint is violated are therefore indicative of HGT. To address this issue in some more detail, we next define “$\mu$-free scenarios” that eventually will lead us to the class of “LDT graphs” that contain all information about genes that diverged after the species in which they reside.

#### Definition 7

(${\mu }$*-free scenario*) Let *T* and *S* be planted trees, $\sigma :L(T)\rightarrow L(S)$ be a map, and $\tau _{T}$ and $\tau _{S}$ be time maps of *T* and *S*, respectively, such that $\tau _{T}(x) = \tau _{S}(\sigma (x))$ for all $x\in L(T)$. Then, ${\mathcal {T}}=(T,S,\sigma ,\tau _{T},\tau _{S})$ is called a $\mu$-*free scenario*.

This definition of a scenario without a reconciliation map $\mu$ is mainly a technical convenience that simplifies the arguments in various proofs by avoiding the construction of a reconciliation map. It is motivated by the observation that the “later-divergence-time” of two genes in comparison with their species is independent from any such $\mu$. Every relaxed scenario ${\mathcal {S}}=(T,S,\sigma ,\mu ,\tau _{T},\tau _{S})$ implies an underlying $\mu$-free scenario ${\mathcal {T}}=(T,S,\sigma ,\tau _{T},\tau _{S})$. Statements proved for $\mu$-free scenarios therefore also hold for relaxed scenarios. Note that, by Lemma 1, given the time map $\tau _{S}$, one can easily construct a time map $\tau _{T}$ such that $\tau _{T}(x) = \tau _{S}(\sigma (x))$ for all $x\in L(T)$. In particular, when constructing relaxed scenarios explicitly, we may simply choose $\tau _{T}(u)=0$ and $\tau _{S}(x)=0$ as common time for all leaves $u\in L(T)$ and $x\in L(S)$. Although not all $\mu$-free scenarios admit a reconciliation map and thus can be turned into relaxed scenarios, Lemma 2 below implies that for every $\mu$-free scenario ${\mathcal {T}}$ there is a relaxed scenario with possibly slightly distorted time maps that encodes the same LDT graph as ${\mathcal {T}}$.

#### Definition 8

(*LDT graph*) For a $\mu$-free scenario ${\mathcal {T}}=(T,S,\sigma ,\tau _{T},\tau _{S})$, we define $G_{_{<}}({\mathcal {T}}) = G_{_{<}}(T,S,\sigma ,\tau _{T},\tau _{S}) = (V,E)$ as the graph with vertex set $V:=L(T)$ and edge set

$$\begin{aligned} E :=\{ab\mid a,b\in L(T), \tau _{T}({{\,\mathrm{lca}\,}}_T(a,b))<\tau _{S}({{\,\mathrm{lca}\,}}_S(\sigma (a),\sigma (b))). \} \end{aligned}$$

A vertex-colored graph $(G,\sigma )$ is a *later-divergence-time graph (LDT graph)*, if there is a $\mu$-free scenario ${\mathcal {T}}=(T,S,\sigma ,\tau _{T},\tau _{S})$ such that $G=G_{_{<}}({\mathcal {T}})$. In this case, we say that ${\mathcal {T}}$ *explains* $(G,\sigma )$.

It is easy to see that the edge set of $G_{_{<}}({\mathcal {T}})$ defines an *undirected* graph and that two genes *a* and *b* form an edge if the divergence time of *a* and *b* is strictly less than the divergence time of the underlying species $\sigma (a)$ and $\sigma (b)$. Moreover, there are no edges of the form *aa*, since $\tau _{T}({{\,\mathrm{lca}\,}}_T(a,a)) = \tau _{T}(a) = \tau _{S}(\sigma (a)) =\tau _{S}({{\,\mathrm{lca}\,}}_S(\sigma (a),\sigma (a)))$. Hence $G_{_{<}}({\mathcal {T}})$ is a simple graph.

By definition, every relaxed scenario ${\mathcal {S}}=(T,S,\sigma ,\mu ,\tau _{T},\tau _{S})$ satisfies $\tau _{T}(x)=\tau _{S}(\sigma (x))$ all $x \in L(T)$. Therefore, removing $\mu$ from ${\mathcal {S}}$ yields a $\mu$-free scenario ${\mathcal {T}}=(T,S,\sigma ,\tau _{T},\tau _{S})$. Thus, we will use the following simplified notation.

#### Definition 9

We put $G_{_{<}}({\mathcal {S}}) :=G_{_{<}}(T,S,\sigma ,\tau _{T},\tau _{S})$ for a given relaxed scenario ${\mathcal {S}}=(T,S,\sigma ,\mu ,\tau _{T},\tau _{S})$ and the underlying $\mu$-free scenario $(T,S,\sigma ,\tau _{T},\tau _{S})$ and say, by slight abuse of notation, that ${\mathcal {S}}$ *explains* $(G_{_{<}}({\mathcal {S}}),\sigma )$.

The next two results show that the existence of a reconciliation map $\mu$ does not impose additional constraints on LDT graphs.

#### Lemma 2

For every $\mu$-free scenario ${\mathcal {T}}=(T,S,\sigma ,\tau _{T},\tau _{S})$, there is a relaxed scenario ${\mathcal {S}}=(T,S,\sigma ,\mu ,{\widetilde{\tau _{T}}},{\widetilde{\tau _{S}}})$ for *T*, *S* and $\sigma$ such that $(G_{_{<}}({\mathcal {T}}),\sigma ) = (G_{_{<}}({\mathcal {S}}), \sigma )$.

#### Theorem 1

$(G,\sigma )$ is an LDT graph if and only if there is a relaxed scenario ${\mathcal {S}}= (T,S,\sigma ,\mu ,\tau _{T},\tau _{S})$ such that $(G,\sigma ) = (G_{_{<}}({\mathcal {S}}),\sigma )$.

#### Remark 1

From here on, we omit the explicit reference to Lemma 2 and Theorem 1 and assume that the reader is aware of the fact that every LDT graph is explained by some relaxed scenario ${\mathcal {S}}$ and that for every $\mu$-free scenario ${\mathcal {T}}=(T,S,\sigma ,\tau _{T},\tau _{S})$, there is a relaxed scenario ${\mathcal {S}}$ for *T*, *S* and $\sigma$ such that $(G_{_{<}}({\mathcal {T}}),\sigma ) = (G_{_{<}}({\mathcal {S}}), \sigma )$.

**Fig. 1:**
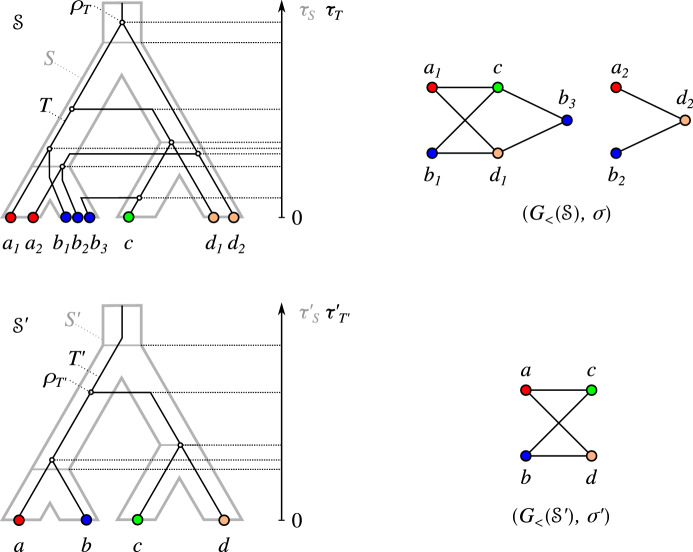
Top row: A relaxed scenario ${\mathcal {S}}=(T,S,\sigma ,\mu ,\tau _{T},\tau _{S})$ (left) with its LDT graph $(G_{_{<}}({\mathcal {S}}),\sigma )$ (right). The reconciliation map $\mu$ is shown implicitly by the embedding of the gene tree *T* into the species tree *S*. The times $\tau _{T}$ and $\tau _{S}$ are indicated by the position on the vertical axis, i.e., if a vertex *x* is drawn higher than a vertex *y*, this implies $\tau _{T}(y)<\tau _{T}(x)$. In subsequent figures we will not show the time maps explicitly. Bottom row: Another relaxed scenario ${\mathcal {S}}' =(T',S',\sigma ',\mu ',\tau _{T}',\tau _{S}')$ with a connected LDT graph $(G_{_{<}}({\mathcal {S}}'),\sigma ')$. As we shall see, connectedness of an LDT graph depends on the relative timing of the roots of the gene and species tree (cf. Lemma 11)

### Properties of LDT graphs

We continue by deriving several interesting characteristics LDT graphs.

#### Proposition 3

Every LDT graph $(G,\sigma )$ is properly colored.

As we shall see below, LDT graphs $(G,\sigma )$ contain detailed information about both the underlying gene trees *T* and species trees *S* for *all* $\mu$-scenarios that explain $(G,\sigma )$, and thus by Lemma 2 and Theorem 1 also about every relaxed scenario ${\mathcal {S}}$ satisfying $G=G_{_{<}}({\mathcal {S}})$. This information is encoded in the form of certain rooted triples that can be retrieved directly from local features in the colored graphs $(G,\sigma )$.

#### Definition 10

For a graph $G=(L,E)$, we define the set of triples on *L* as

$$\begin{aligned} {\mathfrak {T}}(G) :=\{xy|z \; :x,y,z\in L \text { are pairwise distinct, } xy\in E,\; xz,yz\notin E\} \,. \end{aligned}$$

If *G* is endowed with a coloring $\sigma :L\rightarrow M$ we also define a set of color triples

$$\begin{aligned} {\mathfrak {S}}(G,\sigma ) :=\{\sigma (x)\sigma (y)|\sigma (z)\; :&x,y,z\in L,\, \sigma (x),\sigma (y),\sigma (z) \text { are pairwise distinct},\\&xz, yz\in E,\; xy\notin E\}. \end{aligned}$$

#### Lemma 6

If a graph $(G,\sigma )$ is an LDT graph, then ${\mathfrak {S}}(G,\sigma )$ is compatible and *S* displays ${\mathfrak {S}}(G,\sigma )$ for every $\mu$-free scenario ${\mathcal {T}}=(T,S,\sigma ,\tau _{T},\tau _{S})$ that explains $(G,\sigma )$.

The next lemma shows that induced $K_2+K_1$ subgraphs in LDT graphs imply triples that must be displayed by the gene tree *T*.

#### Lemma 7

If $(G,\sigma )$ is an LDT graph, then ${\mathfrak {T}}(G)$ is compatible and *T* displays ${\mathfrak {T}}(G)$ for every $\mu$-free scenario ${\mathcal {T}}=(T,S,\sigma ,\tau _{T},\tau _{S})$ that explains $(G,\sigma )$.

The next results shows that LDT graphs cannot contain induced $P_4$s.

#### Lemma 8

Every LDT graph $(G,\sigma )$ is a properly colored cograph.

The converse of Lemma 8 is not true is in general. To see this, consider the properly-colored cograph $$(G,\sigma )$$ with vertex $$V(G)=\{a,a',b,b',c,c'\}$$, edges $$ab,bc, a'b',a'c' $$ and coloring $$\sigma (a)=\sigma (a')=A$$, $$\sigma (b)=\sigma (b')=B$$, and $$\sigma (c)=\sigma (c')=C$$ with *A*, *B*, *C* being pairwise distinct. In this case, $${\mathfrak {S}}(G,\sigma )$$ contains the triples *AC*|*B* and *BC*|*A*. By Lemma 6, the tree *S* in every $$\mu $$-free scenario $${\mathcal {T}}=(T,S,\sigma ,\tau _{T},\tau _{S})$$ or relaxed scenario $${\mathcal {S}}=(T,S,\sigma ,\mu ,\tau _{T},\tau _{S})$$ explaining $$(G,\sigma )$$ displays *AC*|*B* and *BC*|*A*. Since no such scenario can exist, $$(G,\sigma )$$ is not an LDT graph.

### Recognition and characterization of LDT graphs

In order to design an algorithm for the recognition of LDT graphs, we will consider partitions of the vertex set of a given input graph $(G=(L,E),\sigma )$. To construct suitable partitions, we start with the connected components of *G*. The coloring $\sigma :L\rightarrow M$ imposes additional constraints. We capture these with the help of binary relations that are defined in terms of partitions ${\mathscr {C}}$ of the color set *M* and employ them to further refine the partition of *G*.

#### Definition 12

Let $(G=(L,E),\sigma )$ be a graph with coloring $\sigma :L\rightarrow M$. Let ${\mathscr {C}}$ be a partition of *M*, and ${\mathscr {C}}'$ be the set of connected components of *G*. We define the following binary relation ${\mathfrak {R}}(G, \sigma , {\mathscr {C}})$ by setting

$$\begin{aligned} (x,y)\in {\mathfrak {R}}(G, \sigma , {\mathscr {C}}) \iff x,y\in L,\; \sigma (x), \sigma (y)&\in C \text { for some } C\in {\mathscr {C}}, \text { and } \\ x,y&\in C' \text { for some } C'\in {\mathscr {C}}'. \end{aligned}$$

By construction, two vertices $x,y\in L$ are in relation ${\mathfrak {R}}(G, \sigma , {\mathscr {C}})$ whenever they are in the same connected component of *G* and their colors $\sigma (x), \sigma (y)$ are contained in the same set of the partition of *M*. As shown in Lemma 9 in the Technical Part, the relation ${\mathfrak {R}}:={\mathfrak {R}}(G, \sigma , {\mathscr {C}})$ is an equivalence relation and every equivalence class of ${\mathfrak {R}}$ is contained in some connected component of *G*. In particular, each connected component of *G* is the disjoint union of ${\mathfrak {R}}$-classes.

The following partition of the leaf sets of subtrees of a tree *S* rooted at some vertex $u\in V(S)$ will be useful:

$$\begin{aligned}&\text {If } u \text { is not a leaf, then }&{\mathscr {C}}_{S}(u)&:=\{L(S(v)) \mid v\in {{\,\mathrm{child}\,}}_S(u)\} \\&\text {and, otherwise, }&{\mathscr {C}}_{S}(u)&:=\{\{u\}\}. \end{aligned}$$

One easily verifies that, in both cases, ${\mathscr {C}}_{S}(u)$ yields a valid partition of the leaf set *L*(*S*(*u*)). Recall that $\sigma _{|L',M'}:L'\rightarrow M'$ was defined as the “submap” of $\sigma$ with $L'\subseteq L$ and $\sigma (L') \subseteq M' \subseteq M$.

#### Lemma 10

Let $(G=(L,E),\sigma )$ be a properly colored cograph. Suppose that the triple set ${\mathfrak {S}}(G,\sigma )$ is compatible and let *S* be a tree on *M* that displays ${\mathfrak {S}}(G,\sigma )$. Moreover, let $L'\subseteq L$ and $u\in V(S)$ such that $\sigma (L') \subseteq L(S(u))$. Finally, set ${\mathfrak {R}}:={\mathfrak {R}}(G[L'],\sigma _{|L',L(S(u))},{\mathscr {C}}_{S}(u))$.

Then, for all distinct ${\mathfrak {R}}$-classes *K* and $K'$, either $xy\in E$ for all $x\in K$ and $y\in K'$, or $xy\notin E$ for all $x\in K$ and $y\in K'$. In particular, for $x\in K$ and $y\in K'$, it holds that

$$\begin{aligned} xy\in E \iff K, K' \text { are contained in the same connected component of } G[L']. \end{aligned}$$

**Fig. 2:**
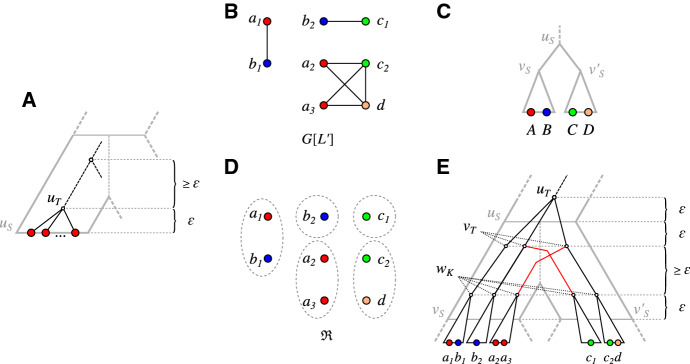
Visualization of Algorithm 1. **A** The case $u_S$ is a leaf (cf. Line 8). **B**–**E** The case $u_S$ is an inner vertex (cf. Line 12). **B** The subgraph of $(G,\sigma )$ induced by $L'$. **C** The local topology of the species tree *S* yields ${\mathscr {C}}_{S}(u_S)=\{\{A,B,\dots \},\{C,D,\dots \}\}$. Note that $L(S(u_S))$ may contain colors that are not present in $\sigma (L')$ (not shown). **D** The equivalence classes of ${\mathfrak {R}}:={\mathfrak {R}}(G[L'], \sigma _{|L',L(S(u))}, {\mathscr {C}}_{S}(u_S))$. **E** The vertex $u_T$ and the vertices $v_T$ are created in this recursion step. The vertices $w_K$ corresponding to the ${\mathfrak {R}}$-classes *K* are created in the next-deeper steps. Note that some vertices have only a single child, and thus get suppressed in Line 25

Lemma 10 suggests a recursive strategy to construct a relaxed scenario ${\mathcal {S}}=(T,S,\sigma ,\mu ,\tau _{T},\tau _{S})$ for a given properly-colored cograph $(G,\sigma )$, which is illustrated in Fig. 2. The starting point is a species tree *S* displaying all the triples in ${\mathfrak {S}}(G,\sigma )$ that are required by Lemma 6. We show below that there are no further constraints on *S* and thus we may choose $S={{\,\mathrm{Aho}\,}}({\mathfrak {S}}(G,\sigma ),L)$ and endow it with an arbitrary time map $\tau _{S}$. Given $(S,\tau _{S})$, we construct $(T,\tau _{T})$ in top-down order. In order to reduce the complexity of the presentation and to make the algorithm more compact and readable, we will not distinguish the cases in which $(G,\sigma )$ is connected or disconnected, nor whether a connected component is a superset of one or more ${\mathfrak {R}}$-classes. The tree *T* therefore will not be phylogenetic in general. We shall see, however, that this issue can be alleviated by simply suppressing all inner vertices with a single child.

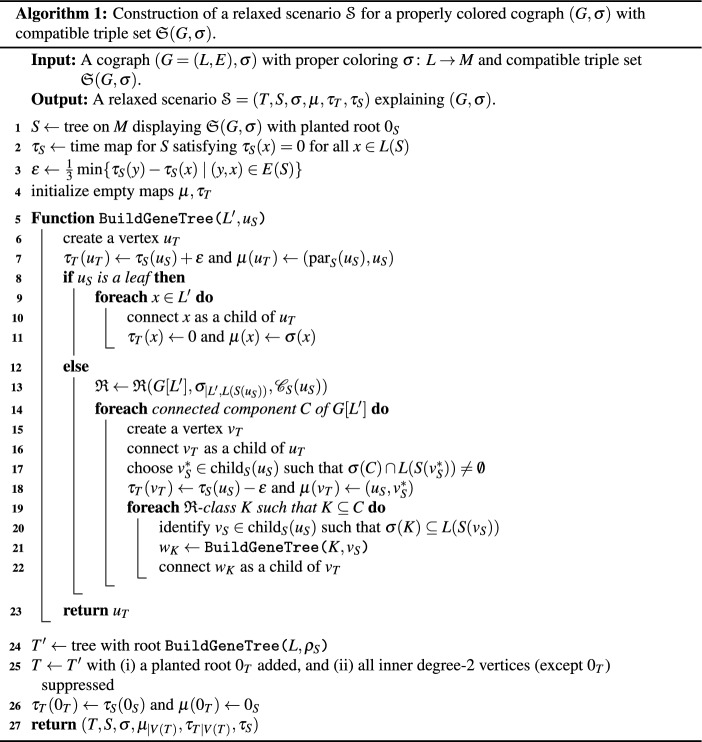



The root $u_T$ is placed above $\rho _S$ to ensure that no two vertices from distinct connected components of *G* will be connected by an edge in $G_{_{<}}({\mathcal {S}})$. The vertices $v_T$ representing the connected components *C* of *G* are each placed within an edge of *S* below $\rho _S$. W.l.o.g., the edges $(\rho _S,v_S)$ are chosen such that the colors of the corresponding connected component *C* and the colors in $L(S(v_S))$ overlap. Next we compute the relation ${\mathfrak {R}}:={\mathfrak {R}}(G,\sigma ,{\mathscr {C}}_{S}(\rho _S))$ and determine, for each connected component *C*, the ${\mathfrak {R}}$-classes *K* that are a subset of *C*. For each of them, a child $w_K$ is appended to the tree vertex $v_T$. The subtree $T(w_K)$ will have leaf set $L(T(w_K))=K$. Since ${\mathfrak {R}}$ is defined on ${\mathscr {C}}_{S}(\rho _S)$ in this first step, $G({\mathcal {S}})$ will have all edges between vertices that are in the same connected component *C* but in distinct ${\mathfrak {R}}$-classes (cf. Lemma 10). The definition of ${\mathfrak {R}}$ also implies that we always find a vertex $v_S\in {{\,\mathrm{child}\,}}_S(\rho _S)$ such that $\sigma (K)\subseteq L(S(v_S))$ (more detailed arguments for this are given in the proof of Claim 4 in the proof of Theorem 2 below). Thus we can place $w_K$ into this edge $(\rho _S,v_S)$, and proceed recursively on the ${\mathfrak {R}}$-classes $L':=K$, the induced subgraphs $G[L']$ and their corresponding vertices $v_S\in V(S)$, which then serve as the root of the species trees. More precisely, we identify $w_K$ with the root $u'_T$ created in the “next-deeper” recursion step. Since we alternate between vertices $u_T$ for which no edges between vertices of distinct subtrees exist, and vertices $v_T$ for which all such edges exist, we can label the vertices $u_T$ with “0” and the vertices $v_T$ with “1” and obtain a cotree for the cograph *G*.

This recursive procedure is described more formally in Algorithm 1 which also describes the constructions of an appropriate time map $\tau _{T}$ for *T* and a reconciliation map $\mu$. We note that we find it convenient to use as trivial case in the recursion the situation in which the current root $u_S$ of the species tree is a leaf rather than the condition $|L'|=1$. In this manner we avoid the distinction between the cases $u_S\in L(S)$ and $u_S\notin L(S)$ in the **else**-condition starting in Line 12. This results in a shorter presentation at the expense of more inner vertices that need to be suppressed at the end in order to obtain the final tree *T*. We proceed by proving the correctness of Algorithm 1.

#### Theorem 2

Let $(G,\sigma )$ be a properly colored cograph, and assume that the triple set ${\mathfrak {S}}(M,G)$ is compatible. Then Algorithm 1 returns a relaxed scenario ${\mathcal {S}}=(T,S,\sigma ,\mu ,\tau _{T},\tau _{S})$ such that $G_{_{<}}({\mathcal {S}})=G$ in polynomial time.

As a consequence of Lemma 6 and 8, and the fact that Algorithm 1 returns a relaxed scenario ${\mathcal {S}}$ for a given properly colored cograph with compatible triple set ${\mathfrak {S}}(G,\sigma )$, we obtain

#### Theorem 3

A graph $(G,\sigma )$ is an LDT graph if and only if it is a properly colored cograph and ${\mathfrak {S}}(G,\sigma )$ is compatible.

Theorem 3 has two consequences that are of immediate interest:

#### Corollary 2

LDT graphs can be recognized in polynomial time.

#### Corollary 3

The property of being an LDT graph is hereditary, that is, if $(G,\sigma )$ is an LDT graph then each of its vertex induced subgraphs is an LDT graph.

The relaxed scenarios ${\mathcal {S}}$ explaining an LDT graph $(G,\sigma )$ are far from being unique. In fact, we can choose from a large set of trees $(S,\tau _{S})$ that is determined only by the triple set ${\mathfrak {S}}(G,\sigma )$:

#### Corollary 4

If $(G=(L,E),\sigma )$ is an LDT graph with coloring $\sigma :L\rightarrow M$, then for all planted trees *S* on *M* that display ${\mathfrak {S}}(G,\sigma )$ there is a relaxed scenario ${\mathcal {S}}=(T,S,\sigma ,\mu ,\tau _{T},\tau _{S})$ that contains $\sigma$ and *S* and that explains $(G,\sigma )$.

As shown in the Technical Part, for every LDT graph $(G,\sigma )$ there is a relaxed scenario ${\mathcal {S}}=(T,S,\sigma ,\mu ,\tau _{T},\tau _{S})$ explaining $(G,\sigma )$ such that *T* displays the discriminating cotree $T_{G}$ of *G* (cf. Corollary 5 in the Technical Part). However, this property is not satisfied by all relaxed scenarios that explain an $(G,\sigma )$. Nevertheless, the latter results enable us to relate connectedness of LDT graphs to properties of the relaxed scenarios by which it can be explained (cf. Lemma 11 in Technical Part).

### Least resolved trees for LDT graphs

As we have seen e.g. in Corollary 4, there are in general many trees *S* and *T* forming relaxed scenarios ${\mathcal {S}}$ that explain a given LDT graph $(G,\sigma )$. This begs the question to what extent these trees are determined by “representatives”. For *S*, we have seen that *S* always displays ${\mathfrak {S}}(G,\sigma )$, suggesting to consider the role of $S={{\,\mathrm{Aho}\,}}({\mathfrak {S}}(G,\sigma ),M)$, where *M* is the codomain of $\sigma$. This tree is least resolved in the sense that there is no relaxed scenario explaining the LDT graph $(G,\sigma )$ with a tree $S'$ that is obtained from *S* by edge-contractions. The latter is due to the fact that any edge contraction in ${{\,\mathrm{Aho}\,}}({\mathfrak {S}}(G,\sigma ),M)$ yields a tree $S'$ that does not display ${\mathfrak {S}}(G,\sigma )$ any more (Jansson et al. 2012). By Proposition 6, none of the relaxed scenarios containing $S'$ explain the LDT graph $(G,\sigma )$.

#### Definition 13

Let ${\mathcal {S}}=(T,S,\sigma ,\mu ,\tau _{T},\tau _{S})$ be a relaxed scenario explaining the LDT graph $(G,\sigma )$. The planted tree *T* is *least resolved* for $(G,\sigma )$ if no relaxed scenario $(T',S',\sigma ',\mu ',\tau _{T}',\tau _{S}')$ with $T'<T$ explains $(G,\sigma )$.

In other words, *T* is least resolved for $(G,\sigma )$ if no relaxed scenario with a gene tree $T'$ obtained from *T* by a series of edge contractions explains $(G,\sigma )$.

**Fig. 3:**
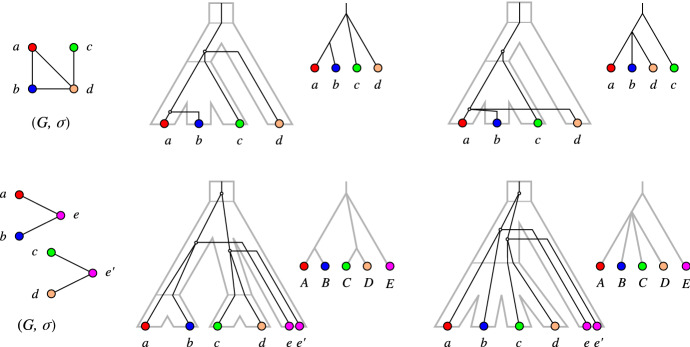
Examples of LDT graphs $(G,\sigma )$ with multiple least resolved trees. Top row: No unique least resolved gene tree. For both trees, contraction of the single inner edge leads to a loss of the gene triple $ab|c\in {\mathfrak {T}}(G)$ (cf. Lemma 7). The species tree is also least resolved since contraction of its single inner edge leads to loss of the species triples $\sigma (a)\sigma (c)|\sigma (d), \sigma (b)\sigma (c)|\sigma (d)\in {\mathfrak {S}}(G,\sigma )$ (cf. Lemma 6). Bottom row: No unique least resolved species tree. Both trees display the two necessary triples $AB|E,CD|E\in {\mathfrak {S}}(G,\sigma )$, and are again least resolved w.r.t. these triples. The gene trees are also least resolved since contraction of either of its two inner edges leads e.g. to loss of one of the triples $ae|c, ce'|a\in {\mathfrak {T}}(G)$

**Fig. 4:**
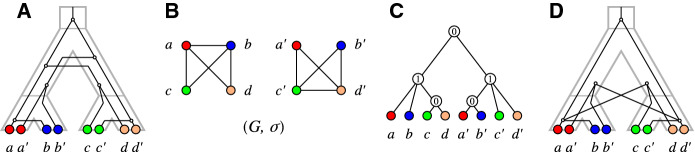
Example of an LDT graph $(G,\sigma )$ in **B** that is explained by the relaxed scenario shown in **A**. Here, $(G,\sigma )$ cannot be explained by a relaxed scenario ${\mathcal {S}}=(T,S,\sigma ,\mu , \tau _{T},\tau _{S})$ such that *T* is the unique discriminating cotree (shown in **C**) for the cograph *G*, see **D** and the text for further explanations

The examples in Fig. 3 show that LDT graphs are in general not accompanied by unique least resolved trees. In the top row, relaxed scenarios with different least resolved gene trees *T* and the same least resolved species tree *S* explain the LDT graph $(G,\sigma )$. In the example below, two distinct least resolved species trees exist for a given least-resolved gene tree.

The example in Fig. 4 shows, furthermore, that the unique discriminating cotree $T_G$ of an LDT graph $(G,\sigma )$ is not always “sufficiently resolved”. To see this, assume that the graph $(G,\sigma )$ in the example can be explained by a relaxed scenario ${\mathcal {S}}=(T,S,\sigma ,\mu , \tau _{T},\tau _{S})$ such that $T=T_G$. First consider the connected component consisting of *a*, *b*, *c*, *d*. Since ${{\,\mathrm{lca}\,}}_T(a,b)\succ _T {{\,\mathrm{lca}\,}}_T(c,d)$, $ab\in E(G)$ and $cd\notin E(G)$, we have $\tau _{S}({{\,\mathrm{lca}\,}}_S(\sigma (a),\sigma (b)))> \tau _{T}({{\,\mathrm{lca}\,}}_T(a,b))> \tau _{T}({{\,\mathrm{lca}\,}}_T(c,d))\ge \tau _{S}({{\,\mathrm{lca}\,}}_S(\sigma (c),\sigma (d)))$. By similar arguments, the second connected component implies $\tau _{S}({{\,\mathrm{lca}\,}}_S(\sigma (c),\sigma (d))) > \tau _{S}({{\,\mathrm{lca}\,}}_S(\sigma (a),\sigma (b)))$; a contradiction. These examples emphasize that LDT graphs constrain the relaxed scenarios, but are far from determining them.

## Horizontal gene transfer and fitch graphs

### HGT-labeled trees and rs-Fitch graphs

As alluded to in the introduction, the LDT graphs are intimately related with horizontal gene transfer. To formalize this connection we first define transfer edges. These will then be used to encode Walter Fitch’s concept of xenologous gene pairs (Fitch 2000; Darby et al. 2017) as a binary relation, and thus, the edge set of a graph.

#### Definition 14

Let ${\mathcal {S}}= (T,S,\sigma ,\mu ,\tau _{T},\tau _{S})$ be a relaxed scenario. An edge (*u*, *v*) in *T* is a *transfer edge* if $\mu (u)$ and $\mu (v)$ are incomparable in *S*. The *HGT-labeling* of *T* in ${\mathcal {S}}$ is the edge labeling $\lambda _{{\mathcal {S}}}: E(T)\rightarrow \{0,1\}$ with $\lambda (e)=1$ if and only if *e* is a transfer edge.

The vertex *u* in *T* thus corresponds to an HGT event, with *v* denoting the subsequent event, which now takes place in the “recipient” branch of the species tree. Note that $\lambda _{{\mathcal {S}}}$ is completely determined by ${\mathcal {S}}$. In general, for a given a gene tree *T*, HGT events correspond to a labeling or coloring of the edges of *T*.

#### Definition 15

(*Fitch graph*) Let $(T,\lambda )$ be a tree *T* together with a map $\lambda :E(T)\rightarrow \{0,1\}$. The *Fitch graph* $\digamma (T,\lambda ) = (V,E)$ has vertex set $V:=L(T)$ and edge set

$$\begin{aligned} E :=\{xy \mid x,y\in L,&\text { the unique path connecting } x \text { and } y \text { in } T \\&\text { contains an edge } e \text { with } \lambda (e)=1. \} \end{aligned}$$

By definition, Fitch graphs of 0/1-edge-labeled trees are loopless and undirected. We call edges *e* of $(T,\lambda )$ with label $\lambda (e)=1$ also 1-edges and, otherwise, 0-edges.

#### Remark 2

Fitch graphs as defined here have been termed *undirected* Fitch graphs (Hellmuth et al. 2018), in contrast to the notion of the *directed* Fitch graphs of 0/1-edge-labeled trees studied e.g. in Geiß et al. (2018) and Hellmuth and Seemann (2019).

#### Proposition 5

(Hellmuth et al. 2018; Zverovich 1999) The following statements are equivalent.

1. *G* is the Fitch graph of a 0/1-edge-labeled tree.
2. *G* is a complete multipartite graph.
3. *G* does not contain $K_2+K_1$ as an induced subgraph.

#### Definition 16

(*rs-Fitch graph*) Let ${\mathcal {S}}= (T,S,\sigma ,\mu ,\tau _{T},\tau _{S})$ be a relaxed scenario with HGT-labeling $\lambda _{{\mathcal {S}}}$. We call the vertex colored graph $(\digamma ({\mathcal {S}}),\sigma ) :=(\digamma (T,\lambda _{{\mathcal {S}}}),\sigma )$ the *Fitch graph of the scenario* ${\mathcal {S}}$.

A vertex colored graph $(G,\sigma )$ is a *relaxed scenario Fitch graph* (*rs-Fitch graph*) if there is a relaxed scenario ${\mathcal {S}}= (T,S,\sigma ,\mu ,\tau _{T},\tau _{S})$ such that $G = \digamma ({\mathcal {S}})$.

**Fig. 5:**
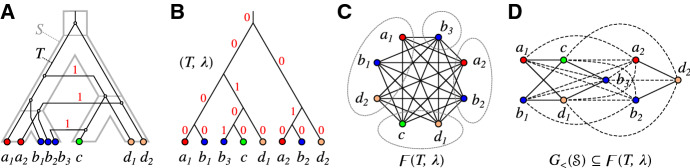
**A** The relaxed scenario ${\mathcal {S}}=(T,S,\sigma ,\mu ,\tau _{T},\tau _{S})$ as already shown in Fig. 1. **B** A 0/1-edge-labeled tree $(T,\lambda )$ satisfying $\lambda =\lambda _{{\mathcal {S}}}$. **C** The corresponding Fitch graph $\digamma (T,\lambda )$ drawn in a layout that emphasizes the property that $\digamma (T,\lambda )$ is a complete multipartite graph. Independent sets are circled. **D** An alternative layout as in Fig. 1 (top row) that emphasizes the relationship $G_{_{<}}({\mathcal {S}})\subseteq \digamma ({\mathcal {S}})=\digamma (T,\lambda )$ (cf. Theorem 4 below). Edges that are not present in $G_{_{<}}({\mathcal {S}})$ are drawn as dashed lines

Figure 5 shows that rs-Fitch graphs are not necessarily properly colored. A subtle difficulty arises from the fact that Fitch graphs of 0/1-edge-labeled trees are defined without a reference to the vertex coloring $\sigma$, while the rs-Fitch graph is vertex colored. This together with Proposition 5 implies

#### Observation 1

If $(G,\sigma )$ is an rs-Fitch graph then *G* is a complete multipartite graph.

The “converse” of Observation 1 is not true in general, as we shall see in Theorem 6 below. If, however, the coloring $\sigma$ can be chosen arbitrarily, then every complete multipartite graph *G* can be turned into an rs-Fitch graph $(G,\sigma )$ as shown in Proposition 6.

#### Proposition 6

If *G* is a complete multipartite graph, then there exists a relaxed scenario ${\mathcal {S}}=(T,S,\sigma ,\mu ,\tau _{T},\tau _{S})$ such that $(G,\sigma )$ is an rs-Fitch graph.

Although every complete multipartite graph can be colored in such a way that it becomes an rs-Fitch graph (cf. Proposition 6), there are colored, complete multipartite graphs $(G,\sigma )$ that are not rs-Fitch graphs, i.e., that do not derive from a relaxed scenario (cf. Theorem 6). We summarize this discussion in the following

#### Observation 2

There are (planted) 0/1-edge labeled trees $(T,\lambda )$ and colorings $\sigma :L(T)\rightarrow M$ such that there is no relaxed scenario ${\mathcal {S}}= (T,S,\sigma ,\mu ,\tau _{T},\tau _{S})$ with $\lambda =\lambda _{{\mathcal {S}}}$.

**Fig. 6:**
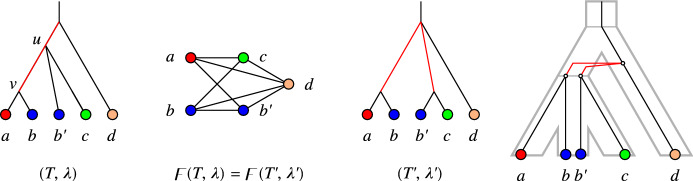
0/1-edge-labeled tree $(T,\lambda )$ for which no relaxed scenario exists such that $(T,\lambda ) = (T,\lambda _{{\mathcal {S}}})$ (see Example 1). Red edges indicates 1-labeled edges. Nevertheless for $\digamma :=\digamma (T,\lambda )$ there is an alternative tree $(T',\lambda ')$ for which a relaxed scenario ${\mathcal {S}}= (T',S,\sigma ,\mu ,\tau _{T},\tau _{S})$ exists (right) such that $\digamma = \digamma (T',\lambda ') = \digamma ({\mathcal {S}})$

A subtle—but important—observation is that trees $(T,\lambda )$ with coloring $\sigma$ for which Observation 2 applies may still encode an rs-Fitch graph $(\digamma (T,\lambda ),\sigma )$, see Example 1 and Fig. 6. The latter is due to the fact that $\digamma (T,\lambda ) = \digamma (T',\lambda ')$ may be possible for a different tree $(T',\lambda ')$ for which there is a relaxed scenario ${\mathcal {S}}' = (T',S,\sigma ,\mu ,\tau _{T},\tau _{S})$ with $\lambda ' = \lambda _{{\mathcal {S}}}$. In this case, $(\digamma (T,\lambda ),\sigma ) = (\digamma ({\mathcal {S}}'),\sigma )$ is an rs-Fitch graph. We shall briefly return to these issues in the discussion Sect. 8.

#### Example 1

Consider the planted edge-labeled tree $(T,\lambda )$ shown in Fig. 6 with leaf set $L=\{a,b,b',c,d\}$, together with a coloring $\sigma$ where $\sigma (b)=\sigma (b')$ and $\sigma (a), \sigma (b), \sigma (c), \sigma (d)$ are pairwise distinct.

Assume, for contradiction, that there is a relaxed scenario ${\mathcal {S}}= (T,S,\sigma ,\mu ,\tau _{T},\tau _{S})$ with $(T,\lambda ) = (T,\lambda _{{\mathcal {S}}})$. Hence, $\mu (v)$ and $\mu (b)=\sigma (b)$ as well as $\mu (u)$ and $\mu (b')=\sigma (b)$ must be comparable in *S*. Therefore, $\mu (u)$ and $\mu (v)$ must both be comparable to $\sigma (b)$ and thus, they are located on the path from $\rho _S$ to $\sigma (b)$. But this implies that $\mu (u)$ and $\mu (v)$ are comparable in *S*; a contradiction, since then $\lambda _{{\mathcal {S}}}(u,v) = 0\ne \lambda (u,v) = 1$.

### LDT graphs and rs-Fitch graphs

We proceed to investigate to what extent an LDT graph provides information about an rs-Fitch graph. As we shall see in Theorem 5 there is indeed a close connection between rs-Fitch graphs and LDT graphs. We start with a useful relation between the edges of rs-Fitch graphs and the reconciliation maps $\mu$ of their scenarios.

#### Lemma 13

Let $\digamma ({\mathcal {S}})$ be an rs-Fitch graph for some relaxed scenario ${\mathcal {S}}$. Then, $ab\notin E(\digamma ({\mathcal {S}}))$ implies that ${{\,\mathrm{lca}\,}}_S(\sigma (a),\sigma (b)) \preceq _S \mu ({{\,\mathrm{lca}\,}}_T(a,b))$.

The next result shows that a subset of transfer edges can be inferred immediately from LDT graphs:

#### Theorem 4

If $(G,\sigma )$ is an LDT graph, then $G\subseteq \digamma ({\mathcal {S}})$ for all relaxed scenarios ${\mathcal {S}}$ that explain $(G,\sigma )$.

Since we only have that *xy* is an edge in $\digamma ({\mathcal {S}})$ if the path connecting *x* and *y* in the tree *T* of ${\mathcal {S}}$ contains a transfer edge, Theorem 4 immediately implies

#### Corollary 6

For every relaxed scenario ${\mathcal {S}}=(T,S,\sigma ,\mu ,\tau _{T},\tau _{S})$ without transfer edges, it holds that $E(G_{_{<}}({\mathcal {S}})) = \emptyset$.

**Fig. 7:**
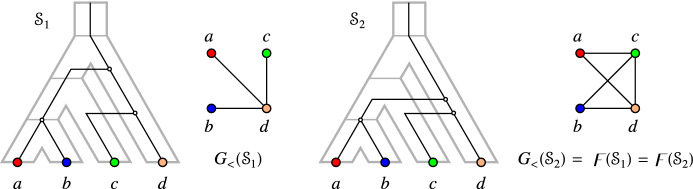
Two relaxed scenarios ${\mathcal {S}}_1$ and ${\mathcal {S}}_2$ with the same rs-Fitch graph $\digamma = \digamma ({\mathcal {S}}_1)=\digamma ({\mathcal {S}}_2)$ (right) and different LDT graphs $G_{_{<}}({\mathcal {S}}_1)\ne \digamma$ and $G_{_{<}}({\mathcal {S}}_2)=\digamma$

Theorem 4 provides the formal justification for indirect phylogenetic approaches to HGT inference that are based on the work of Lawrence and Hartl (1992), Clarke et al. (2002), and Novichkov et al. (2004) by showing that $(x,y)\in E(G_{_{<}}({\mathcal {S}}))$ can be explained only by HGT, irrespective of how complex the true biological scenario might have been. However, it does not cover all HGT events. Figure 7 shows that there are relaxed scenarios ${\mathcal {S}}$ for which $G_{_{<}}({\mathcal {S}}) \ne \digamma ({\mathcal {S}})$ even though $\digamma ({\mathcal {S}})$ is properly colored. Moreover, it is possible that an rs-Fitch graph $(G,\sigma )$ contains edges $xy\in E(G)$ with $\sigma (x)=\sigma (y)$. In particular, therefore, an rs-Fitch graph is not always an LDT graph.

It is natural, therefore, to ask whether for every properly colored Fitch graph there is a relaxed scenario ${\mathcal {S}}$ such that $G_{_{<}}({\mathcal {S}}) = \digamma ({\mathcal {S}})$. An affirmative answer is provided by

#### Theorem 5

The following statements are equivalent.

1. $(G,\sigma )$ is a properly colored complete multipartite graph.
2. There is a relaxed scenario ${\mathcal {S}}=(T,S,\sigma ,\mu ,\tau _{T},\tau _{S})$ with coloring $\sigma$ such that $G=G_{_{<}}({\mathcal {S}}) = \digamma ({\mathcal {S}})$.
3. $(G,\sigma )$ is complete multipartite and an LDT graph.
4. $(G,\sigma )$ is properly colored and an rs-Fitch graph.

In particular, for every properly colored complete multipartite graph $(G,\sigma )$ the triple set ${\mathfrak {S}}(G,\sigma )$ is compatible.

relaxed scenarios for which $(\digamma ({\mathcal {S}}),\sigma )$ is properly colored do not admit two members of the same gene family that are separated by a HGT event. While restrictive, such models are not altogether unrealistic. Proper coloring of $(\digamma ({\mathcal {S}}),\sigma )$ is, in particular, the case if every horizontal transfer is *replacing*, i.e., if the original copy is effectively overwritten by homologous recombination (Thomas and Nielsen 2005), see also (Choi et al. 2012) for a detailed case study in *Streptococcus*. As a consequence of Theorem 5, LDT graphs are sufficient to describe replacing HGT. However, the incidence rate of replacing HGT decreases exponentially with phylogenetic distance between source and target (Williams et al. 2012), and additive HGT becomes the dominant mechanism between phylogenetically distant organisms. Still, replacing HGTs may also be the result of additive HGT followed by a loss of the (functionally redundant) vertically inherited gene.

### rs-Fitch graphs with general colorings

In scenarios with additive HGT, the rs-Fitch graph is no longer properly colored and no-longer coincides with the LDT graph. Since not every vertex-colored complete multipartite graph $(G,\sigma )$ is an rs-Fitch graph (cf. Theorem 6), we ask whether an LDT $(G,\sigma )$ that is not itself already an rs-Fitch graph imposes constraints on the rs-Fitch graphs $(\digamma ({\mathcal {S}}),\sigma )$ that derive from relaxed scenarios ${\mathcal {S}}$ that explain $(G,\sigma )$. As a first step towards this goal, we aim to characterize rs-Fitch graphs, i.e., to understand the conditions imposed by the existence of an underlying scenario ${\mathcal {S}}$ on the compatibility of the collection of independent sets ${\mathscr {I}}$ of *G* and the coloring $\sigma$. As we shall see, these conditions can be explained in terms of an auxiliary graph that we introduce in a very general setting:

#### Definition 17

Let *L* be a set, $\sigma :L\rightarrow M$ a map and ${\mathscr {I}}=\{I_1,\dots , I_k\}$ a set of subsets of *L*. Then the graph ${\mathcal {A}}_{\digamma }(\sigma ,{\mathscr {I}})$ has vertex set *M* and edges *xy* if and only if $x\ne y$ and $x,y\in \sigma (I')$ for some $I'\in {\mathscr {I}}$.

By construction ${\mathcal {A}}_{\digamma }(\sigma ,\mathscr {I'})$ is a subgraph of ${\mathcal {A}}_{\digamma }(\sigma ,{\mathscr {I}})$ whenever $\mathscr {I'}\subseteq {\mathscr {I}}$. An extended version of Definition 17 that contains also an edge-labeling of ${\mathcal {A}}_{\digamma }(\sigma ,{\mathscr {I}})$ can be found in the Technical Part—this technical detail is not needed here. As it turns out, rs-Fitch graphs are characterized by the structure of their auxiliary graphs ${\mathcal {A}}_{\digamma }$ as shown in the next

#### Theorem 6

A graph $(G,\sigma )$ is an rs-Fitch graph if and only if (i) it is complete multipartite with independent sets ${\mathscr {I}}=\{I_1,\dots , I_k\}$, and (ii) if $k>1$, there is an independent set $I'\in {\mathscr {I}}$ such that ${\mathcal {A}}_{\digamma }(\sigma ,{\mathscr {I}}{\setminus }\{I'\})$ is disconnected.

As a consequence of Theorem 6, we obtain

#### Corollary 9

rs-Fitch graphs can be recognized in polynomial time.

As for LDT graphs, the property of being an rs-Fitch graph is hereditary.

#### Corollary 14

If $(G=(L,E),\sigma )$ is an rs-Fitch graph, then the colored vertex induced subgaph $(G[W],\sigma _{|W})$ is an rs-Fitch graph for all non-empty subsets $W\subseteq L$.

**Fig. 8:**
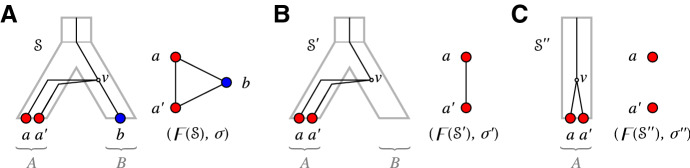
Shown are three distinct relaxed scenarios ${\mathcal {S}}$, ${\mathcal {S}}'$ and ${\mathcal {S}}''$ with corresponding rs-Fitch graphs. Here $\sigma ' = \sigma _{|\{a,a'\}}$ and $\sigma '' = \sigma _{|\{a,a'\},\{A\}}$ (cf. Definition 1). Putting $(G,\sigma ) = (\digamma ({\mathcal {S}}),\sigma )$, one can observe that $(G[\{a,a'\}], \sigma ') = (\digamma ({\mathcal {S}}'),\sigma ')$ is an rs-Fitch graph. In contrast, $\sigma ''$ is restricted to the “observable” part of species (consisting of *A* alone), and $(G[\{a,a'\}], \sigma '')$ is not an rs-Fitch graph, see text for further details

Note, however, that Corollary 14 is not satisfied if we restrict the codomain of $\sigma$ to the observable part of colors, i.e., if we consider $\sigma _{|W,\sigma (W)}:W \rightarrow \sigma (W)$ instead of $\sigma _{|W}:W\rightarrow M$, even if $\sigma$ is surjective. To see this consider the vertex colored graph $(G,\sigma )$ with $V(G)=\{a,a',b\}$, $E(G) = \{aa',ab,a'b\}$ and $\sigma :V(G)\rightarrow M = \{A,B\}$ where $\sigma (a) = \sigma (a')=A \ne \sigma (b)=B$. A possible relaxed scenario ${\mathcal {S}}$ for $(G,\sigma )$ is shown in Fig. 8A. The deletion of *b* yields $W=V(G){\setminus } \{b\} = \{a,a'\}$ and the graph $(G[W],\sigma _{|W})$ for which ${\mathcal {S}}'$ with HGT-labeling $\lambda _{{\mathcal {S}}'}$ as in Fig. 8B is a relaxed scenario that satisfies $G[W] = \digamma (T,\lambda _{{\mathcal {S}}'})$. However, if we restrict the codomain of $\sigma$ to obtain $\sigma _{|W,\{A\}}:\{a,a'\} \rightarrow \sigma (W) =\{A\}$, then there is no relaxed scenario ${\mathcal {S}}$ for which $G[W] = \digamma (T,\lambda _{{\mathcal {S}}})$, since there is only a single species tree *S* on $L(S)=\{A\}$ (Fig. 8C) that consists of the single edge $(0_T,A)$ and thus, $\mu (v)$ and $\mu (a)$ as well as $\mu (v)$ and $\mu (a')$ must be comparable in this scenario.

### Least resolved trees for Fitch graphs

It is important to note that the characterization of rs-Fitch graphs in Theorem 6 does not provide us with a characterization of rs-Fitch graphs that share a common relaxed scenario with a given LDT graph. As a potential avenue to address this problem we investigate the structure of least-resolved trees for Fitch graphs as possible source of additional constraints.

#### Definition 18

The edge-labeled tree $(T,\lambda )$ is *Fitch-least-resolved* w.r.t. $\digamma (T,\lambda )$, if for all trees $T'\ne T$ that are displayed by *T* and every labeling $\lambda '$ of $T'$ it holds that $\digamma (T,\lambda )\ne \digamma (T',\lambda ')$.

As shown in the Technical Part (Theorem 7), Fitch-least-resolved trees can be characterized in terms of their edge-labeling, a result that is very similar to the results for “directed” Fitch graphs of 0/1-edge-labeled trees in Geiß et al. (2018). As a consequence of this characterization, Fitch-least-resolved trees can be constructed in polynomial time. However, Fitch-least-resolved trees are far from being unique. In particular, Fitch-least-resolved trees are only of very limited use for the construction of relaxed scenarios ${\mathcal {S}}=(T,S,\sigma ,\mu ,\tau _{T},\tau _{S})$ from an underlying Fitch graph. In fact, even though $(G,\sigma )$ is an rs-Fitch graph, Example 3 in the Technical Part shows that it is possible that there is no relaxed scenario ${\mathcal {S}}=(T,S,\sigma ,\mu ,\tau _{T},\tau _{S})$ with HGT-labeling $\lambda _{{\mathcal {S}}}$ such that $(T,\lambda ) = (T,\lambda _{{\mathcal {S}}})$ for *any* of its Fitch-least-resolved trees $(T,\lambda )$.

## Editing problems

### Editing colored graphs to LDT graphs and Fitch graphs

Empirical estimates of LDT graphs from sequence data are expected to suffer from noise and hence to violate the conditions of Theorem 3. It is of interest, therefore, to consider the problem of correcting an empirical estimate $(G,\sigma )$ to the closest LDT graph. We therefore briefly investigate the usual three edge *modification* problems for graphs: *completion* only considers the insertion of edges, for *deletion* edges may only be removed, while solutions to the *editing* problem allow both insertions and deletions, see e.g. Burzyn et al. (2006).

#### Problem 1

(LDT-Graph-Modification (LDT-M))

*Input:* A colored graph $(G =(V,E),\sigma )$ and an integer *k*.
*Question:* Is there a subset $F\subseteq E$ such that $|F|\le k$ and $(G'=(V,E\star F),\sigma )$ is an LDT graph where $\star \in \{{\setminus }, \cup , \varDelta \}$?

We write LDT-E, LDT-C, LDT-D for the editing, completion, and deletion version of LDT-M. By virtue of Theorem 3, the LDT-M is closely related to the problem of finding a compatible subset ${\mathcal {R}}\subseteq {\mathfrak {S}}(G_{\mathcal {R}},\sigma )$ with maximum cardinality. The corresponding decision problem, MaxRTC, is known to be NP-complete (Jansson 2001, Thm. 1). In the technical part we prove

#### Theorem 9

LDT-M is NP-complete.

Even through at present it remains unclear whether rs-Fitch graphs can be estimated directly, the corresponding graph modification problems are at least of theoretical interest.

#### Problem 2

(rs-Fitch Graph-Modification (rsF-M))

*Input:* A colored graph $(G =(V,E),\sigma )$ and an integer *k*.
*Question:* Is there a subset $F\subseteq E$ such that $|F|\le k$ and $(G'=(V,E\star F),\sigma )$ is an rs-Fitch graph where $\star \in \{{\setminus }, \cup , \varDelta \}$?

As above, we write rsF-E, rsF-C, rsF-D for the editing, completion, and deletion version of rsF-M. Since rs-Fitch graphs are complete multipartite, their complements are disjoint unions of complete graphs. The problems rsF-M are thus closely related the cluster graph modification problems. Both Cluster Deletion and Cluster Editing are NP-complete, while Cluster Completion is polynomial (by completing each connected component to a clique, i.e., computing the transitive closure) (Shamir et al. 2004). We obtain

#### Theorem 10

rsF-C and rsF-E are NP-complete.

rsF-D remains open since the complement of the transitive closure of the complement of a colored graph $(G,\sigma )$ is not necessarily an rs-Fitch graph. This is in particular the case if $(G,\sigma )$ is complete multipartite but not an rs-Fitch graph.

### Editing LDT graphs to Fitch graphs

Putative LDT graphs $(G,\sigma )$ can be estimated directly from sequence (dis)similarity data. The most direct approach was introduced by Novichkov et al. (2004), where, for (reciprocally) most similar genes *x* and *y* from two distinct species $\sigma (x)=A$ and $\sigma (x)=B$, dissimilarities $\delta (x,y)$ between genes and dissimilarities $\varDelta (A,B)$ of the underlying species are compared under the assumption of a (gene family specific) clock-rate *r*, i.e., the expectation that orthologous gene pairs satisfy $\delta (x,y)\approx r \varDelta (A,B)$. In this setting, $xy\in E(G)$ if $\delta (x,y)< r \varDelta (A,B)$ at some level of statistical significance. The rate assumption can be relaxed to consider rank-order statistics. For fixed *x*, differences in the orders of $\delta (x,y)$ and $\varDelta (\sigma (x),\sigma (y))$ assessed by rank-order correlation measures have been used to identify *x* as HGT candidate e.g. Lawrence and Hartl (1992); Clarke et al. (2002). An interesting variation on the theme is described by Sevillya et al. (2020), who use relative synteny rather than sequence similarity for the same purpose. A more detailed account on estimating $(G,\sigma )$ will be given elsewhere.

**Fig. 9:**
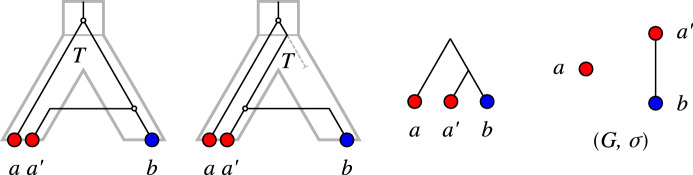
Two relaxed scenarios with *T* displaying the triple $a'b|a$ and explaining the same graph $(G,\sigma )$

In contrast, it seems much more difficult to infer a Fitch graph $(\digamma ,\sigma )$ directly from data. To our knowledge, no method for this purpose has been proposed in the literature. However, $(\digamma ,\sigma )$ is of much more direct practical interest because the independent sets of $\digamma$ determine the maximal HGT-free subsets of genes, which could be analyzed separately by better-understood techniques. In this section, we therefore focus on the aspects of $(\digamma ,\sigma )$ that are not captured by LDT graphs $(G,\sigma )$. In the light of the previous section, these are in particular non-replacing HGTs, i.e., HGTs that result in genes *x* and *y* in the same species $\sigma (x)=\sigma (y)$. In this case, $(\digamma ,\sigma )$ is no longer properly colored and thus $G\ne \digamma$. To get a better intuition on this case consider three genes *a*, $a'$, and *b* with $\sigma (a)=\sigma (a')\ne \sigma (b)$ with $ab\notin E(G)$ and $a'b\in E(G)$. By Lemma 7, the gene tree *T* of any explaining relaxed scenario displays the triple $a'b|a$. Fig. 9 shows two relaxed scenarios with a single HGT that explain this situation: In the first, we have $aa'\in E(\digamma )$, while the other implies $aa'\notin E(\digamma )$. Neither scenario is *a priori* less plausible than the other. Although the frequency of true homologous replacement via crossover decreases exponentially with the phylogenetic distance of donor and acceptor species (Williams et al. 2012), additive HGT with subsequent loss of one copy is an entirely plausible scenario.

A pragmatic approach to approximate $(\digamma ,\sigma )$ is therefore to consider the step from an LDT graph $(G,\sigma )$ to $(\digamma ,\sigma )$ as a graph modification problem. First we note that Algorithm 1 explicitly produces a relaxed scenario ${\mathcal {S}}$ and thus implies a corresponding gene tree $T_{{\mathcal {S}}}$ with HGT-labeling $\lambda _{{\mathcal {S}}}$, and thus an rs-Fitch graph $(\digamma ({\mathcal {S}}),\sigma )$. However, Algorithm 1 was designed primarily as proof device. It produces neither a unique relaxed scenario nor necessarily the most plausible or a most parsimonious one. Furthermore, both the LDT graph $(G,\sigma )$ and the desired rs-Fitch graph $(\digamma ,\sigma )$ are consistent with a potentially very large number of scenarios. It thus appears preferable to altogether avoid the explicit construction of scenarios at this stage.

Since every LDT graph $(G,\sigma )$ is explained by some ${\mathcal {S}}$, it is also a spanning subgraph of the corresponding rs-Fitch graph $(\digamma ({\mathcal {S}}),\sigma )$. The step from an LDT graph $(G,\sigma )$ to an rs-Fitch graph $(\digamma ,\sigma )$ can therefore be viewed as an edge-completion problem. The simplest variation of the problem is

#### Problem 3

(*Fitch graph completion*) Given an LDT graph $(G,\sigma )$, find a minimum cardinality set *Q* of possible edges such that $((V(G),E(G)\cup Q),\sigma )$ is a complete multipartite graph.

A close inspection of Problem 3 shows that the coloring is irrelevant in this version, and the actual problem to be solved is the problem Complete Multipartite Graph Completion with a cograph as input. We next show that this task can be performed in linear time. The key idea is to consider the complementary problem, i.e., the problem of deleting a minimum set of edges from the complementary cograph ${\overline{G}}$ such that the end result is a disjoint union of complete graphs. This is known as Cluster Deletion problem (Shamir et al. 2004), and is known to have a greedy solution for cographs (Gao et al. 2013).

#### Lemma 18

There is a linear-time algorithm to solve Problem 3 for every cograph *G*.

All maximum clique partitions of a cograph *G* have the same sequence of cluster sizes (Gao et al. 2013, Thm. 1). However, they are not unique as partitions of the vertex set *V*(*G*). Thus the minimal editing set *Q* that needs to be inserted into a cograph to reach a complete multipartite graphs will not be unique in general. In the Technical Part, we briefly sketch a recursive algorithm operating on the cotree of ${\overline{G}}$.

**Fig. 10:**
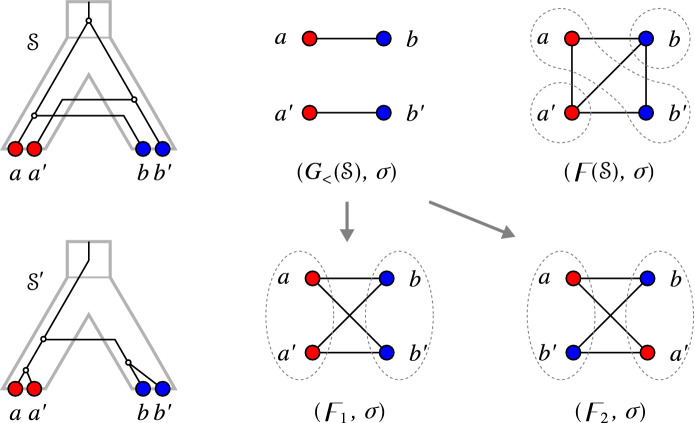
Upper panel: A relaxed scenario ${\mathcal {S}}$ with LDT graph $(G_{_{<}}({\mathcal {S}}),\sigma )$ and rs-Fitch graph $(\digamma ({\mathcal {S}}),\sigma )$. There are two minimum edge completion sets that yield the complete multipartite graphs $(\digamma _1,\sigma )$ and $(\digamma _2,\sigma )$ (lower part). By Theorem 6, $(\digamma _2,\sigma )$ is not an rs-Fitch graph. The graph $(\digamma _1,\sigma )$ is an rs-Fitch graph for the relaxed scenario ${\mathcal {S}}'$. However, $G_{_{<}}({\mathcal {S}})\ne G_{_{<}}({\mathcal {S}}')$ for all scenarios ${\mathcal {S}}'$ with $(\digamma ({\mathcal {S}}'),\sigma ) = (\digamma _1,\sigma )$. To see this, note that the gene tree $T=((a,b),(a',b'))$ in ${\mathcal {S}}$ is uniquely determined by application of Lemma 5 and 7. Assume that there is any edge-labeling $\lambda$ such that $\digamma (T,\lambda ) = \digamma _1$. The none-edges in $\digamma _1$ imply that along the two paths from *a* to $a'$ and *b* to $b'$ there is no transfer edge, that is, there cannot be any transfer edge in *T*; a contradiction

**Fig. 11:**
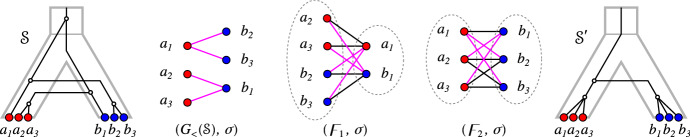
The LDT graph $(G_{_{<}}({\mathcal {S}}),\sigma )$ for the relaxed scenario ${\mathcal {S}}$ has a unique minimum edge completion set (as determined by full enumeration), resulting in the complete multipartite graph $(\digamma _1,\sigma )$. However, Theorem 6 implies that $(\digamma _1,\sigma )$ is not rs-Fitch graph. An edge completion set with more edges must be used to obtain an rs-Fitch graph, for instance $(\digamma _2,\sigma )$, which is explained by the scenario ${\mathcal {S}}'$

However, an optimal solution to Problem 3 with input $(G,\sigma )$ does not necessarily yield an rs-Fitch graph or an rs-Fitch graph $(\digamma ({\mathcal {S}}),\sigma )$ such that $G=G_{_{<}}({\mathcal {S}})$, see Fig. 10. In particular, there are LDT graphs $(G,\sigma )$ for which more edges need to be added to obtain an rs-Fitch graph than the minimum required to obtain a complete multipartite graph, see Fig. 11.

A more relevant problems for our purposes, therefore is

#### Problem 4

(*rs-Fitch graph completion*) Given an LDT graph $(G,\sigma )$ find a minimum cardinality set *Q* of possible edges such that $((V(G),E(G)\cup Q),\sigma )$ is an rs-Fitch graph.

The following, stronger version is what we ideally would like to solve:

#### Problem 5

(*strong rs-Fitch graph completion*) Given an LDT graph $(G,\sigma )$ find a minimum cardinality set *Q* of possible edges such that $\digamma = ((V(G),E(G)\cup Q),\sigma )$ is an rs-Fitch graph and there is a common relaxed scenario ${\mathcal {S}}$, that is, ${\mathcal {S}}$ satisfies $G = G_{_{<}}({\mathcal {S}})$ and $\digamma = \digamma ({\mathcal {S}})$.

The computational complexity of Problems 4 and 5 is unknown. We conjecture, however, that both are NP-hard. In contrast to the application of graph modification problems to correct possible errors in the originally estimated data, the minimization of inserted edges into an LDT graph lacks a direct biological interpretation. Instead, most-parsimonious solutions in terms of evolutionary events are usually of interest in biology. In our framework, this translates to

#### Problem 6

(*Min transfer completion*) Let $(G,\sigma )$ be an LDT graph and ${\mathbb {S}}$ be the set of all relaxed scenarios ${\mathcal {S}}$ with $G=G_{_{<}}({\mathcal {S}})$. Find a relaxed scenario ${\mathcal {S}}'\in {\mathbb {S}}$ that has a minimal number of transfer edges among all elements in ${\mathbb {S}}$ and the corresponding rs-Fitch graph $\digamma ({\mathcal {S}}')$.

One way to address this problem might be as follows: Find edge-completion sets for the given LDT graph $(G,\sigma )$ that minimize the number of independent sets in the resulting rs-Fitch graph $\digamma = ((V(G),E(G)\cup Q),\sigma )$. The intuition behind this idea is that, in this case, the number of pairs within the individual independent sets is maximized and thus, we get a maximized set of gene pairs without transfer along their connecting path in the gene tree. It remains an open question whether this idea always yields a solution for Problem 6.

## Simulation results

Evolutionary scenarios covering a wide range of HGT frequencies were generated with the simulation library AsymmeTree (Stadler et al. 2020). The tool generates a planted species tree *S* with time map $\tau _{S}$. A constant-rate birth-death process then generates a gene tree $({{\widetilde{T}}},{\widetilde{\tau _{T}}})$ with additional branching events producing copies at inner vertex *u* of *S* propagating to each descendant lineage of *u*. To model HGT events, a recipient branch of *S* is selected at random. The simulation is event-based in the sense that each node of the “true” gene tree other than the planted root is one of speciation, gene duplication, horizontal gene transfer, gene loss, or a surviving gene. Here, the lost as well as the surviving genes form the leaf set of ${{\widetilde{T}}}$.

**Fig. 12:**
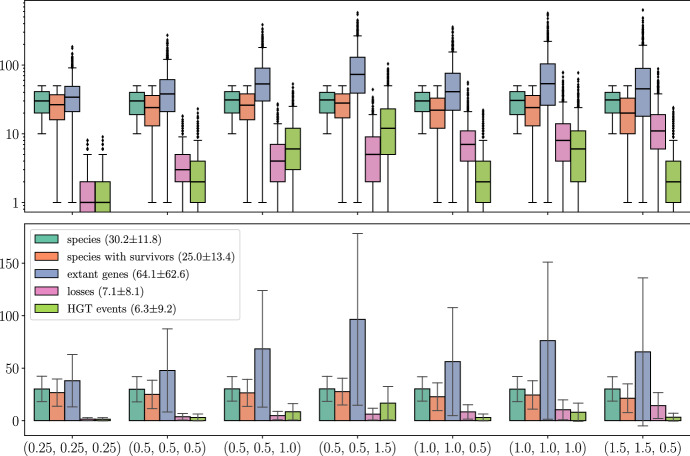
Top panel: Distribution of the numbers of species (i.e. species tree leaves), species thereof that contain at least one surviving genes, surviving genes in total (non-loss leaves in the gene trees), loss events (loss leaves), and horizontal transfer events (inner vertices that are HGT events). Bottom panel: Mean and standard deviation of these quantities. The numbers in the legend indicate the mean and standard deviation taken over all event rate combinations. The tuples on the horizontal axis give the rates for duplication, loss, and horizontal transfer

**Fig. 13:**
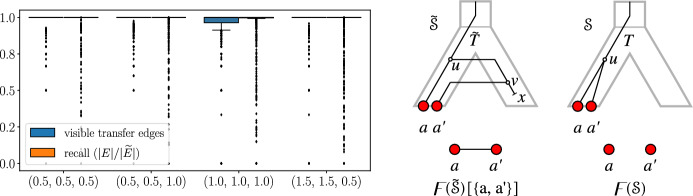
Left: Fraction of “visible” transfer edges among the “true” transfer edges in *T* in the simulated scenarios, i.e., the edges that correspond to a path in ${{\widetilde{T}}}$ containing at least one transfer edge w.r.t. $\widetilde{{\mathcal {S}}}$ (see also the explanation in the text). The tuples on the horizontal axis give the rates for duplication, loss, and horizontal transfer. Since $E:=E(\digamma ({\mathcal {S}})) \subseteq {\widetilde{E}} :=E(\digamma ({\widetilde{{\mathcal {S}}}})[L(T)])$, we also show the ratio $|E|/|{{\widetilde{E}}}|$. Right: A relaxed scenario ${\mathcal {S}}=(T,S,\sigma ,\mu ,\tau _{T},\tau _{S})$ with an “invisible” transfer edge $(u,a')$ (as determined by the knowledge of ${\widetilde{{\mathcal {S}}}}=(\widetilde{T},S,\sigma ,{\widetilde{\mu }},{\widetilde{\tau _{T}}},\tau _{S})$). In this example we have $\digamma ({\widetilde{{\mathcal {S}}}})[L(T)=\{a,a'\}] \ne \digamma ({\mathcal {S}})$

**Fig. 14:**
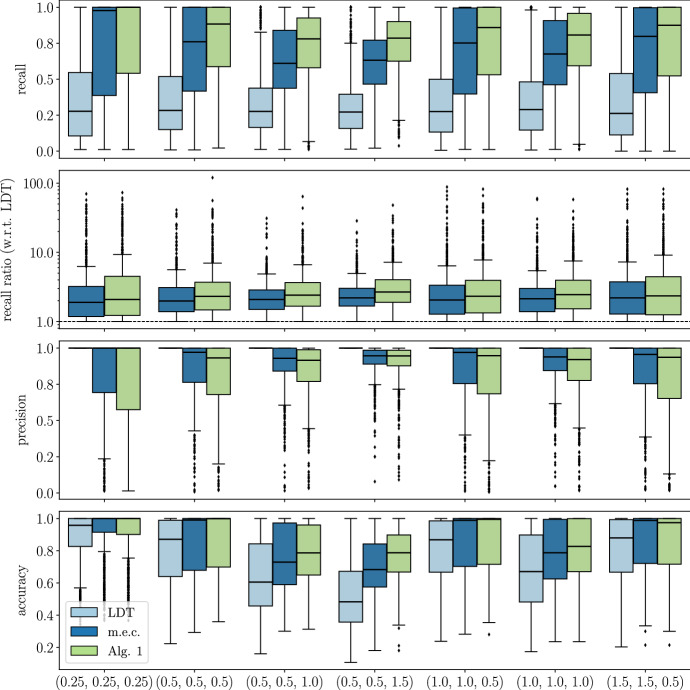
Xenologs inferred from LDT graphs. Only observable scenarios ${\mathcal {S}}$ whose LDT graph $(G_{_{<}}({\mathcal {S}}),\sigma )$ contains at least one edge are included (82.3% of all scenarios). The tuples on the horizontal axis give the rates for duplication, loss, and horizontal transfer. Top panel: Recall. Fraction of edges in $\digamma ({\mathcal {S}})$ represented in $G_{_{<}}({\mathcal {S}})$ (light blue). As an alternative, the fraction of edges in a “minimum edge completion” (m.e.c.) to the “closest” complete multipartite graph is shown in dark blue. We observe a substantial increase in the fraction of inferred edges. The Fitch graph $\digamma ({\mathcal {S}}')$ obtained from the scenario ${\mathcal {S}}'$ produced by Algorithm 1 with input $(G_{_{<}}({\mathcal {S}}),\sigma )$ yields an even better recall (light green). Second panel: Increase in the number of correctly inferred edges relative to the LDT graph $G_{_{<}}({\mathcal {S}})$. Third panel: Precision. In contrast to LDT graphs, which by Theorem 4 cannot contain false positive edges, this is not the case for the estimated Fitch graphs obtained as m.e.c. and by Algorithm 1. While false positive edges are typically rare, occasionally very poor estimates are observed. Bottom panel: Accuracy

We used the following parameter settings for AsymmeTree: Planted species trees with a number of leaves between 10 and 50 (randomly drawn in each scenario) were generated using the Innovation Model (Keller-Schmidt and Klemm 2012) and equipped with a time map as described in Stadler et al. (2020). Multifurcations were introduced into the species tree by contraction of inner edges with a common probability $p=0.2$ per edge to simulate. Gene trees therefore are also not binary in general. We used multifurcations to model the effects of limited phylogenetic resolution. Duplication and HGT events, however, always result in bifurcations in the gene tree ${{\widetilde{T}}}$. We considered different combinations of duplication, loss, and HGT event rates (indicated on the horizontal axis in Figs. 12, 13 and 14). For each combination of event rates, we simulated 1000 scenarios per event rate combination. Figure 12 summarizes basic statistics of the simulated data sets.

The simulation also determines the set of surviving genes $L\subseteq L({\widetilde{T}})$, the reconciliation map ${\widetilde{\mu }}:V({\widetilde{T}})\rightarrow V(S)\cup E(S)$ and the coloring $\sigma :L\rightarrow L(S)$ representing the species in which each surviving gene resides. From the true tree ${{\widetilde{T}}}$, the observable gene tree $T={\widetilde{T}}_{|L}$ is obtained by recursively removing leaves that correspond to loss events, i.e. $L({\widetilde{T}}){\setminus } L$, and suppressing inner vertices with a single child and setting $\tau _{T}(x)={\widetilde{\tau _{T}}}(x)$ and $\mu (x)={\widetilde{\mu }}(x)$ for all $x\in V(T)$. This defines a relaxed scenario ${\mathcal {S}}=(T,S,\sigma ,\mu ,\tau _{T},\tau _{S})$. From the scenario ${\mathcal {S}}$, we can immediately determine the associated HGT map $\lambda _{{\mathcal {S}}}$, the Fitch graph $\digamma ({\mathcal {S}})$, and the LDT graph $G_{_{<}}({\mathcal {S}})$. We also consider ${\widetilde{{\mathcal {S}}}}=({{\widetilde{T}}}, S,\sigma ,{\widetilde{\mu }},{\widetilde{\tau _{T}}},\tau _{S})$ which, from a formal point of view, is not a relaxed scenario, see Fig. 13. In this example, the gene-species association $\sigma :L \rightarrow L(S)$ is not a map for the entire leaf set $L({{\widetilde{T}}})$. Still, we can define the *true LDT graph* $G_{_{<}}({\widetilde{{\mathcal {S}}}})$ and the *true Fitch graph* $\digamma ({\widetilde{{\mathcal {S}}}})$ of ${\widetilde{{\mathcal {S}}}}$ in the same way as LDT graphs using Definitions 8, 9, and 16, respectively. Note that this does not guarantee that every true Fitch graph is also an rs-Fitch graph. The example in Fig. 13 shows, furthermore, that $\digamma ({\widetilde{{\mathcal {S}}}})[L] \ne \digamma ({\mathcal {S}})$ is possible. For the LDT graphs, on the other hand, we have $G_{_{<}}({\mathcal {S}}) = G_{_{<}}({\widetilde{{\mathcal {S}}}})$ because ${\widetilde{{\mathcal {S}}}}$ and ${\mathcal {S}}$ are based on the same time maps.

The distinction between the true graph $\digamma ({\widetilde{{\mathcal {S}}}})[L]$ and the rs-Fitch graph $\digamma ({\mathcal {S}})$ is closely related to the definition of transfer edges. So far, we only took into account transfer edges (*u*, *v*) in the (observable) gene trees *T*, for which *u* and *v* are mapped to incomparable vertices or edges of the species trees *S* (cf. Definition 14). Thus, given the knowledge of the relaxed scenario ${\mathcal {S}}=(T,S,\sigma ,\mu ,\tau _{T},\tau _{S})$, these transfer edges are in that sense “visible”. However, given ${\widetilde{{\mathcal {S}}}}=({{\widetilde{T}}}, S,\sigma ,{\widetilde{\mu }},{\widetilde{\tau _{T}}},\tau _{S})$, which still contains all loss branches, it is possible that a non-transfer edge in *T* corresponds to a path in ${{\widetilde{T}}}$ which contains a transfer edge w.r.t. ${\widetilde{{\mathcal {S}}}}$, i.e., some edge $(u,v)\in E({\widetilde{T}})$ such that $\widetilde{\mu }(u)$ and $\widetilde{\mu }(v)$ are incomparable in *S*. In particular, this is the case whenever a gene is transferred into some recipient branch followed by a back-transfer into the original branch and a loss in the recipient branch (see Fig. 13, right). Figure 13 shows that, in the majority of the simulated scenarios, the HGT information is preserved in the observable data. In fact, $\digamma ({\mathcal {S}})=\digamma ({\widetilde{{\mathcal {S}}}})$ in $86.7\%$ of simulated scenarios. Occasionally, however, we also encounter scenarios in which large fractions of the xenologous pairs are hidden from inference by the LDT-based approach.

In the following, we will only be concerned with estimating a Fitch graph $\digamma ({\mathcal {S}})$, i.e., the graph resulting from the “visible” transfer edges. These were edgeless in about $17.7\%$ of the observable scenarios ${\mathcal {S}}$ (all parameter combinations taken into account). In these cases the LDT and thus also the inferred Fitch graphs are edgeless. These scenarios were excluded from further analysis.

We first ask how well the LDT graph $G_{_{<}}({\mathcal {S}})$ approximates the Fitch graph $\digamma ({\mathcal {S}})$. As shown in Fig. 14, the recall is limited. Over a broad range of parameters, the LDT graph contains about a third of the xenologous pairs. This begs the question whether the solution of the editing Problem 3, obtained using the exact recursive algorithm detailed in Sect. C in the Technical Part, leads to a substantial improvement. We find that recall indeed increases substantially, at very moderate levels of false positives. The editing approach achieves a median precision of well above 90% in most cases and a median recall of at least 60%, it provides results that are at the very least encouraging. We find that minimal edge completion (Problem 3) already yields an rs-Fitch graph in the vast majority of cases (99.8%, scenarios of all parameter combinations taken into account), even if we restrict the color set to $M':=\sigma (L)$ (instead of *L*(*S*)) and thus force surjectivity of the coloring $\sigma$. We note that the original LDT graph and the minimal edge completion may not always be explained by a common scenario. This suggests that it will be worthwhile to consider the more difficult editing problems for rs-Fitch graphs with a relaxed scenario ${\mathcal {S}}$ that at the same time explains the LDT graph.

Algorithm 1 provides a means to obtain an rs-Fitch graph satisfying the latter constraint but without giving any guarantees for optimality in terms of a minimal edge completion. An implementation is available in the current release of the AsymmeTree package. For the rs-Fitch graphs $\digamma ({\mathcal {S}}')$ of the scenarios ${\mathcal {S}}'$ constructed by Algorithm 1 with $(G_{_{<}}({\mathcal {S}}),\sigma )$ as input, we observe another moderate increase of recall when compared with the minimal edge completion results. This comes, however, at the expense of a loss in precision. This is not surprising, since $\digamma ({\mathcal {S}}')$ by construction contains at least as many edges as any minimal edge completion of $G_{_{<}}({\mathcal {S}})$. Therefore, the number of both true positive and false positive edges in $\digamma ({\mathcal {S}}')$ can be expected to be higher, resulting in a higher recall and lower precision, respectively.

The recall is given by $TP / (TP + FN)$, and $|E(\digamma ({\mathcal {S}}))|= TP + FN$ in terms of true positives *TP* and false negatives *FN*. Moreover, $G_{_{<}}({\mathcal {S}})$ is a subgraph of the Fitch graphs $\digamma _{\text {m.e.c.}}$ and $\digamma ({\mathcal {S}}')$ inferred with editing or with Algorithm 1, respectively. The ratio $|E(\digamma ({\mathcal {S}})) \cap E(\digamma ^*)| / |E(\digamma ({\mathcal {S}}) \cap E(G_{_{<}}({\mathcal {S}})))|$ with $\digamma ^*\in \{\digamma _{\text {m.e.c.}}, \digamma ({\mathcal {S}}') \}$ therefore directly measures the increase in the number of correctly predicted xenologous pairs relative to the LDT. It is equivalent to the ratio of the respective recalls. By construction, the ratio is always $\ge 1$. This is summarized as the second panel in Fig. 14.

## Discussion and future directions

In this contribution, we have introduced *later-divergence-time (LDT) graphs* as a model capturing the subset of horizontal transfer detectable through the pairs of genes that have diverged later than their respective species. Within the setting of relaxed scenarios, LDT graphs $(G,\sigma )$ are exactly the properly colored cographs with a consistent triple set ${\mathfrak {S}}(G,\sigma )$. We further showed that LDT graphs describe a sufficient set of HGT events if and only if they are complete multipartite graphs. This corresponds to scenarios in which all HGT events are replacing. Otherwise, additional HGT events exist that separate genes from the same species. To better understand these, we investigated scenario-derived rs-Fitch graphs and characterized them as those complete multipartite graphs that satisfy an additional constraint on the coloring (expressed in terms of an auxiliary graph). Although the information contained in LDT graphs is not sufficient to unambiguously determine the missing HGT edges, we arrive at an efficiently solvable graph editing problem from which a “best guess” can be obtained. To our knowledge, this is the first detailed mathematical investigation into the power and limitation of an implicit phylogenetic method for HGT inference.

From a data analysis point of view, LDT graphs appear to be an attractive avenue to infer HGT in practice. While existing methods to estimate them from (dis)similarity data certainly can be improved, it is possible to use their cograph structure to correct the initial estimate in the same way as orthology data (Hellmuth et al. 2015). Although the LDT modification problems are NP-complete (Theorem 9), it does not appear too difficult to modify efficient cograph editing heuristics (Crespelle 2019; Hellmuth et al. 2020a) to accommodate the additional coloring constraints.

LDT graphs by themselves clearly do no contain sufficient information to completely determine a relaxed scenario. Additional information, e.g. a best match graph (Geiß et al. 2019, 2020a) will certainly be required. The most direct practical use of LDT information is to infer the Fitch graph, whose independent sets correspond to maximal HGT-free subsets of genes. These subsets can be analyzed separately (Hellmuth 2017) using recent results to infer gene family histories, including orthology relations from best match data (Geiß et al. 2020a; Schaller et al. 2021b). The main remaining unresolved question is whether the resulting HGT-free subtrees can be combined into a complete scenario using only relational information such as best match data. One way to attack this is to employ the techniques used by Lafond and Hellmuth (2020) to characterize the conditions under which a fully event-labled gene tree can be reconciled with unknown species trees. These not only resulted in an polynomial-time algorithm but also establishes additional constraints on the HGT-free subtrees. An alternative, albeit mathematically less appealing approach is to adapt classical phylogenetic methods to accommodate the HGT-free subtrees as constraints. We suspect that best match data can supply further, stringent constraints for this task. We will pursue this avenue elsewhere.

Several alternative routes can be followed to obtain Fitch graphs from LDT graphs. The most straightforward approach is to elaborate on the editing problems briefly discussed in Sect. 6. A natural question arising in this context is whether there are non-LDT edges that are shared by all minimal completion sets *Q*, and whether these “obligatory Fitch-edges” can be determined efficiently. A natural alternative is to modify Algorithm 1 to incorporate some form of cost function to favor the construction of biologically plausible scenarios. In a very different approach, one might also consider to use LDT graphs as constraints in probabilistic models to reconstruct scenarios, see e.g. Sjöstrand et al. (2014) and Khan et al. (2016).

Although we have obtained characterizations of both LDT graphs and rs-Fitch graphs, many open questions and avenues for future research remain.

*Reconciliation maps* The notion of *relaxed reconciliation maps* used here appears to be at least as general as alternatives that have been explored in the literature. It avoids the concurrent definition of event types and thus allows situations that may be excluded in a more restrictive setting. For example, relaxed scenarios may have two or more vertically inherited genes *x* and *y* in the same species with $u:={{\,\mathrm{lca}\,}}_T(x,y)$ mapping to a vertex of the species trees. In the usual interpretation, *u* correspond to a speciation event (by virtue of $\mu (u)\in V^0(S)$); on the other hand, the descendants *x* and *y* constitute paralogs in most interpretations. Such scenarios are explicitly excluded e.g. in Stadler et al. (2020). Lemma 3 suggests that relaxed scenarios are sufficiently flexible to make it possible to replace a scenario ${\mathcal {S}}$ that is “forbidden” in response to such inconsistent interpretations of events by an “allowed” scenario ${\mathcal {S}}'$ with the same $\sigma$ such that $G_{_{<}}({\mathcal {S}})=G_{_{<}}({\mathcal {S}}')$. Whether this is indeed true, or whether a more restrictive definition of reconciliation imposes additional constraints of LDT graphs will of course need to be checked in each case.

The restriction of a $\mu$-free scenario to a subset $L'$ of leaves of *T* and to a subset $M'$ of leaves of *S* is well defined as long as $\sigma (L')\subseteq M'$. One can also define a corresponding restriction of the reconciliation map $\mu$. Most importantly, the deletion of some leaves of *T* may leave inner vertices in *T* with only a single child, which are then suppressed to recover a phylogenetic tree. This replaces paths in *T* by single edges and thus affects the definition of the HGT map $\lambda _{{\mathcal {S}}}$ since a path in *T* that contains two adjacent vertices $u_1$, $u_2$ with incomparable images $\mu (u_1)$ and $\mu (u_2)$ may be replaced by an edge with comparable end points in the restricted scenario ${\mathcal {S}}'$. This means that HGT events may become invisible, and thus $\digamma ({\mathcal {S}}')$ is not necessarily an *induced* subgraph of $\digamma ({\mathcal {S}})$, but a subgraph that may lack additional edges. Note that this is in contrast to the *assumptions* made in the analysis of (directed) Fitch graphs of 0/1-edge-labeled graphs (Geiß et al. 2018; Hellmuth and Seemann 2019), where the information on horizontal transfers is inherited upon restriction of $(T,\lambda )$.

*Observability* The latter issue is a special case of the more general problem with *observability* of events. Conceptually, we assume that evolution followed a *true scenario* comprising discrete events (speciations, duplications, horizontal transfer, gene losses, and possibly other events such as hybridization which are not considered here). In computer simulations, of course we know this true scenario, as well as all event types. Gene loss not only renders some leaves invisible but also erases the evidence of all subtrees without surviving leaves. Removal of these vertices in general results in a non-phylogenetic gene tree that contains inner vertices with a single child. In the absence of horizontal transfer, this causes little problems and the *unobservable vertices* can be be removed as described in the previous paragraph, see e.g. Hernández-Rosales et al. (2012). The situation is more complicated with HGT. In Nøjgaard et al. (2018), an HGT-vertex is deemed observable if it has both a horizontally and a vertically inherited descendant. In our present setting, the scenario retains an HGT-edge by virtue of consecutive vertices in *T* with incomparable $\mu$-images, irrespective of whether an HGT-vertex is retained. This type of “vertex-centered” notion of xenology is explored further in Hellmuth et al. (2017). We suspect that these different points of view can be unified only when gene losses are represented explicitly or when gene and species tree trees are not required to be phylogenetic (with single-child vertices implicating losses). Either extension of the theory, however, requires a more systematic understanding of which losses need to be represented and what evidence can be acquired to “observe” them.

*Impact of orthology* Pragmatically, one would define two genes *x* and *y* to be *orthologs* if $\mu ({{\,\mathrm{lca}\,}}_T(x,y))\in V^0(S)$, i.e., if *x* and *y* are the product of a speciation event. Lemma 3 implies that there is always a scenario without any orthologs that explains a given LDT graph $(G,\sigma )$. In particular, therefore, $(G,\sigma )$ makes no implications on orthology. Conversely, however, orthology information is available and additional information on HGT might become available. In a situation akin to Fig. 9 (with the ancestral duplication moved down to the speciation), knowing that *a* and *b* are orthologs in the more restrictive sense that $\mu ({{\,\mathrm{lca}\,}}_T(a,b))={{\,\mathrm{lca}\,}}_S(\sigma (a),\sigma (b))$ excludes the r.h.s. scenario and implies that $a'$ is the horizontally inherited child, and therefore also that *a* and $a'$ are xenologs. This connection of orthology and xenology will be explored elsewhere.

*Other types of implicit phylogenetic information* LDT graphs are not the only conceivable type of accessible xenology information. A large class of methods is designed to assess whether a single gene is *a xenolog*, i.e., whether there is evidence that it has been horizontally inserted into the genome of the recipient species. The main subclasses evaluate nucleotide composition patterns, the phyletic distribution of best-matching genes, or combination thereof. A recent overview can be found e.g. in Sánchez-Soto et al. (2020). It remains an open question how this information can be utilized in conjunction with other types of HGT information, such as LDT graphs. It seems reasonable to expect that it can provide not only additional constraints to infer rs-Fitch graphs but also provides directional information that may help to infer the directed Fitch graphs studied by Geiß et al. (2018) and Hellmuth and Seemann (2019)). Complementarily, we may ask whether it is possible to gain direct information on HGT edges between pairs of genes in the same genome, and if so, what needs to be measured to extract this information efficiently.

We also have to leave open several mathematical questions. Regarding 0/1-edge labeled trees $(T,\lambda )$, it would be of interest to know whether there is always a relaxed scenario ${\mathcal {S}}= (T,S,\sigma ,\mu ,\tau _{T},\tau _{S})$ such that $(T,\lambda ) = (T,\lambda _{{\mathcal {S}}})$ for a suitable choice of $\sigma$. Elaborating on Theorem 5, it would be interesting to characterize the leaf colorings $\sigma$ for $(T,\lambda )$ such that there is a relaxed scenario ${\mathcal {S}}$ with $\digamma (T,\lambda ) = \digamma ({\mathcal {S}})$.

## Figures and Tables

**Fig. 15:**
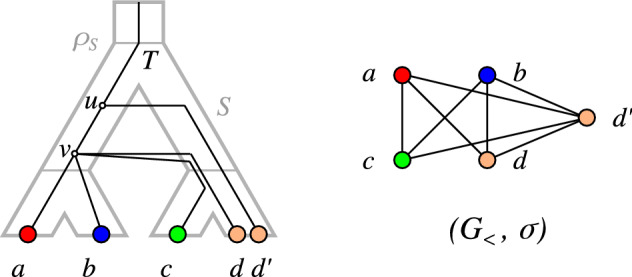
Left a relaxed scenario ${\mathcal {S}}=(T,S,\sigma ,\mu ,\tau _{T},\tau _{S})$ with corresponding graph $(G_{_{<}}({\mathcal {S}}),\sigma )$ (right). For $(G_{_{<}}({\mathcal {S}}),\sigma )$ there is no relaxed scenario $\widetilde{{\mathcal {S}}} = (T,S,\sigma ,{\widetilde{\mu }},{\widetilde{\tau _{T}}},\tau _{S})$ such that $G_{_{<}}(\widetilde{{\mathcal {S}}})=G_{_{<}}({\mathcal {S}})$ and for all distinct $x,y\in L(T)$ with $xy\notin E(G_{_{<}}({\mathcal {S}}))$ it holds that ${\widetilde{\mu }}({{\,\mathrm{lca}\,}}_T(x,y))\succ _S{{\,\mathrm{lca}\,}}_S(\sigma (x),\sigma (y))$, see Example 2

**Fig. 16:**
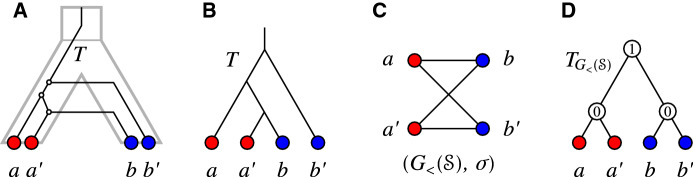
A relaxed scenario ${\mathcal {S}}$ (**A**) with gene tree *T* (**B**) and its associated graph $(G_{_{<}}({\mathcal {S}}),\sigma )$ (**C**). The discriminating cotree $T_{G_{_{<}}({\mathcal {S}})}$ (**D**) is not displayed by *T*

**Fig. 17:**
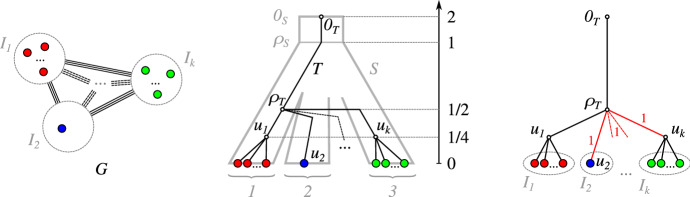
Construction in the proof of Proposition 6

**Fig. 18:**
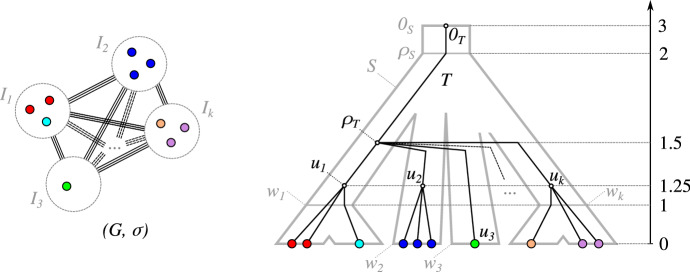
Construction of the relaxed scenario ${\mathcal {S}}$ in the proof of Theorem 5

**Fig. 19:**
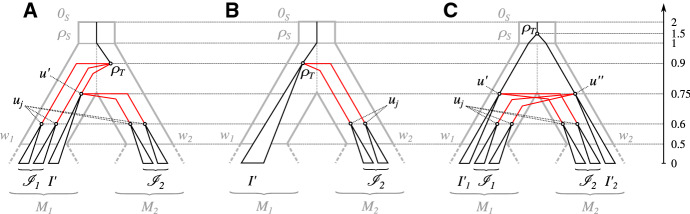
Illustration of the relaxed scenario constructed in the *if*-direction of the proof of Theorem 6. For Cases (a) and (c), only the situation in which a vertex $u'$ and $u''$, resp., is necessary is shown. Otherwise, the single element in $I'$, $I'_1$ or $I'_2$ would be a child of the root $\rho _T$. Moreover, the vertices $u_j$ are drawn under the assumption that $|I_j|>1$. Otherwise, there are identified with the single leaf in $I_j$

**Fig. 20:**
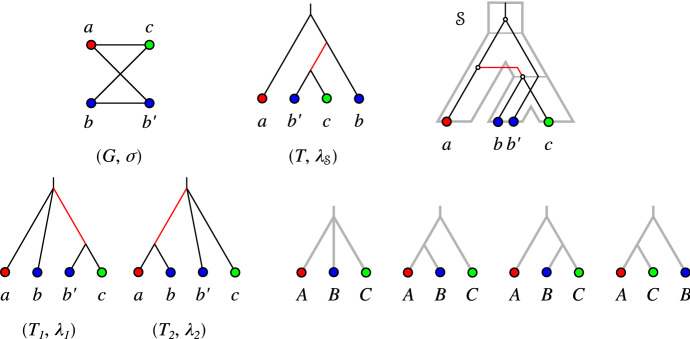
An rs-Fitch graph $(G,\sigma )$ and a possible relaxed scenario ${\mathcal {S}}=(T,S,\sigma ,\mu ,\tau _{T},\tau _{S})$ with $G = \digamma (T,\lambda _{{\mathcal {S}}})$. For the planted versions $(T_1,\lambda _1)$ and $(T_2,\lambda _2)$ of the Fitch-least-resolved trees of $(G,\sigma )$ there is no relaxed scenario ${\mathcal {S}}$ such that $(T_i,\lambda _i) = (T_i,\lambda _{{\mathcal {S}}})$, $i\in \{1,2\}$. Red edges indicate 1-labeled (i.e., transfer) edges. See Example 3 for further details

## Later-divergence-time graphs

### LDT graphs and evolutionary scenarios

In the absence of horizontal gene transfer, the last common ancestor of two species *A* and *B* should mark the latest possible time point at which two genes *a* and *b* residing in $\sigma (a)=A$ and $\sigma (b)=B$, respectively, may have diverged. Situations in which this constraint is violated are therefore indicative of HGT.

#### Definition 7

(${\mu }$*-free scenario*) Let *T* and *S* be planted trees, $\sigma :L(T)\rightarrow L(S)$ be a map and $\tau _{T}$ and $\tau _{S}$ be time maps of *T* and *S*, respectively, such that $\tau _{T}(x) = \tau _{S}(\sigma (x))$ for all $x\in L(T)$. Then, ${\mathcal {T}}=(T,S,\sigma ,\tau _{T},\tau _{S})$ is called a $\mu$-*free scenario*.

The condition that $\tau _{T}(x) = \tau _{S}(\sigma (x))$ for all $x\in L(T)$ is mostly a technical convenience that makes $\mu$-free scenarios easier to interpret. Nevertheless, by Lemma 1, given the time map $\tau _{S}$, one can easily construct a time map $\tau _{T}$ such that $\tau _{T}(x) = \tau _{S}(\sigma (x))$ for all $x\in L(T)$. In particular, when constructing relaxed scenarios explicitly, we may simply choose $\tau _{T}(u)=0$ and $\tau _{S}(x)=0$ as common time for all leaves $u\in L(T)$ and $x\in L(S)$.

#### Definition 8

(*LDT graph*) For a $\mu$-free scenario ${\mathcal {T}}=(T,S,\sigma ,\tau _{T},\tau _{S})$, we define $G_{_{<}}({\mathcal {T}}) = G_{_{<}}(T,S,\sigma ,\tau _{T},\tau _{S}) = (V,E)$ as the graph with vertex set $V:=L(T)$ and edge set

$$\begin{aligned} E :=\{ab\mid a,b\in L(T), \tau _{T}({{\,\mathrm{lca}\,}}_T(a,b))<\tau _{S}({{\,\mathrm{lca}\,}}_S(\sigma (a),\sigma (b))). \} \end{aligned}$$

A vertex-colored graph $(G,\sigma )$ is a *later-divergence-time graph* (*LDT* graph), if there is a $\mu$-free scenario ${\mathcal {T}}=(T,S,\sigma ,\tau _{T},\tau _{S})$ such that $G=G_{_{<}}({\mathcal {T}})$. In this case, we say that ${\mathcal {T}}$ *explains* $(G,\sigma )$.

It is easy to see that the edge set of $G_{_{<}}({\mathcal {T}})$ defines an *undirected* graph and that there are no edges of the form *aa*, since $\tau _{T}({{\,\mathrm{lca}\,}}_T(a,a)) = \tau _{T}(a) = \tau _{S}(\sigma (a)) =\tau _{S}({{\,\mathrm{lca}\,}}_S(\sigma (a),\sigma (a)))$. Hence $G_{_{<}}({\mathcal {T}})$ is a simple graph.

By definition, every relaxed scenario ${\mathcal {S}}=(T,S,\sigma ,\mu ,\tau _{T},\tau _{S})$ satisfies $\tau _{T}(x)=\tau _{S}(\sigma (x))$ all $x \in L(T)$. Therefore, removing $\mu$ from ${\mathcal {S}}$ yields a $\mu$-free scenario ${\mathcal {T}}=(T,S,\sigma ,\tau _{T},\tau _{S})$. Thus, we will use the following simplified notation.

#### Definition 9

We put $G_{_{<}}({\mathcal {S}}) :=G_{_{<}}(T,S,\sigma ,\tau _{T},\tau _{S})$ for a given relaxed scenario ${\mathcal {S}}=(T,S,\sigma ,\mu ,\tau _{T},\tau _{S})$ and the underlying $\mu$-free scenario $(T,S,\sigma ,\tau _{T},\tau _{S})$ and say, by slight abuse of notation, that ${\mathcal {S}}$ *explains* $(G_{_{<}}({\mathcal {S}}),\sigma )$.

#### Lemma 2

For every $\mu$-free scenario ${\mathcal {T}}=(T,S,\sigma ,\tau _{T},\tau _{S})$, there is a relaxed scenario ${\mathcal {S}}=(T,S,\sigma ,\mu ,{\widetilde{\tau _{T}}},{\widetilde{\tau _{S}}})$ for *T*, *S* and $\sigma$ such that $(G_{_{<}}({\mathcal {T}}),\sigma ) = (G_{_{<}}({\mathcal {S}}), \sigma )$.

#### Proof

Let ${\mathcal {T}}=(T,S,\sigma ,\tau _{T},\tau _{S})$ be a $\mu$-free scenario. In order to construct a relaxed scenario ${\mathcal {S}}=(T,S,\sigma ,\mu ,{\widetilde{\tau _{T}}},{\widetilde{\tau _{S}}})$ that satisfies $G_{_{<}}({\mathcal {S}})=G_{_{<}}({\mathcal {T}})$, we start with a time map ${\widetilde{\tau _{T}}}$ for *T* satisfying ${\widetilde{\tau _{T}}}(0_T)=\max (\tau _{T}(0_T),\tau _{S}(0_S))$ and ${\widetilde{\tau _{T}}}(v)=\tau _{T}(v)$ for all $v\in V(T){\setminus }\{0_T\}$. Correspondingly, we introduce a time map ${\widetilde{\tau _{S}}}$ for *S* such that ${\widetilde{\tau _{S}}}(0_S)=\max (\tau _{T}(0_T),\tau _{S}(0_S))$ and ${\widetilde{\tau _{S}}}(v)=\tau _{S}(v)$ for all $v\in V(S){\setminus }\{0_S\}$. By construction, we have $t_{\max ,T}:=\max \{\tau _{T}(v) \mid v\in V(T)\}=\tau _{T}(0_T)=\tau _{S}(0_S)$. Moreover, we have $t_{\min ,S}:=\min \{\tau _{S}(v) \mid v\in V(S)\} \le \min \{\tau _{T}(v) \mid v\in V(T)\}=:t_{\min ,T}$. To see this, we can choose $x\in V(T)$ such that $\tau _{T}(v)=t_{\min ,T}$. By the definition of time maps and minimality of $\tau _{T}(v)$, the vertex *x* must be a leaf. Hence, since ${\mathcal {T}}$ is a $\mu$-free scenario, we have $\tau _{T}(x)=\tau _{S}(\sigma (x))$ with $X:=\sigma (x)\in L(S)\subset V(S)$. Therefore, it must hold that $t_{\min ,S}\le t_{\min ,T}$. We now define $P:=\{p\in V(S)\cup E(S) \mid X\preceq _{S} p\}$, i.e., the set of all vertices and edges on the unique path in *S* from $0_S$ to the leaf *X*. Since $\tau _{S}(X)= t_{\min ,T} < t_{\max ,T} = \tau _{S}(0_S)$, we find, for each $v\in V(T)$, *either* a vertex $u\in P$ such that $\tau _{T}(v)=\tau _{S}(u)$ *or* an edge $(u,w)\in P$ such that $\tau _{S}(w)<\tau _{T}(v)<\tau _{S}(u)$. Hence, we can specify the reconciliation map $\mu$ by defining, for every $v\in V(T)$,

$$\begin{aligned} \mu (v) :={\left\{ \begin{array}{ll} 0_S &{}\text {if } v=0_T,\\ \sigma (v) &{}\text {if } v\in L(T),\\ u &{}\text {if there is some vertex } u\in P \text { with } \tau _{T}(v)=\tau _{S}(u),\\ (u,w) &{}\text {if there is some edge } (u,w)\in P \text { with } \tau _{S}(w)<\tau _{T}(v)<\tau _{S}(u). \end{array}\right. } \end{aligned}$$

For each $v\in V^0(T)$, exactly one of the two alternatives for *P* applies, hence $\mu$ is well-defined. It is now an easy task to verify that all conditions in Definitions 4 and 5 are satisfied for ${\mathcal {S}}=(T,S,\sigma ,\mu ,{\widetilde{\tau _{T}}},{\widetilde{\tau _{S}}})$ by construction. Hence, by Definition 6, ${\mathcal {S}}$ is a relaxed scenario.

It remains to show that $G_{_{<}}({\mathcal {T}})=G_{_{<}}({\mathcal {S}})$. Let $a,b\in L(T)$ be arbitrary. Clearly, neither ${{\,\mathrm{lca}\,}}_T(a,b)$ nor ${{\,\mathrm{lca}\,}}_S(\sigma (a),\sigma (b))$ equals the planted root $0_T$ or $0_S$, respectively. Since we have only changed the timing of the roots $0_T$ or $0_S$, we obtain $ab\in E(G_{_{<}}({\mathcal {S}}))$ if and only if ${\widetilde{\tau _{T}}}({{\,\mathrm{lca}\,}}_T(a,b)) = \tau _{T}({{\,\mathrm{lca}\,}}_T(a,b)) < {\widetilde{\tau _{S}}}({{\,\mathrm{lca}\,}}_S(\sigma (a),\sigma (b))) = \tau _{S}({{\,\mathrm{lca}\,}}_S(\sigma (a),\sigma (b)))$ if and only if $ab\in E(G_{_{<}}({\mathcal {T}}))$, which completes the proof. $\square$

#### Theorem 1

$(G,\sigma )$ is an LDT graph if and only if there is a relaxed scenario ${\mathcal {S}}= (T,S,\sigma ,\mu ,\tau _{T},\tau _{S})$ such that $(G,\sigma ) = (G_{_{<}}({\mathcal {S}}),\sigma )$.

#### Proof

By definition, $(G,\sigma )$ is an LDT graph for every relaxed scenario ${\mathcal {S}}$ with coloring $\sigma$ that satisfies $(G,\sigma ) = (G_{_{<}}({\mathcal {S}}),\sigma )$. Now suppose that $(G,\sigma )$ is an LDT graph. By definition, there is a $\mu$-free scenario ${\mathcal {T}}=(T,S,\sigma ,\tau _{T},\tau _{S})$ with coloring $\sigma$ such that $(G,\sigma )=(G_{_{<}}({\mathcal {T}}),\sigma )$. By Lemma 2, there is a relaxed scenario ${\mathcal {S}}=(T,S,\sigma ,\mu ,{\widetilde{\tau _{T}}},{\widetilde{\tau _{S}}})$ for *T*, *S* and $\sigma$ such that $(G,\sigma ) = (G_{_{<}}({\mathcal {S}}), \sigma )$. $\square$

#### Remark 3

From here on, we omit the explicit reference to Lemma 2 and Thm 1 and assume that the reader is aware of the fact that every LDT graph is explained by some relaxed scenario ${\mathcal {S}}$ and that for every $\mu$-free scenario ${\mathcal {T}}=(T,S,\sigma ,\tau _{T},\tau _{S})$, there is a relaxed scenario ${\mathcal {S}}$ for *T*, *S* and $\sigma$ such that $(G_{_{<}}({\mathcal {T}}),\sigma ) = (G_{_{<}}({\mathcal {S}}), \sigma )$.

We now derive some simple properties of $\mu$-free and relaxed scenarios. It may be surprising at first glance that “the speciation nodes”, i.e., vertices $u\in V^0(T)$ with $\mu (u)\in V(S)$ do not play a special role in determining LDT graphs.

#### Lemma 3

For every relaxed scenario ${\mathcal {S}}=(T,S,\sigma ,\mu ,\tau _{T},\tau _{S})$ there exists a relaxed scenario $\widetilde{{\mathcal {S}}} = (T,S,\sigma ,{\widetilde{\mu }},{\widetilde{\tau _{T}}},\tau _{S})$ such that $G_{_{<}}(\widetilde{{\mathcal {S}}})=G_{_{<}}({\mathcal {S}})$ and for all distinct $x,y\in L(T)$ with $xy\notin E(G_{_{<}}({\mathcal {S}}))$ holds ${\widetilde{\tau _{T}}}({{\,\mathrm{lca}\,}}_T(x,y))>\tau _{S}({{\,\mathrm{lca}\,}}_S(\sigma (x),\sigma (y)))$.

#### Proof

For the relaxed scenario ${\mathcal {S}}=(T,S,\sigma ,\mu ,\tau _{T},\tau _{S})$ we write $V^0(S):=V(S){\setminus } (L(S)\cup \{0_S\})$ and define

$$\begin{aligned} D_S&:=\{|\tau _{S}(y)-\tau _{S}(x)| :x,y\in V(S),\tau _{S}(x)\ne \tau _{S}(y)\} \text {,}\\ D_T&:=\{|\tau _{T}(y)-\tau _{T}(x)| :x,y\in V(T),\tau _{T}(x)\ne \tau _{T}(y)\} \text {, and} \\ D_{TS}&:=\{|\tau _{T}(x)-\tau _{S}(y)| :x\in V(T),\, y\in V(S), \tau _{T}(x)\ne \tau _{S}(y)\}. \end{aligned}$$

We have $D_S\ne \emptyset$ and $D_T\ne \emptyset$ since we do not consider empty trees, and thus, at least the “planted” edges $0_S\rho _S$ and $0_T\rho _T$ always exist. By construction, all values in $D_T$, $D_S$, and $D_{TS}$ are strictly positive. Now define

$$\begin{aligned} \epsilon :=\frac{1}{2}\min (D_{ST}\cup D_S\cup D_T). \end{aligned}$$

Since $D_S$ and $D_T$ are not empty, $\epsilon$ is well-defined and, by construction, $\epsilon >0$. Next we set, for all $v\in V(T)$,

$$\begin{aligned} \begin{aligned} {\widetilde{\tau _{T}}}(v)&:={\left\{ \begin{array}{ll} \tau _{T}(v)+\epsilon ,&{}\quad \text {if } v\in V^0(T)\\ \tau _{T}(v), &{}\quad \text {otherwise,} \end{array}\right. }\\ {\widetilde{\mu }}(v)&:={\left\{ \begin{array}{ll} ({{\,\mathrm{par}\,}}(x),x),&{}\quad \text {if } \mu (v) = x\in V^0(S)\\ \mu (v),&{}\quad \text {otherwise.} \end{array}\right. } \\ \end{aligned} \end{aligned}$$

#### Claim 1

$\widetilde{{\mathcal {S}}} :=(T,S,\sigma ,{\widetilde{\mu }},{\widetilde{\tau _{T}}},\tau _{S})$ is a relaxed scenario.

#### Proof

By construction, if $\mu (v)\in (L(S)\cup \{0_S\})$ and thus, $\mu (v)\notin V^0(S)$, $\mu (v)$ and ${\widetilde{\mu }}(v)$ coincide. Therefore, (G0) and (G1) are trivially satisfied for ${\widetilde{\mu }}$. In order to show (G2), we first note that ${\widetilde{\tau _{T}}}(v)= \tau _{T}(v) = \tau _{S}(\sigma (v))$ holds for all $v \in L(T)$ by Definition 4.

We next argue that ${\widetilde{\tau _{T}}}$ is a time map. To this end, let $x,y\in V(T)$ with $x\prec _T y$. Hence, $\tau _{T}(x)<\tau _{T}(y)$ and, in particular, $\tau _{T}(y)-\tau _{T}(x)\ge 2\epsilon$. Assume for contradiction that ${\widetilde{\tau _{T}}}(x) \ge {\widetilde{\tau _{T}}}(y)$. This implies ${\widetilde{\tau _{T}}}(x) = \tau _{T}(x)+\epsilon$ and ${\widetilde{\tau _{T}}}(y) =\tau _{T}(y)$, since $\tau _{T}(x)<\tau _{T}(y)$ and $\epsilon >0$ always implies $\tau _{T}(x)+\epsilon <\tau _{T}(y) +\epsilon$ and $\tau _{T}(x) <\tau _{T}(y) +\epsilon$. Therefore, ${\widetilde{\tau _{T}}}(y) - {\widetilde{\tau _{T}}}(x) = \tau _{T}(y)-(\tau _{T}(x) + \epsilon ) \ge \epsilon >0$ and thus, ${\widetilde{\tau _{T}}}(y) > {\widetilde{\tau _{T}}}(x)$; a contradiction.

We continue with showing that the two time maps ${\widetilde{\tau _{T}}}$ and $\tau _{S}$ are time-consistent w.r.t. $\widetilde{{\mathcal {S}}}$. To see that Condition (C1) is satisfied, observe that, by construction, ${\widetilde{\mu }}(v)\in V(S)$ does hold only in case $\mu (v)\notin E(S)\cup V^0(S)$ and thus, $\mu (v)\in L(S) \cup \{0_S\}$. In this case, ${\widetilde{\mu }}(v) = \mu (v)$ and since $\mu (v)$ satisfies (G1) we have $v\in L(T)\cup \{0_T\}$. Thus, $v\notin V^0(T)$ and, therefore, ${\widetilde{\tau _{T}}}(v) =\tau _{T}(v) = \tau _{S}(\mu (v))$. Therefore, Condition (C1) is satisfied.

Now consider Condition (C2). As argued above, ${\widetilde{\mu }}(v)\in E(S)$ holds for all $v\in V^0(T) = V(T){\setminus } (L(T)\cup \{0_T\})$. By construction, ${\widetilde{\tau _{T}}}(v) = \tau _{T}(v)+\epsilon$. There are two cases: $\mu (v)=x\in V^0(S)$, or $\mu (v)=(y,x)\in E(S)$ with $y = {{\,\mathrm{par}\,}}(x)$. The following arguments hold for both cases: We have ${\widetilde{\mu }}(v) = (y,x)\in E(S)$. Moreover, $\tau _{S}(x) \le \tau _{T}(v)< {\widetilde{\tau _{T}}}(v)$ since $\tau _{T}$ and $\tau _{S}$ satisfy (C1) and (C2). Furthermore, $\tau _{T}(v)<\tau _{S}(y)$ and, by construction, $\tau _{S}(y)-\tau _{T}(v)\ge 2\epsilon$. This immediately implies that $\tau _{S}(y) \ge \tau _{T}(v) + 2\epsilon = {\widetilde{\tau _{T}}}(v) + \epsilon > {\widetilde{\tau _{T}}}(v)$. In summary, $\tau _{S}(x)< \widetilde{\tau _{T}}(v) < \tau _{S}(y)$ whenever ${\widetilde{\mu }}(v) = (y,x)\in E(S)$. Therefore, Condition (C2) is satisfied for $\widetilde{{\mathcal {S}}}$. $\diamond$

#### Claim 2

$$E(G_{_{<}}({\mathcal {S}})) \subseteq E(G_{_{<}}(\widetilde{{\mathcal {S}}}))$$.

#### Proof

Let *xy* be an edge in $G_{_{<}}({\mathcal {S}})$ and thus $x\ne y$, and set $v_T:={{\,\mathrm{lca}\,}}_T(x,y)$ and $v_S:={{\,\mathrm{lca}\,}}_S(\sigma (x),\sigma (y))$. By definition, we have $\tau _{T}(v_T)<\tau _{S}(v_S)$. Therefore, we have $\tau _{S}(v_S)-\tau _{T}(v_T)\in D_{TS}$ and, hence, $\tau _{S}(v_S)-\tau _{T}(v_T)\ge 2\epsilon$. Since $x\ne y$, $v_T={{\,\mathrm{lca}\,}}_T(x,y)$ is an inner vertex of *T*. By construction, therefore, $\widetilde{\tau _{T}}(v_T)=\tau _{T}(v_T)+\epsilon$. The latter arguments together with the fact that $\tau _{S}$ remains unchanged imply that $\tau _{S}(v_S)-\widetilde{\tau _{T}}(v_T)\ge \epsilon >0$, and thus, $\widetilde{\tau _{T}}(v_T)<\tau _{S}(v_S)$. Therefore, we conclude that *xy* is an edge in $G_{_{<}}(\widetilde{{\mathcal {S}}})$. $\diamond$

It remains to show

#### Claim 3

For all distinct $x,y\in L(T)$ with $xy\notin E(G_{_{<}}({\mathcal {S}}))$, we have ${\widetilde{\tau _{T}}}({{\,\mathrm{lca}\,}}_T(x,y))>\tau _{S}({{\,\mathrm{lca}\,}}_S(\sigma (x),\sigma (y)))$.

#### Proof

Suppose $xy\notin E(G_{_{<}}({\mathcal {S}}))$ for two distinct $x,y\in L(T)$, and set $v_T:={{\,\mathrm{lca}\,}}_T(x,y)$ and $v_S:={{\,\mathrm{lca}\,}}_S(\sigma (x),\sigma (y))$. By definition, this implies $\tau _{T}(v_T)\ge \tau _{S}(v_S)$. Since $x\ne y$, we clearly have that $v_T={{\,\mathrm{lca}\,}}_T(x,y)$ is an inner vertex of *T*, and hence, $\widetilde{\tau _{T}}(v_T)=\tau _{T}(v_T)+\epsilon$. The latter two argument together with $\epsilon >0$ and the fact that $\tau _{S}$ remains unchanged imply that $\widetilde{\tau _{T}}(v_T)>\tau _{S}(v_S)$. $\diamond$

In particular, therefore, $xy\notin E(G_{_{<}}({\mathcal {S}}))$ implies that $xy\notin E(G_{_{<}}({\widetilde{{\mathcal {S}}}}))$ and therefore, $E(G_{_{<}}(\widetilde{{\mathcal {S}}}))\subseteq E(G_{_{<}}({\mathcal {S}}))$. Together with Claim  2 and the fact that both $G_{_{<}}({\mathcal {S}})$ and $G_{_{<}}({\widetilde{{\mathcal {S}}}})$ have vertex set *L*(*T*), we conclude that $G_{_{<}}({\mathcal {S}}) = G_{_{<}}(\widetilde{{\mathcal {S}}})$, which completes the proof. $\square$

Since the relaxed scenario $\widetilde{{\mathcal {S}}} = (T,S,\sigma ,{\widetilde{\mu }},{\widetilde{\tau _{T}}},\tau _{S})$ as constructed in the proof of Lemma 3 satisfies ${\widetilde{\mu }}(v)\notin V^0(S)$ we obtain

#### Corollary 1

For every relaxed scenario ${\mathcal {S}}=(T,S,\sigma ,\mu ,\tau _{T},\tau _{S})$ there exists a relaxed scenario $\widetilde{{\mathcal {S}}} = (T,S,\sigma ,{\widetilde{\mu }},{\widetilde{\tau _{T}}},\tau _{S})$ such that $G_{_{<}}(\widetilde{{\mathcal {S}}})=G_{_{<}}({\mathcal {S}})$ and ${\widetilde{\mu }}(v)\notin V^0(S)$ for all $v\in V(T)$.

Lemma 3, however, does not imply that one can always find a relaxed scenario with a reconciliation map ${\widetilde{\mu }}$ for given trees *T* and *S* satisfying ${\widetilde{\mu }}({{\,\mathrm{lca}\,}}_T(x,y))\succ _S{{\,\mathrm{lca}\,}}_S(\sigma (x),\sigma (y))$ for all distinct $x,y \in L(T)$ with $xy\notin E(G_{_{<}}({\mathcal {S}}))$, as shown in Example 2.

#### Example 2

Consider the LDT graph $(G_{_{<}}({\mathcal {S}}),\sigma )$ with corresponding relaxed scenario ${\mathcal {S}}$ as shown in Fig. 15. Note first that $v={{\,\mathrm{lca}\,}}_T(a,b)={{\,\mathrm{lca}\,}}_{T}(c,d)$ and $ab,cd\notin E(G_{_{<}})$. To satisfy both ${\widetilde{\mu }}(v)\succ _S {{\,\mathrm{lca}\,}}_S(\sigma (a),\sigma (b))$ and ${\widetilde{\mu }}(v)\succ _S {{\,\mathrm{lca}\,}}_S(\sigma (c),\sigma (d))$, we clearly need that $\widetilde{\mu }(v)\succeq _S \rho _S$, and thus ${\widetilde{\tau _{T}}}(v)\ge {\widetilde{\tau _{S}}}(\rho _S)$. However, $ad'\in E(G_{_{<}})$ and ${{\,\mathrm{lca}\,}}_{T}(a,d')=u$ imply that ${\widetilde{\tau _{T}}}(u)<\tau _{S}(\sigma (a),\sigma (d))=\tau _{S}(\rho _S)$. Hence, we obtain ${\widetilde{\tau _{T}}}(u)<\tau _{S}(\rho _S)\le {\widetilde{\tau _{T}}}(v)$; a contradiction to $(u,v)\in E(T)$ and $\widetilde{\tau _{T}}$ being a time map for *T*. Therefore, there is no relaxed scenario $\widetilde{{\mathcal {S}}} = (T,S,\sigma ,{\widetilde{\mu }},{\widetilde{\tau _{T}}},\tau _{S})$ such that $G_{_{<}}(\widetilde{{\mathcal {S}}})=G_{_{<}}({\mathcal {S}})$ and such that ${\widetilde{\mu }}({{\,\mathrm{lca}\,}}_T(x,y))\succ _S{{\,\mathrm{lca}\,}}_S(\sigma (x),\sigma (y))$ for all distinct $x,y\in L(T)$ with $xy\notin E(G_{_{<}}({\mathcal {S}}))$.

For the special case that the graph under consideration has no edges we have

#### Lemma 4

For an edgeless graph *G* and for any choice of  *T* and *S* with $L(T)=V(G)$ and $\sigma (L(T))=L(S)$ there is a relaxed scenario ${\mathcal {S}}=(T,S,\sigma ,\mu ,\tau _{T},\tau _{S})$ that satisfies $G = G_{_{<}}({\mathcal {S}})$.

#### Proof

Given *T* and *S* we construct a relaxed scenario as follows. Let $\tau _{S}$ be an arbitrary time map on *S*. Then we can choose $\tau _{T}$ such that $\tau _{S}(\rho _S)<\tau _{T}(u)<\tau _{S}(0_S)$ for all $u\in V^0(T)$. Each leaf $u\in L(T)$ then has a parent in *T* located above the last common ancestor $\rho _S$ of all species in which case $G_{_{<}}({\mathcal {S}})$ is edgeless. $\square$

Lemma 4 is reminiscent of the fact that for DL-only scenarios any given gene tree *T* can be reconciled with an arbitrary species tree as long as $\sigma (L(T))=L(S)$ (Guigó et al. 1996; Geiß et al. 2020a).

### Properties of LDT graphs

#### Proposition 3

Every LDT graph $(G,\sigma )$ is properly colored.

#### Proof

Let ${\mathcal {T}}=(T,S,\sigma ,\tau _{T},\tau _{S})$ be a $\mu$-free scenario such that $(G,\sigma ) = (G_{_{<}}({\mathcal {T}}),\sigma )$ and recall that every $\mu$-free scenario satisfies $\tau _{T}(x) = \tau _{S}(\sigma (x))$ for all $x\in L(T)$ with $\sigma (x)\in L(S)$. Let $a,b\in L(T)$ be distinct and suppose that $\sigma (a)=\sigma (b)=A$. Since *a* and *b* are distinct we have $a,b\prec _T {{\,\mathrm{lca}\,}}_T(a,b)$ and hence, by Definition 3, $\tau _{T}(a) < \tau _{T}({{\,\mathrm{lca}\,}}_T(a,b))$. This implies that $\tau _{T}(a) = \tau _{S}(A) = \tau _{S}({{\,\mathrm{lca}\,}}_S(A,A)) <\tau _{T}({{\,\mathrm{lca}\,}}_T(a,b))$. Therefore, $ab\notin E(G)$. Consequently, $ab\in E(G)$ implies $\sigma (a)\ne \sigma (b)$, which completes the proof. $\square$

Extending earlier work of Dekker (1986) and Bryant and Steel (1995) derived conditions under which two triples $r_1,r_2$ imply a third triple $r_3$ that must be displayed by any tree that displays $r_1,r_2$. In particular, we make frequent use of the following

#### Lemma 5

If a tree *T* displays *xy*|*z* and *zw*|*y* then *T* displays *xy*|*w* and *zw*|*x*. In particular $T_{|\{x,y,z,w\}} = ((x,y),(z,w))$ (in *Newick* format).

#### Definition 10

For every graph $G=(L,E)$, we define the set of triples on *L*

$$\begin{aligned} {\mathfrak {T}}(G) :=\{xy|z \; :x,y,z\in L \text { are pairwise distinct, } xy\in E,\; xz,yz\notin E\} \,. \end{aligned}$$

If *G* is endowed with a coloring $\sigma :L\rightarrow M$ we also define a set of color triples

$$\begin{aligned} {\mathfrak {S}}(G,\sigma ) :=\{\sigma (x)\sigma (y)|\sigma (z)\; :&x,y,z\in L,\, \sigma (x),\sigma (y),\sigma (z) \text { are pairwise distinct},\\&xz, yz\in E,\; xy\notin E\}. \end{aligned}$$

#### Lemma 6

If a graph $(G,\sigma )$ is an LDT graph then ${\mathfrak {S}}(G,\sigma )$ is compatible and *S* displays ${\mathfrak {S}}(G,\sigma )$ for every $\mu$-free scenario ${\mathcal {T}}=(T,S,\sigma ,\tau _{T},\tau _{S})$ that explains $(G,\sigma )$.

#### Proof

Suppose that $(G=(L,E),\sigma )$ is an LDT graph and let ${\mathcal {T}}=(T,S,\sigma ,\tau _{T},\tau _{S})$ be a $\mu$-free scenario that explains $(G,\sigma )$. In order to show that ${\mathfrak {S}}(G,\sigma )$ is compatible it suffices to show that *S* displays every triple in ${\mathfrak {S}}(G,\sigma )$.

Let $AB|C\in {\mathfrak {S}}(G,\sigma )$. By definition, *A*, *B*, *C* are pairwise distinct and there must be vertices $a,b,c\in L$ with $\sigma (a)=A$, $\sigma (b)=B$, and $\sigma (c)=C$ such that $ab \notin E$ and $bc,ac \in E$. First, $ab \notin E$ and $bc,ac \in E$ imply $\tau _{T}({{\,\mathrm{lca}\,}}_T(a,b))\ge \tau _{S}({{\,\mathrm{lca}\,}}_S(A,B))$, $\tau _{T}({{\,\mathrm{lca}\,}}_T(b,c))<\tau _{S}({{\,\mathrm{lca}\,}}_S(B,C))$, and $\tau _{T}({{\,\mathrm{lca}\,}}_T(a,c))<\tau _{S}({{\,\mathrm{lca}\,}}_S(A,C))$. Moreover, for any three vertices *a*, *b*, *c* in *T* it holds that $1 \le |\{{{\,\mathrm{lca}\,}}_T(a,b),{{\,\mathrm{lca}\,}}_T(a,c),{{\,\mathrm{lca}\,}}_T(b,c)\}| \le 2$.

Therefore we have to consider the following four cases: (1) $u:={{\,\mathrm{lca}\,}}_T(a,b)={{\,\mathrm{lca}\,}}_T(b,c)={{\,\mathrm{lca}\,}}_T(a,c)$, (2) $u:={{\,\mathrm{lca}\,}}_T(a,b)={{\,\mathrm{lca}\,}}_T(a,c)\ne {{\,\mathrm{lca}\,}}_T(b,c)$ and (3) $u:={{\,\mathrm{lca}\,}}_T(a,b)={{\,\mathrm{lca}\,}}_T(b,c)\ne {{\,\mathrm{lca}\,}}_T(a,c)$, (4) ${{\,\mathrm{lca}\,}}_T(a,b)\ne u:={{\,\mathrm{lca}\,}}_T(b,c)={{\,\mathrm{lca}\,}}_T(a,c)$. Note, for any three vertices *x*, *y*, *z* in *T*, ${{\,\mathrm{lca}\,}}_T(x,y)\ne {{\,\mathrm{lca}\,}}_T(x,z)={{\,\mathrm{lca}\,}}_T(y,z)$ implies that ${{\,\mathrm{lca}\,}}_T(x,y)\prec _T {{\,\mathrm{lca}\,}}_T(x,z)={{\,\mathrm{lca}\,}}_T(y,z)$. In Cases (1) and (2), we find $\tau _{S}({{\,\mathrm{lca}\,}}_S(A,C)) > \tau _{T}(u) \ge \tau _{S}({{\,\mathrm{lca}\,}}_S(A,B))$. Together with the fact that ${{\,\mathrm{lca}\,}}_S(A,C)$ and ${{\,\mathrm{lca}\,}}_S(A,B)$ are comparable in *S*, this implies that *AB*|*C* is displayed by *S*. In Case (3), we obtain $\tau _{S}({{\,\mathrm{lca}\,}}_S(B,C)) > \tau _{T}(u) \ge \tau _{S}({{\,\mathrm{lca}\,}}_S(A,B))$ and, by analogous arguments, *AB*|*C* is displayed by *S*. Finally, in Case (4), the tree *T* displays the triple *ab*|*c*. Thus, $\tau _{S}({{\,\mathrm{lca}\,}}_S(A,B))\le \tau _{T}({{\,\mathrm{lca}\,}}_T(a,b))< \tau _{T}(u) < \tau _{S}({{\,\mathrm{lca}\,}}_S(A,C))$. Again, *AB*|*C* is displayed by *S*. $\square$

The next lemma shows that induced $K_2+K_1$ subgraphs in LDT graphs implies triples that must be displayed by *T*.

#### Lemma 7

If $(G,\sigma )$ is an LDT graph, then ${\mathfrak {T}}(G)$ is compatible and *T* displays ${\mathfrak {T}}(G)$ for every $\mu$-free scenario ${\mathcal {T}}=(T,S,\sigma ,\tau _{T},\tau _{S})$ that explains $(G,\sigma )$.

#### Proof

Suppose that $(G=(L,E),\sigma )$ is an LDT graph and let ${\mathcal {T}}=(T,S,\sigma ,\tau _{T},\tau _{S})$ be a $\mu$-free scenario that explains $(G,\sigma )$. In order to show that ${\mathfrak {T}}(G)$ is compatible it suffices to show that *T* displays every triple in ${\mathfrak {T}}(G,\sigma )$.

Let $ab|c \in {\mathfrak {T}}(G)$. By definition, $a,b,c\in L(T)$ are distinct, and $ab\in E$ and $ac,bc\not \in E$. Since $ab \in E$, we have $A:=\sigma (a)\ne \sigma (b)=:B$ by Proposition 3.

There are two cases, either $\sigma (c)\in \{A,B\}$ or not. Suppose first that w.l.o.g. $\sigma (c)=A$. In this case, $ab \in E$ and $bc \notin E$ together imply $\tau _{T}({{\,\mathrm{lca}\,}}_T(a,b))<\tau _{S}({{\,\mathrm{lca}\,}}_S(A,B))\le \tau _{T}({{\,\mathrm{lca}\,}}_T(b,c))$. This and the fact that ${{\,\mathrm{lca}\,}}_T(a,b)$ and ${{\,\mathrm{lca}\,}}_T(b,c)$ are comparable in *T* implies that *T* displays *ab*|*c*.

Suppose now that $\sigma (c)=C\notin \{A,B\}$. We now consider the four possible topologies of $S'=S_{|ABC}$: (1) $S'$ is a star, (2) $S'=AB|C$, (3) $S'=AC|B$, and (4) $S'=BC|A$.

In Cases (1), (2) and (4), we have $\tau _{S}({{\,\mathrm{lca}\,}}_S(A,B)) \le \tau _{S}({{\,\mathrm{lca}\,}}_S(A,C))$, where equality holds only in Cases (1) and (4). This together with $ab \in E$ and $ac \notin E$ implies $\tau _{T}({{\,\mathrm{lca}\,}}_T(a,b))<\tau _{S}({{\,\mathrm{lca}\,}}_S(A,B)) \le \tau _{S}({{\,\mathrm{lca}\,}}_S(A,C)) \le \tau _{T}({{\,\mathrm{lca}\,}}_T(a,c))$. This and the fact that ${{\,\mathrm{lca}\,}}_T(a,b)$ and ${{\,\mathrm{lca}\,}}_T(a,c)$ are comparable in *T* implies that *T* displays *ab*|*c*. In Case (3), $ab \in E$ and $bc \notin E$ imply $\tau _{T}({{\,\mathrm{lca}\,}}_T(a,b))<\tau _{S}({{\,\mathrm{lca}\,}}_S(A,B)) = \tau _{S}({{\,\mathrm{lca}\,}}_S(B,C)) \le \tau _{T}({{\,\mathrm{lca}\,}}_T(b,c))$. By analogous arguments as before, *T* displays *ab*|*c*.

$$\square $$

We note, finally, that the Aho graph of the triple set $[{\mathfrak {T}}(G),L]$ in a sense recapitulates *G*. More precisely, we have:

#### Proposition 4

Let $(G=(L,E),\sigma )$ be a vertex-colored graph. If for all edges $xy\in E$ there is a vertex *z* such that $xz,yz\notin E$ (and thus, in particular, in case that *G* is disconnected), then $[{\mathfrak {T}}(G),L]=G$.

#### Proof

Clearly, the vertex sets of $[{\mathfrak {T}}(G),L]$ and *G* are the same, that is, *L*. Let $xy\in E$ and thus, we have $x\ne y$. There is a vertex $z\ne x,y$ in *G* with $xz,yz\notin E$ if and only if $xy|z\in {\mathfrak {T}}(G)$ and thus, if and only if *xy* is an edge in $[{\mathfrak {T}}(G),L]=G$. $\square$

#### Definition 11

For a vertex-colored graph $(G,\sigma )$, we will use the shorter notation $x_1-x_2-\dots -x_n$ and $X_1-X_2-\dots -X_n$ for a path $P_n$ that is induced by the vertices $\{x_i\mid 1\le i\le n\}$ with colors $\sigma (x_i)=X_i$, $1\le i\le n$ and edges $x_ix_{i+1}$, $1\le i\le n-1$.

#### Lemma 8

Every LDT graph $(G,\sigma )$ is a properly colored cograph.

#### Proof

Let ${\mathcal {T}}=(T,S,\sigma ,\tau _{T},\tau _{S})$ be a $\mu$-free scenario that explains $(G,\sigma )$. By Proposition 3, $(G,\sigma )$ is properly colored. To show that $G=(L,E)$ is a cograph it suffices to show that *G* does not contain an induced path on four vertices (cf. Proposition 2). Hence, assume for contradiction that *G* contains an induced $P_4$.

First we observe that for each edge *ab* in this $P_4$ it holds that $\sigma (a)\ne \sigma (b)$ since, otherwise, by Proposition 3, $ab\notin E$. Based on possible colorings of the $P_4$ w.r.t. $\sigma$ and up to symmetry, we have to consider four cases: (1) $A-B-C-D$, (2) $A-B-C-A$, (3) $A-B-A-C$ and (4) $A-B-A-B$.

In Case (1) the $P_4$ is of the form $a-b-c-d$ with $\sigma (a)=A$, $\sigma (b)=B$, $\sigma (c)=C$, $\sigma (d)=D$. By Lemma  6, the species tree *S* must display both *AC*|*B* and *BD*|*C*. Hence, by Lemma 5, $S_{|ABCD} = ((A,C),(B,D))$ in *Newick* format. Let $x :={{\,\mathrm{lca}\,}}_S(A,B,C,D) = \rho _{S_{|ABCD}}$. Note, *x* “separates” *A* and *C* from *B* and *D*. Now, $ab\in E$ and $ad\notin E$ implies that $\tau _{T}({{\,\mathrm{lca}\,}}_T(a,b))<\tau _{S}(x)\le \tau _{T}({{\,\mathrm{lca}\,}}_T(a,d))$. This and the fact that ${{\,\mathrm{lca}\,}}_T(a,b)$ and ${{\,\mathrm{lca}\,}}_T(a,d)$ are comparable in *T* implies that *T* displays *ab*|*d*. Similarly, $cd\in E$ and $ad\notin E$ implies that *T* displays *cd*|*a* is displayed by *T*. By Lemma 5, $T_{|abcd} = ((a,b),(c,d))$. Let $y :={{\,\mathrm{lca}\,}}_T(a,b,c,d) = \rho _{T_{|abcd}}$. Now, $bc\in E$, ${{\,\mathrm{lca}\,}}_T(b,c)=y$, and ${{\,\mathrm{lca}\,}}_S(B,C)=x$ implies $\tau _{T}(y)<\tau _{S}(x)$. This and ${{\,\mathrm{lca}\,}}_T(a,d)=y$ and ${{\,\mathrm{lca}\,}}_S(A,D)=x$ imply that $ad\in E$, and thus *a*, *b*, *c*, *d* do not induce a $P_4$ in *G*; a contradiction.

Case (2) can be directly excluded, since Lemma 6 implies that, in this case, *S* must display *AC*|*B* and *AB*|*C*; a contradiction.

Now consider Case (3), that is, the $P_4$ is of the form $a-b-a'-c$ with $\sigma (a)=\sigma (a')=A$, $\sigma (b)=B$ and $\sigma (c)=C$. By Lemma  6, the species tree *S* must display *BC*|*A* and thus $x:={{\,\mathrm{lca}\,}}_S(A,B)={{\,\mathrm{lca}\,}}_S(A,C)$. Since $ab\in E$ and $ac\notin E$ we observe $\tau _{T}({{\,\mathrm{lca}\,}}_T(a,b))<\tau _{S}(x)\le {{\,\mathrm{lca}\,}}_T(a,c)$ and, as in Case (1) we infer that *T* displays *ab*|*c*. By similar arguments, $a'c\in E$ and $ac\notin E$ implies that *T* displays $a'c|a$. By Lemma  5, $T_{|abcd} = ((a,b),(a',c))$ and thus, $y:={{\,\mathrm{lca}\,}}_T(a',b) = {{\,\mathrm{lca}\,}}_T(a,c)$ and $a'b\in E$ implies that $\tau _{T}(y)<\tau _{S}(x)$. Since $y= {{\,\mathrm{lca}\,}}_T(a,c)$ and $\tau _{T}(y)<\tau _{S}(x)=\tau _{S}({{\,\mathrm{lca}\,}}_S(A,C))$, we can conclude that $ac\in E$. Hence, *a*, *b*, *c*, *d* do not induce a $P_4$ in *G*; a contradiction.

In Case (4) the $P_4$ is of the form $a-b-a'-b'$ with $\sigma (a)=\sigma (a')=A$ and $\sigma (b)=\sigma (b')=B$. Now, $ab,a'b'\in E$ and $ab'\notin E$ imply that $\tau _{T}({{\,\mathrm{lca}\,}}_T(a,b)), \tau _{T}({{\,\mathrm{lca}\,}}_T(a',b')) < \tau _{S}({{\,\mathrm{lca}\,}}_S(A,B))\le \tau _{T}({{\,\mathrm{lca}\,}}_T(a,b'))$. Hence, by similar arguments as above, *T* must display $ab|b'$ and $a'b'|a$. By Lemma 5, $T_{abcd} = ((a,b),(a',b'))$ and thus, $y:={{\,\mathrm{lca}\,}}_T(a'b) = {{\,\mathrm{lca}\,}}_T(a,b')$. However, $a'b\notin E$ implies that $\tau _{T}(y)<\tau _{S}({{\,\mathrm{lca}\,}}_S(A,B))$; a contradiction to $\tau _{S}({{\,\mathrm{lca}\,}}_S(A,B))\le \tau _{T}({{\,\mathrm{lca}\,}}_T(a,b'))$. $\square$

The converse of Lemma 8 is not true in general. To see this, consider the properly-colored cograph $$(G,\sigma )$$ with vertex $$V(G)=\{a,a',b,b',c,c'\}$$, edges $$ab,bc, a'b',a'c' $$ and coloring $$\sigma (a)=\sigma (a')=A$$ $$\sigma (b)=\sigma (b')=B$$, $$\sigma (c)=\sigma (c')=C$$ with *A*, *B*, *C* being pairwise distinct. In this case, $${\mathfrak {S}}(G,\sigma )$$ contains the triples *AC*|*B* and *BC*|*A*. By Lemma 6, the tree *S* in every $$\mu $$-free scenario $${\mathcal {T}}=(T,S,\sigma ,\tau _{T},\tau _{S})$$ or relaxed scenario $${\mathcal {S}}=(T,S,\sigma ,\mu ,\tau _{T},\tau _{S})$$ explaining $$(G,\sigma )$$ displays *AC*|*B* and *BC*|*A*. Since no such scenario can exist, $$(G,\sigma )$$ is not an LDT graph.

### Recognition and characterization of LDT graphs

#### Definition 12

Let $(G=(L,E),\sigma )$ be a graph with coloring $\sigma :L\rightarrow M$. Let ${\mathscr {C}}$ be a partition of *M*, and ${\mathscr {C}}'$ be the set of connected components of *G*. We define the following binary relation ${\mathfrak {R}}(G, \sigma , {\mathscr {C}})$ by setting

$$\begin{aligned} (x,y)\in {\mathfrak {R}}(G, \sigma , {\mathscr {C}}) \iff&x,y\in L,\; \sigma (x), \sigma (y) \in C \text { for some } C\in {\mathscr {C}}, \text { and } \\&x,y \in C' \text { for some } C'\in {\mathscr {C}}'. \end{aligned}$$

In words, two vertices $x,y\in L$ are in relation ${\mathfrak {R}}(G, \sigma , {\mathscr {C}})$ whenever they are in the same connected component of *G* and their colors $\sigma (x), \sigma (y)$ are contained in the same set of the partition of *M*.

#### Lemma 9

Let $(G=(L,E),\sigma )$ be a graph with coloring $\sigma :L\rightarrow M$ and ${\mathscr {C}}$ be a partition of *M*. Then, ${\mathfrak {R}}:={\mathfrak {R}}(G, \sigma , {\mathscr {C}})$ is an equivalence relation and every equivalence class of ${\mathfrak {R}}$, or short ${\mathfrak {R}}$-class, is contained in some connected component of *G*. In particular, each connected component of *G* is the disjoint union of ${\mathfrak {R}}$-classes.

#### Proof

It is easy to see that ${\mathfrak {R}}$ is reflexive and symmetric. Moreover, $xy,yz\in {\mathfrak {R}}$ implies that $\sigma (x), \sigma (y), \sigma (z)$ must be contained in the same set of the partition ${\mathscr {C}}$, and *x*, *y*, *z* must be contained in the same connected component of *G*. Therefore, $xy\in {\mathfrak {R}}$ and thus, ${\mathfrak {R}}$ is transitive. In summary, ${\mathfrak {R}}$ is an equivalence relation.

We continue with showing that every ${\mathfrak {R}}$-class *K* is entirely contained in some connected component of *G*. Clearly, there is a connected component *C* of *G* such that $C\cap K\ne \emptyset$. Assume, for contradiction, that $K\not \subseteq C$. Hence, *G* must be disconnected and, in particular, there is a second connected component $C'$ of *G* such that $C'\cap K\ne \emptyset$. Hence, there is a pair $xy\in K$ such that $x\in C\cap K$ and $y\in C'\cap K$. But then *x* and *y* are in different connected components of *G* violating the definition of ${\mathfrak {R}}$; a contradiction. Hence, every ${\mathfrak {R}}$-class is entirely contained in some connected component of *G*. This and the fact the ${\mathfrak {R}}$-classes are disjoint implies that each connected component of *G* is the disjoint union of ${\mathfrak {R}}$-classes. $\square$

The following partition of the leaf sets of subtrees of a tree *S* rooted at some vertex $u\in V(S)$ will be useful:

$$\begin{aligned}&\text {If } u \text { is not a leaf, then }&{\mathscr {C}}_{S}(u)&:=\{L(S(v)) \mid v\in {{\,\mathrm{child}\,}}_S(u)\} \\&\text {and, otherwise, }&{\mathscr {C}}_{S}(u)&:=\{\{u\}\}. \end{aligned}$$

One easily verifies that, in both cases, ${\mathscr {C}}_{S}(u)$ yields a valid partition of the leaf set *L*(*S*(*u*)). Recall that $\sigma _{|L',M'}:L'\rightarrow M'$ was defined as the “submap” of $\sigma$ with $L'\subseteq L$ and $\sigma (L') \subseteq M' \subseteq M$.

#### Lemma 10

Let $(G=(L,E),\sigma )$ be a properly colored cograph. Suppose that the triple set ${\mathfrak {S}}(G,\sigma )$ is compatible and let *S* be a tree on *M* that displays ${\mathfrak {S}}(G,\sigma )$. Moreover, let $L'\subseteq L$ and $u\in V(S)$ such that $\sigma (L') \subseteq L(S(u))$. Finally, set ${\mathfrak {R}}:={\mathfrak {R}}(G[L'],\sigma _{|L',L(S(u))},{\mathscr {C}}_{S}(u))$.

Then, for all distinct ${\mathfrak {R}}$-classes *K* and $K'$, either $xy\in E$ for all $x\in K$ and $y\in K'$, or $xy\notin E$ for all $x\in K$ and $y\in K'$. In particular, for $x\in K$ and $y\in K'$, it holds that

$$\begin{aligned} xy\in E \iff K, K' \text { are contained in the same connected component of } G[L']. \end{aligned}$$

#### Proof

Let $\sigma :L\rightarrow M$ and put ${\mathfrak {S}}= {\mathfrak {S}}(G,\sigma )$. Since ${\mathfrak {S}}$ is a compatible triple set on *M*, there is a tree *S* on *M* that displays ${\mathfrak {S}}$. Moreover, the condition $\sigma (L') \subseteq L(S(u))\subseteq M$ together with the fact that ${\mathscr {C}}_{S}(u)$ is a partition of *L*(*S*(*u*)) ensures that ${\mathfrak {R}}$ is well-defined.

Now suppose that *K* and $K'$ are distinct ${\mathfrak {R}}$-classes. As a consequence of Lemma 9, we have exactly the two cases: either (i) *K* and $K'$ are contained in the same connected component *C* of $G[L']$ or (ii) $K\subseteq C$ and $K'\subseteq C'$ for distinct components *C* and $C'$ of $G[L']$.

Case (i). Assume, for contradiction, that there are two vertices $x\in K$ and $y\in K'$ with $xy\notin E$. Note that $C\subseteq L'$ and thus, *G*[*C*] is an induced subgraph of $G[L']$. By Proposition 2, both induced subgraphs $G[L']$ and *G*[*C*] are cographs. Now we can again apply Proposition 2 to conclude that $\mathrm {diam}(G[C])\le 2$. Hence, there is a vertex $z\in C$ such that $xz,zy\in E$. Since *x* and *y* are in distinct classes of ${\mathfrak {R}}$ but in the same connected component *C* of $G[L']$, $\sigma (x)$ and $\sigma (y)$ must lie in distinct sets of ${\mathscr {C}}_{S}(u)$. In particular, it must hold that $\sigma (x)\ne \sigma (y)$. The fact that $G[L']$ is properly colored together with $xz, yz \in E$ implies that $\sigma (z)\ne \sigma (x),\sigma (y)$. By definition and since $G[L']$ is an induced subgraph of *G*, we obtain that $\sigma (x)\sigma (y)|\sigma (z)\in {\mathfrak {S}}$. In particular, $\sigma (x)\sigma (y)|\sigma (z)$ is displayed by *S*. Since $\sigma (x)$ and $\sigma (y)$ lie in distinct sets of ${\mathscr {C}}_{S}(u)$, *u* must be an inner vertex, and we have $\sigma (x)\in L(S(v))$ and $\sigma (y)\in L(S(v'))$ for distinct $v, v'\in {{\,\mathrm{child}\,}}_S(u)$. In particular, it must hold that ${{\,\mathrm{lca}\,}}_S(\sigma (x),\sigma (y))=u$. Moreover, $z\in C\subseteq L'$ and $\sigma (L')\subseteq L(S(u))$ imply that $\sigma (z)\in L(S(u))$. Taken together, the latter two arguments imply that *S* cannot display the triple $\sigma (x)\sigma (y)|\sigma (z)$; a contradiction.

Case (ii). By assumption, the ${\mathfrak {R}}$-classes *K* and $K'$ are in distinct connected components of $G[L']$, which immediately implies $xy\notin E$ for all $x\in K$, $y\in K'$.

In summary, either $xy\in E$ for all $x\in K$ and $y\in K'$, or $xy\notin E$ for all $x\in K$ and $y\in K'$. Moreover, Case (i) establishes the *if*-direction and Case (ii) establishes, by means of contraposition, the *only-if*-direction of the final statement. $\square$

Lemma 10 suggests a recursive strategy to construct a relaxed scenario ${\mathcal {S}}=(T,S,\sigma ,\mu ,\tau _{T},\tau _{S})$ for a given properly-colored cograph $(G,\sigma )$, which is outlined in the main part of this paper and described more formally in Algorithm 1. We proceed by proving the correctness of Algorithm 1.

#### Theorem 2

Let $(G,\sigma )$ be a properly colored cograph, and assume that the triple set ${\mathfrak {S}}(M,G)$ is compatible. Then Algorithm 1 returns a relaxed scenario ${\mathcal {S}}=(T,S,\sigma ,\mu ,\tau _{T},\tau _{S})$ such that $G_{_{<}}({\mathcal {S}})=G$ in polynomial time.

#### Proof

Let $\sigma :L\rightarrow M$ and put ${\mathfrak {S}}:={\mathfrak {S}}(G,\sigma )$. By a slight abuse of notation, we will simply write $\mu$ and $\tau _{T}$ also for restrictions to subsets of *V*(*T*). Observe first that due to Line 1, the algorithm continues only if $(G,\sigma )$ is a properly colored cograph and ${\mathfrak {S}}$ is compatible, and returns a tuple ${\mathcal {S}}=(T,S,\sigma ,\mu ,\tau _{T},\tau _{S})$ in this case. In particular, a tree *S* on *M* that displays ${\mathfrak {S}}$ exists, and can e.g. be constructed using BUILD (Line 1). By Lemma 1, we can always construct a time map $\tau _{S}$ for *S* satisfying $\tau _{S}(x)=0$ for all $x\in L(S)$ (Line 2). By definition, $\tau _{S}(y)>\tau _{S}(x)$ must hold for every edge $(y,x)\in E(S)$, and thus, we obtain $\epsilon >0$ in Line 3. Moreover, the recursive function BuildGeneTree maintains the following invariant:

#### Claim 4

In every recursion step of the function BuildGeneTree, we have $\sigma (L')\subseteq L(S(u_S))$.

#### Proof

Since *S* (with root $\rho _S$) is a tree on *M* by construction and thus $L(S(\rho _S))=M$, the statement holds for the top-level recursion step on *L* and $\rho _S$. Now assume that the statement holds for an arbitrary step on $L'$ and $u_S$. If $u_S$ is a leaf, there are no deeper recursion steps. Thus assume that $u_S$ is an inner vertex. Recall that ${\mathscr {C}}_{S}(u_S)$ is a partition of $L(S(u_S))$ (by construction), and that ${\mathfrak {R}}= {\mathfrak {R}}(G[L'], \sigma _{|L',L(S(u))}, {\mathscr {C}}_{S}(u_S))$ is an equivalence relation (by Lemma 9). This together with the definition of ${\mathfrak {R}}$ and $\sigma (L')\subseteq L(S(u_S))$, implies that there is a child $v_S\in {{\,\mathrm{child}\,}}_S(u_{S})$ such that $\sigma (K)\subseteq L(S(v_S))$ for all ${\mathfrak {R}}$-classes *K*. In particular, therefore, the statement is true for all recursive calls on *K* and $v_S$ in Line 21. Repeating this argument top-down along the recursion hierarchy proves the claim. $\diamond$

Note, that we are in the *else*-condition in Line 13 only if $u_S$ is not a leaf. Therefore and as a consequence of Claim 4 and by similar arguments as in its proof, there is a vertex $v^*_S\in {{\,\mathrm{child}\,}}_S(u_S)$ such that $\sigma (C)\cap L(S(v^*_S))\ne \emptyset$ for every connected component *C* of $G[L']$ in Line 17, and a vertex $v_S\in {{\,\mathrm{child}\,}}_S(u_{S})$ such that $\sigma (K)\subseteq L(S(v_S))$ for every ${\mathfrak {R}}$-class *K* in Line 20. Moreover, ${{\,\mathrm{par}\,}}_S(u_{S})$ is always defined since we have $u_S=\rho _S$ and thus ${{\,\mathrm{par}\,}}_S(u_S)=0_S$ in the top-level recursion step, and recursively call the function BuildGeneTree on vertices $v_S$ such that $v_S\prec _S u_S$.

In summary, all assignments are well-defined in every recursion step. It is easy to verify that the algorithm terminates since, in each recursion step, we either have that $u_S$ is a leaf, or we recurse on vertices $v_{S}$ that lie strictly below $u_S$. We argue that the resulting tree $T'$ is a *not necessarily phylogenetic* tree on *L* by observing that, in each step, each $x\in L'$ is either attached to the tree as a leaf if $u_S$ is a leaf, or, since ${\mathfrak {R}}$ forms a partition of $L'$ by Lemma 9, passed down to a recursion step on *K* for some ${\mathfrak {R}}$-class *K*. Nevertheless, $T'$ is turned into a phylogenetic tree *T* by suppression of degree-two vertices in Line 25. Finally, $\mu (x)$ and $\tau _{T}(x)$ are assigned for all vertices $x\in L(T')=L$ in Line 11, and for all newly created inner vertices in Lines 7 and 18.

Recall that $\tau _{S}$ is a valid time map satisfying $\tau _{S}(x)=0$ for all $x\in L(S)$ by construction. Before we continue to show that ${\mathcal {S}}$ is a relaxed scenario, we first show that the conditions for time maps and time consistency are satisfied for $(T',\tau _{T}, S, \tau _{S},\mu )$:

#### Claim 5

For all $x,y \in V(T')$ with $x\prec _{T'} y$, we have $\tau _{T}(x)<\tau _{T}(y)$. Moreover, for all $x\in V(T')$, the following statements are true:

(i) if $\mu (x)\in V(S)$, then $\tau _{T}(x)=\tau _{S}(\mu (x))$, and
(ii) if $\mu (x)=(a,b)\in E(S)$, then $\tau _{S}(b)<\tau _{T}(x)<\tau _{S}(a)$.

#### Proof

Recall that we always write an edge (*u*, *v*) of a tree *T* such that $v\prec _T u$. For the first part of the statement, it suffices to show that $\tau _{T}(x)<\tau _{T}(y)$ holds for every edge $(y,x)\in E(T')$, and thus to consider all vertices $x\ne \rho _{T'}$ in $T'$ and their unique parent, which will be denoted by *y* in the following. Likewise, we have to consider all vertices $x\in V(T')$ including the root to show the second statement. The root $\rho _{T'}$ of $T'$ corresponds to the vertex $u_T$ created in Line 6 in the top-level recursion step on *L* and $\rho _{S}$. Hence, we have $\mu (\rho _{T'})=({{\,\mathrm{par}\,}}_S(\rho _S)=0_S,\rho _S)\in E(S)$ and $\tau _{T}(\rho _{T'})=\tau _{S}(\rho _S) +\epsilon$ (cf. Line 7). Therefore, we have to show (ii). Since $\epsilon >0$, it holds that $\tau _{S}(\rho _S)<\tau _{T}(\rho _{T'})$. Moreover, $\tau _{S}(0_S)-\tau _{S}(\rho _{S})\ge 3\epsilon$ holds by construction, and thus $\tau _{S}(0_S)-(\tau _{T}(\rho _{T'})-\epsilon )\ge 3\epsilon$ and $\tau _{S}(0_S)-\tau _{T}(\rho _{T'})\ge 2\epsilon$, which together with $\epsilon >0$ implies $\tau _{T}(\rho _{T'})<\tau _{S}(0_S)$.

We now consider the remaining vertices $x\in V(T'){\setminus }\{\rho _{T'}\}$. Every such vertex *x* is introduced into $T'$ in some recursion step on $L'$ and $u_S$ in one of the Lines 6, 10,  15 or 21. There are exactly the following three cases: (a) $x\in L(T')$ is a leaf attached to some inner vertex $u_T$ in Line 10, (b) $x=v_T$ as created in Line 15, and (c) $x=w_T$ as assigned in Line 21. Note that if $x=u_T$ as created in Line 6, then $u_T$ is either the root of $T'$, or equals a vertex $w_T$ as assigned in Line 21 in the “parental” recursion step.

In Case (a), we have that $x\in L(T')$ is a leaf and attached to some inner vertex $y=u_T$. Since $u_S$ must be a leaf in this case, and thus $\tau _{S}(u_S)=0$, we have $\tau _{T}(y)=0+\epsilon =\epsilon$ and $\tau _{T}(x)=0$ (cf. Lines 7 and 11). Since $\epsilon >0$, this implies $\tau _{T}(x)<\tau _{T}(y)$. Moreover, we have $\mu (x)=\sigma (x)\in L(S)\subset V(S)$ (cf. Line 11), and thus have to show Subcase (i). Since $u_S$ is a leaf and $\sigma (L')\subseteq L(S(u_S))$, we conclude $\sigma (x)=u_S$. Thus we obtain $\tau _{T}(x)=0=\tau _{S}(u_S)=\tau _{S}(\mu (x))$.

In Case (b), we have $x=v_T$ as created in Line 15, and *x* is attached as a child to some vertex $y=u_T$ created in the same recursion step. Thus, we have $\tau _{T}(y)=\tau _{S}(u_S)+\epsilon$ and $\tau _{T}(x)=\tau _{S}(u_S)-\epsilon$ (cf. Lines 7 and 18). Therefore and since $\epsilon >0$, it holds $\tau _{T}(x)<\tau _{T}(y)$. Moreover, we have $\mu (x)=(u_S,v^*_S)\in E(S)$ for some $v^*_S\in {{\,\mathrm{child}\,}}_S(u_S)$. Hence, we have to show Subcase (ii). By a similar calculation as before, $\epsilon >0$, $\tau _{S}(u_S)-\tau _{S}(v^*_S)\ge 3\epsilon$ and $\tau _{T}(x)=\tau _{S}(u_S)-\epsilon$ imply $\tau _{S}(v^*_S)<\tau _{T}(x)<\tau _{S}(u_S)$.

In Case (c), $x=w_T$ as assigned in Line 21 is equal to $u_T$ as created in Line 6 in some next-deeper recursion step with $u'_S\in {{\,\mathrm{child}\,}}_S(u_S)$. Thus, we have $\tau _{T}(x)=\tau _{S}(u'_S)+\epsilon$ and $\mu (x)=(u_S,u'_S)\in E(S)$ (cf. Line 7). Moreover, *x* is attached as a child of some vertex $y=v_T$ as created in Line 15. Thus, we have $\tau _{T}(y)=\tau _{S}(u_S)-\epsilon$. By construction and since $(u_S,u'_S)\in E(S)$, we have $\tau _{S}(u_S)-\tau _{S}(u'_S)\ge 3\epsilon$. Therefore, $(\tau _{T}(y)+\epsilon ) - (\tau _{T}(x)-\epsilon ) \ge 3\epsilon$ and thus $\tau _{T}(y)- \tau _{T}(x) \ge \epsilon$. This together with $\epsilon >0$ implies $\tau _{T}(x)<\tau _{T}(y)$. Moreover, since $\mu (x)=(u_S,u'_S)\in E(S)$ for some $u'_S\in {{\,\mathrm{child}\,}}_S(u_S)$, we have to show Subcase (ii). By a similar calculation as before, $\epsilon >0$, $\tau _{S}(u_S)-\tau _{S}(u'_S)\ge 3\epsilon$ and $\tau _{T}(x)=\tau _{S}(u'_S)+\epsilon$ imply $\tau _{S}(u'_S)<\tau _{T}(x)<\tau _{S}(u_S)$. $\diamond$

#### Claim 6

${\mathcal {S}}=(T,S,\sigma ,\mu ,\tau _{T},\tau _{S})$ is a relaxed scenario.

#### Proof

The tree *T* is obtained from $T'$ by first adding a planted root $0_T$ (and connecting it to the original root) and then suppressing all inner vertices except $0_T$ that have only a single child in Line  25. In particular, *T* is a planted phylogenetic tree by construction. The root constraint (G0) $\mu (x)=0_S$ if and only if $x=0_T$ also holds by construction (cf. Line 26). Since we clearly have not contracted any outer edges (*y*, *x*), i.e. with $x\in L(T')$, we conclude that $L(T')=L(T)=L$. As argued before, we have $\tau _{T}(x)=0$ and $\mu (x)=\sigma (x)$ whenever $x\in L(T')=L(T)$ (cf. Line 11). Since all other vertices are either $0_T$ or mapped by $\mu$ to some edge of *S* (cf. Lines 26, 7 and 18), the leaf constraint (G1) $\mu (x)=\sigma (x)$ is satisfied if and only if $x\in L(T)$.

By construction, we have $V(T){\setminus } \{0_T\} \subseteq V(T')$. Moreover, suppression of vertices clearly preserves the $\preceq$-relation between all vertices $x,y\in V(T){\setminus } \{0_T\}$. Together with Claim 5, this implies $\tau _{T}(x)<\tau _{T}(y)$ for all vertices $x,y\in V(T){\setminus } \{0_T\}$ with $x\prec _{T} y$. For the single child $\rho _T$ of $0_T$ in *T*, we have $\tau _{T}(\rho _T)\le \tau _{S}(\rho _S)+\epsilon$ where equality holds if the root of $T'$ was not suppressed and thus is equal to $\rho _T$. Moreover, $\tau _{T}(0_T)=\tau _{S}(0_S)$ and $\tau _{S}(0_S)-\tau _{S}(\rho _S)\ge 3\epsilon$ hold by construction. Taken together the latter two arguments imply that $\tau _{T}(\rho _T)<\tau _{T}(0_T)$. In particular, we obtain $\tau _{T}(x)<\tau _{T}(y)$ for all vertices $x,y\in V(T)$ with $x\prec _{T} y$. Hence, $\tau _{T}$ is a time map for *T*, which, moreover, satisfies $\tau _{T}(x)=0$ for all $x\in L(T)$.

To show that ${\mathcal {S}}=(T,S,\sigma ,\mu , \tau _{T},\tau _{S})$ is a relaxed scenario, it remains to show that $\mu$ is time-consistent with the time maps $\tau _{T}$ and $\tau _{S}$. In case $x\in L(T)\subset V(T)$, we have $\mu (x)=\sigma (x)\in L(S)\subset V(S)$ and thus $\tau _{T}(x)=0=\tau _{S}(\sigma (x))=\tau _{S}(\mu (x))$. For $0_T$, we have $\tau _{T}(0_T)=\tau _{S}(0_S)=\tau _{S}(\mu (0_T))$. The latter two arguments imply that all vertices $x\in L(T)\cup \{0_T\}$ satisfy (C1) in the Definition 4. The remaining vertices of *T* are all vertices of $T'$ as well. In particular, they are all inner vertices that are mapped to some edge of *S* (cf. Lines 7 and 18). The latter two arguments together with Claim 5 imply that, for all vertices $x\in V(T){\setminus } (L(T)\cup \{0_T\})$, we have $\mu (x)=(a,b)\in E(S)$ and $\tau _{S}(b)<\tau _{T}(x)<\tau _{S}(a)$. Therefore, every such vertex satisfies (C2) in Definition 4. It follows that the time consistency constraint (G2) is also satisfied, and thus ${\mathcal {S}}$ is a relaxed scenario. $\diamond$

#### Claim 7

Every vertex $v\in V^0(T)$ was either created in Line 6 or in Line 15. In particular, it holds for all $x,y\in L(T)$ with ${{\,\mathrm{lca}\,}}_T(x,y)=v$:

1. If *v* was created in Line 6, then $xy\notin E(G)$ and $xy\notin E(G_{_{<}}({\mathcal {S}}))$.
2. If *v* was created in Line 15, then $xy\in E(G)$ and $xy\in E(G_{_{<}}({\mathcal {S}}))$.

Furthermore, *G* is a cograph with cotree (*T*, *t*) where $t(v) = 0$ if *v* was created in Line 6 and $t(v) = 1$, otherwise.

#### Proof

Since *T* is phylogenetic, every vertex $v\in V^0(T)$ is the last common ancestor of two leaves $x,y\in L:=L(T)$. Let $v\in V^0(T)$ be arbitrary and choose arbitrary leaves $x,y\in L$ such that ${{\,\mathrm{lca}\,}}_T(x,y)=v$. Since $v\in V^0(T)$, the leaves *x* and *y* must be distinct.

Note that $v\notin L(T)\cup \{0_T\}$, and thus, *v* is also an inner vertex in $T'$. Therefore, we have exactly the two cases (1) $v=u_T$ is created in Line 6, and (2) $v=v_T$ is created in Line 15. Similar as before, the case that $v=w_K$ is assigned in Line 21 is covered by Case (a), since, in this case, $w_K$ is created in a deeper recursion step.

We consider the recursion step on $L'$ and $u_S$, in which *v* was created. Clearly, it must hold that $x,y\in L'$. Before we continue, set ${\mathfrak {R}}:={\mathfrak {R}}(G[L'], \sigma _{|L',L(S(u))}, {\mathscr {C}}_{S}(u_S))$ as in Line 13. Note, since ${\mathcal {S}}$ is a relaxed scenario, the graph $(G_{_{<}}({\mathcal {S}}),\sigma )$ is well-defined.

For Statement (1), suppose that $v=u_T$ was created in Line 6. Hence, we have the two cases (i) the vertex $u_S$ of *S* in this recursion step is a leaf, and (ii) $u_S$ is an inner vertex. In Case (i), we have $L(S(u_S))=\{u_S\}$. Together with Claim 4 and $\sigma (x),\sigma (y)\in \sigma (L')$, this implies $\sigma (x)=\sigma (x)=u_S$. By assumption, $(G,\sigma )$ is properly colored. By Proposition 3$(G_{_{<}}({\mathcal {S}}),\sigma )$ must be properly colored as well. Hence, we conclude that $xy\notin E(G)$ and $xy\notin E(G_{_{<}}({\mathcal {S}}))$, respectively. In Case (ii), $u_S$ is not a leaf. Therefore, ${{\,\mathrm{lca}\,}}_{T}(x,y)=v=u_T$ is only possible if *x* and *y* lie in distinct connected components of $G[L']$. This immediately implies $xy\notin E(G)$. Moreover, we have $\sigma (x),\sigma (y)\in L(S(u_S))$ and thus ${{\,\mathrm{lca}\,}}_S(\sigma (x),\sigma (y))\preceq _{S} u_S$. Since $\tau _{S}$ is a time map for *S*, it follows that $\tau _{S}({{\,\mathrm{lca}\,}}_S(\sigma (x),\sigma (y)))\le \tau _{S}(u_S)$. Together with $\tau _{T}(u_T)=\tau _{S}(u_S)+\epsilon$ (cf. Line 7) and $\epsilon >0$, this implies $\tau _{S}({{\,\mathrm{lca}\,}}_S(\sigma (x),\sigma (y))) < \tau _{T}(v)=\tau _{T}({{\,\mathrm{lca}\,}}_T(x,y))$. Hence, $xy\notin E(G_{_{<}}({\mathcal {S}}))$.

For Statement (2), suppose that $v=v_T$ was created in Line 15. Therefore, ${{\,\mathrm{lca}\,}}_{T}(x,y)=v=v_T$ is only possible if *x* and *y* lie in the same connected components of $G[L']$ but in distinct ${\mathfrak {R}}$-classes. Now, we can apply Lemma 10 to conclude that $xy\in E(G)$. Moreover, the fact that *x* and *y* lie in the same connected component of $G[L']$ but in distinct ${\mathfrak {R}}$-classes implies that $\sigma (x)$ and $\sigma (y)$ lie in distinct sets of ${\mathscr {C}}_{S}(u_S)$. Hence, there are distinct $v_S,v'_S\in {{\,\mathrm{child}\,}}_S(u)$ such that $\sigma (x)\preceq _{S}v_S$ and $\sigma (y)\preceq _{S} v'_S$. In particular, ${{\,\mathrm{lca}\,}}_S(\sigma (x),\sigma (y))=u_S$. In Line 18, we assign $\tau _{T}({{\,\mathrm{lca}\,}}_T(x,y))=\tau _{T}(v_T)=\tau _{S}(u_S)-\epsilon$. Together with $\epsilon >0$, the latter two arguments imply $\tau _{T}({{\,\mathrm{lca}\,}}_T(x,y))<\tau _{S}(u_S)=\tau _{S}({{\,\mathrm{lca}\,}}_S(\sigma (x),\sigma (y)))$. Therefore, we have $xy\in E(G_{_{<}}({\mathcal {S}}))$.

By the latter arguments, the cotree (*T*, *t*) as defined above is well-defined and, for all $v\in V^0(T)$, we have $t(v)=1$ if and only if $xy\in E(G)$ for all $x,y\in L$ with ${{\,\mathrm{lca}\,}}_T(x,y)=v$. Hence, (*T*, *t*) is a cotree for *G*. $\diamond$

#### Claim 8

The relaxed scenario ${\mathcal {S}}$ satisfies $G_{_{<}}({\mathcal {S}})=G$.

#### Proof

Since $L(T)=L$, the two undirected graphs $G_{_{<}}({\mathcal {S}})$ and *G* have the same vertex set. By Claim 7, we have, for all distinct $x,y\in L$, either $xy\notin E(G)$ and $xy\notin E(G_{_{<}}({\mathcal {S}}))$, or $xy\in E(G)$ and $xy\in E(G_{_{<}}({\mathcal {S}}))$. $\diamond$

Together, Claims 6 and 8 imply that Algorithm 1 returns a relaxed scenario ${\mathcal {S}}=(T,S,\sigma ,\mu ,\tau _{T},\tau _{S})$ with coloring $\sigma$ such that $G_{_{<}}({\mathcal {S}})=G$.

To see that Algorithm 1 runs in polynomial time, we first note that the function $\texttt {BuildGeneTree()}$ operates in polynomial time. This is clear for the setup and the $\mathbf{if}$ part. The construction of ${\mathfrak {R}}$ in the $\mathbf{else}$ part involves the computation of connected components and the evaluation of Definition 12, both of which can be achieved in polynomial time. This is also true for the comparisons of color classes required to identify $v_S^*$ and $v_S$. Since the sets *K* in recursive calls of $\texttt {BuildGeneTree()}$ form a partition of $L'$, and the $v_S$ are children of $u_S$ in *S* and the depth of the recursion is bounded by *O*(|*L*(*S*)|), the total effort remains polynomial. $\square$

#### Theorem 3

A graph $(G,\sigma )$ is an LDT graph if and only if it is a properly colored cograph and ${\mathfrak {S}}(G,\sigma )$ is compatible.

#### Proof

By Lemma 6 and 8, if $(G,\sigma )$ is an LDT graph then it is a properly colored cograph and ${\mathfrak {S}}(G,\sigma )$ is compatible. Now suppose that $(G,\sigma )$ is a properly colored cograph and ${\mathfrak {S}}(G,\sigma )$ is compatible. Then, by Theorem 2, Algorithm 1 outputs a relaxed scenario ${\mathcal {S}}=(T,S,\sigma ,\mu ,\tau _{T},\tau _{S})$ such that $G_{_{<}}({\mathcal {S}})=G$. By definition, this in particular implies that $(G,\sigma )$ is an LDT graph. $\square$

#### Corollary 2

LDT graphs can be recognized in polynomial time.

#### Proof

Cographs can be recognized in linear time (Corneil et al. 1981b), the proper coloring can be verified in linear time, the triple set ${\mathfrak {S}}(G,\sigma )$ contains not more than $|V(G)|\cdot |E(G)|$ triples and can be constructed in $O(|V(G)|\cdot |E(G)|)$ time, and compatibility of ${\mathfrak {S}}(G,\sigma )$ can be checked in $O(\min (|{\mathfrak {S}}|\log ^2 |V(G)|, |{\mathfrak {S}}| + |V(G)|^2\ln |V(G)|))$ time (Jansson et al. 2005). $\square$

#### Corollary 3

The property of being an LDT graph is hereditary, that is, if $(G,\sigma )$ is an LDT graph then each of its vertex induced subgraphs is an LDT graph.

#### Proof

Let $(G=(V,E),\sigma )$ be an LDT graph. It suffices to show that $(G-x, \sigma _{|V{\setminus } \{x\}})$ is an LDT graph, where $G-x$ is obtained from *G* by removing $x\in V$ and all its incident edges. By Proposition 2, $G-x$ is a cograph that clearly remains properly colored. Moreover, every induced path on three vertices in $G-x$ is also an induced path on three vertices in *G*. This implies that if $xy|z \in {\mathfrak {S}}' = {\mathfrak {S}}(G-x,\sigma _{|V{\setminus } \{x\}})$, then $xy|z \in {\mathfrak {S}}(G,\sigma )$. Hence, ${\mathfrak {S}}' \subseteq {\mathfrak {S}}(G,\sigma )$. By Theorem 3, ${\mathfrak {S}}(G,\sigma )$ is compatible. Hence, any tree that displays all triples in ${\mathfrak {S}}(G,\sigma )$, in particular, displays all triples in ${\mathfrak {S}}'$. Therefore, ${\mathfrak {S}}'$ is compatible. In summary, $(G-x, \sigma _{|V{\setminus } \{x\}})$ is a properly colored cograph and ${\mathfrak {S}}'$ is compatible. By Theorem 3 it is an LDT graph. $\square$

The relaxed scenarios ${\mathcal {S}}$ explaining an LDT graph $(G,\sigma )$ are far from being unique. In fact, we can choose from a large set of trees $(S,\tau _{S})$ that is determined only by the triple set ${\mathfrak {S}}(G,\sigma )$:

#### Corollary 4

If $(G=(L,E),\sigma )$ is an LDT graph with coloring $\sigma :L\rightarrow M$, then for all planted trees *S* on *M* that display ${\mathfrak {S}}(G,\sigma )$ there is a relaxed scenario ${\mathcal {S}}=(T,S,\sigma ,\mu ,\tau _{T},\tau _{S})$ that contains $\sigma$ and *S* and that explains $(G,\sigma )$.

#### Proof

If $(G,\sigma )$ is an LDT graph, then the species tree *S* assigned in Line 1 in Algorithm 1 is an arbitrary tree on *M* displaying ${\mathfrak {S}}(G,\sigma )$. $\square$

#### Corollary 5

If $(G,\sigma )$ is an LDT graph, then there exists a relaxed scenario ${\mathcal {S}}=(T,S,\sigma ,\mu ,\tau _{T},\tau _{S})$ explaining $(G,\sigma )$ such that *T* displays the discriminating cotree $T_{G}$ of *G*.

#### Proof

Suppose that $(G,\sigma )$ is an LDT graph. By Theorem 3, $(G,\sigma )$ must be a properly colored cograph and ${\mathfrak {S}}(G,\sigma )$ is comparable. Hence, Theorem 2 implies that Algorithm 1 constructs a relaxed scenario ${\mathcal {S}}=(T,S,\sigma ,\mu ,\tau _{T},\tau _{S})$ explaining $(G,\sigma )$. In particular, the tree *T* together with labeling *t* as specified in Claim 7 is a cotree for *G*. Since the unique discriminating cotree $(T_{G},{{\hat{t}}})$ of *G* is obtained from any other cotree by contraction of edges in *T*, the tree *T* must display $T_{G}$. $\square$

Although, Corollary 5 implies that there is always a relaxed scenario ${\mathcal {S}}$ where the tree *T* displays the discriminating cotree $T_{G}$ of $G=G({\mathcal {S}})$, this is not true for all relaxed scenarios ${\mathcal {S}}$ with $G=G({\mathcal {S}})$. Figure 16 shows a relaxed scenario ${\mathcal {S}}' = (T',S',\sigma ,\mu ',\tau _{T}',\tau _{S}')$ with $G = G({\mathcal {S}}')$ for which $T'$ does not display $T_G$.

Corollary 5 enables us to relate connectedness of LDT graphs to properties of the relaxed scenarios by which it can be explained.

#### Lemma 11

An LDT graph $(G=(L,E),\sigma )$ with $|L|>1$ is connected if and only if for every relaxed scenario ${\mathcal {S}}=(T,S,\sigma ,\mu ,\tau _{T},\tau _{S})$ that explains $(G,\sigma )$, we have $\tau _{T}(\rho _T)<\tau _{S}({{\,\mathrm{lca}\,}}_S(\sigma (L)))$.

#### Proof

By contraposition, suppose first that there is a relaxed scenario ${\mathcal {S}}=(T,S,\sigma ,\mu ,\tau _{T},\tau _{S})$ that explains $(G,\sigma )$ such that $\tau _{T}(\rho _T) \ge \tau _{S}({{\,\mathrm{lca}\,}}_S(\sigma (L)))$. Since $|L(T)|=|L|>1$, the root $\rho _{T}$ is not a leaf. To show that *G* is disconnected we consider two distinct children $v,w\in {{\,\mathrm{child}\,}}(\rho _T)$ of the root and leaves $x\in L(T(v))$ and $y\in L(T(w))$ and verify that *x* and *y* cannot be adjacent in *G*. If $\sigma (x)=\sigma (y)$, then $xy\notin E$ since $(G,\sigma )$ is properly colored (cf. Lemma 8). Hence, suppose that $\sigma (x)\ne \sigma (y)$. By construction, ${{\,\mathrm{lca}\,}}_T(x,y)=\rho _T$ and thus, by assumption, $\tau _{T}({{\,\mathrm{lca}\,}}_T(x,y)) = \tau _{T}(\rho _T) \ge \tau _{S}({{\,\mathrm{lca}\,}}_S(\sigma (L)))$. Now ${{\,\mathrm{lca}\,}}_S(\sigma (L))\succeq _S {{\,\mathrm{lca}\,}}_S(\sigma (x),\sigma (y))$ implies that $\tau _{S}({{\,\mathrm{lca}\,}}_S(\sigma (L)))\ge \tau _{S}({{\,\mathrm{lca}\,}}_S(\sigma (x),\sigma (y)))$ and thus, $\tau _{T}({{\,\mathrm{lca}\,}}_T(x,y))\ge \tau _{S}({{\,\mathrm{lca}\,}}_S(\sigma (x),\sigma (y)))$. Hence, $xy\notin E$. Consequently, for all distinct children $v,w\in {{\,\mathrm{child}\,}}(\rho _T)$, none of the vertices in *L*(*T*(*v*)) are adjacent to any of the vertices in *L*(*T*(*w*)) and thus, *G* is disconnected.

Conversely, suppose that *G* is disconnected. We consider Algorithm 1 with input $(G,\sigma )$. By Theorems 2 and 3, the algorithm constructs a relaxed scenario ${\mathcal {S}}=(T,S,\sigma ,\mu ,\tau _{T},\tau _{S})$ that explains $(G,\sigma )$. Consider the top-level recursion step on *L* and $\rho _S$. Since *G* is disconnected, the vertex $u_T$ created in Line 6 of this step equals the root $\rho _T$ of the final tree *T*. To see this, assume first that $\rho _S$ is a leaf. Then, we attach the $|L|>1$ elements in *L* as leaves to $u_T$ (cf. Line 10). Now assume that $\rho _S$ is not a leaf. Since $G[L]=G$ has at least two components, we attach at least two vertices $v_T$ created in Line 15 to $u_T$. Hence $u_T$ is not suppressed in Line 25 and thus $\rho _T=u_T$. By construction, therefore, we have $\tau _{T}(\rho _T)=\tau _{T}(u_T)=\tau _{S}(u_S)+\epsilon =\tau _{S}(\rho _S)+\epsilon$ for some $\epsilon >0$. From $\sigma (\rho _S)\succeq _S {{\,\mathrm{lca}\,}}_S(\sigma (L))$ and the definition of time maps, we obtain $\tau _{S}(\rho _S)\ge \tau _{S}({{\,\mathrm{lca}\,}}_S(\sigma (L)))$. Therefore, we have $\tau _{T}(\rho _T)\ge \tau _{S}({{\,\mathrm{lca}\,}}_S(\sigma (L)))+\epsilon >\tau _{S}({{\,\mathrm{lca}\,}}_S(\sigma (L)))$, which completes the proof. Therefore, we have shown so-far that if all relaxed scenarios ${\mathcal {S}}=(T,S,\sigma ,\mu ,\tau _{T},\tau _{S})$ that explain $(G,\sigma )$ satisfy $\tau _{T}(\rho _T)\le \tau _{S}({{\,\mathrm{lca}\,}}_S(\sigma (L)))$, then $(G,\sigma )$ must be connected. However, $\tau _{T}(\rho _T) = \tau _{S}({{\,\mathrm{lca}\,}}_S(\sigma (L)))$ cannot occur, since we can reuse the same arguments as in the beginning of this proof to show that, in this case, *G* is disconnected.

$$\square $$

### Least resolved trees for LDT graphs

As we have seen e.g. in Corollary 4, there are in general many trees *S* and *T* forming relaxed scenarios ${\mathcal {S}}$ that explain a given LDT graph $(G,\sigma )$. This begs the question to what extent these trees are determined by “representatives”. For *S*, we have seen that *S* always displays ${\mathfrak {S}}(G,\sigma )$, suggesting to consider the role of $S={{\,\mathrm{Aho}\,}}({\mathfrak {S}}(G,\sigma ), M)$. This tree is least resolved in the sense that there is no relaxed scenario explaining the LDT graph $(G,\sigma )$ with a tree $S'$ that is obtained from *S* by edge-contractions. The latter is due to the fact that any edge contraction in ${{\,\mathrm{Aho}\,}}({\mathfrak {S}}(G,\sigma ), M)$ yields a tree $S'$ that does not display ${\mathfrak {S}}(G,\sigma )$ any more (Jansson et al. 2012). By Proposition 6, none of the relaxed scenarios containing $S'$ explain the LDT $(G,\sigma )$.

#### Definition 13

Let ${\mathcal {S}}=(T,S,\sigma ,\mu ,\tau _{T},\tau _{S})$ be a relaxed scenario explaining the LDT graph $(G,\sigma )$. The planted tree *T* is *least resolved* for $(G,\sigma )$ if no relaxed scenario $(T',S',\sigma ',\mu ',\tau _{T}',\tau _{S}')$ with $T'<T$ explain $(G,\sigma )$.

In other words, *T* is least resolved for $(G,\sigma )$ if no scenario with a gene tree $T'$ obtained from *T* by a series of edge contractions explains $(G,\sigma )$. The examples in Fig. 3 show that there is not always a unique least resolved tree.

As outlined in the main part of this paper, the examples in Fig. 3 show that LDT graphs are in general not accompanied by unique least resolved trees and the example in Fig. 4 shows that the unique discriminating cotree $T_G$ of an LDT graph $(G,\sigma )$ is not always “sufficiently resolved”.

## Horizontal gene transfer and Fitch graphs

### HGT-labeled trees and rs-Fitch graphs

As alluded to in the introduction, the LDT graphs are intimately related with horizontal gene transfer. To formalize this connection we first define transfer edges. These will then be used to encode Walter Fitch’s concept of xenologous gene pairs (Fitch 2000; Darby et al. 2017) as a binary relation, and thus, the edge set of a graph.

#### Definition 14

Let ${\mathcal {S}}= (T,S,\sigma ,\mu ,\tau _{T},\tau _{S})$ be a relaxed scenario. An edge (*u*, *v*) in *T* is a *transfer edge* if $\mu (u)$ and $\mu (v)$ are incomparable in *S*. The *HGT-labeling* of *T* in ${\mathcal {S}}$ is the edge labeling $\lambda _{{\mathcal {S}}}: E(T)\rightarrow \{0,1\}$ with $\lambda (e)=1$ if and only if *e* is a transfer edge.

The vertex *u* in *T* thus corresponds to an HGT event, with *v* denoting the subsequent event, which now takes place in the “recipient” branch of the species tree. Note that $\lambda _{{\mathcal {S}}}$ is completely determined by ${\mathcal {S}}$. In general, for a given a gene tree *T*, HGT events correspond to a labeling or coloring of the edges of *T*.

#### Definition 15

(*Fitch graph*) Let $(T,\lambda )$ be a tree *T* together with a map $\lambda :E(T)\rightarrow \{0,1\}$. The *Fitch graph* $\digamma (T,\lambda ) = (V,E)$ has vertex set $V:=L(T)$ and edge set

$$\begin{aligned}&E :=\{xy \mid x,y\in L, \text { the unique path connecting } x\\&\quad \text { and } y \text { in } T \text { contains an edge } e \text { with } \lambda (e)=1. \} \end{aligned}$$

By definition, Fitch graphs of 0/1-edge-labeled trees are loop-less and undirected. We call edges *e* of $(T,\lambda )$ with label $\lambda (e)=1$ also 1-edges and, otherwise, 0-edges.

#### Remark 4

Fitch graphs as defined here have been termed *undirected* Fitch graphs (Hellmuth et al. 2018), in contrast to the notion of the *directed* Fitch graphs of 0/1-edge-labeled trees studied e.g. in Geiß et al. (2018) and Hellmuth and Seemann (2019).

#### Proposition 5

(Hellmuth et al. 2018; Zverovich 1999) The following statements are equivalent.

1. *G* is the Fitch graph of a 0/1-edge-labeled tree.
2. *G* is a complete multipartite graph.
3. *G* does not contain $K_2+K_1$ as an induced subgraph.

A natural connection between LDT graphs and complete multipartite graphs is suggested by the definition of triple sets ${\mathfrak {T}}(G)$, since each forbidden induced subgraph $K_2+K_1$ of a complete multipartite graphs corresponds to a triple in an LDT graph. More precisely, we have:

#### Lemma 12

$(G,\sigma )$ is a properly colored complete multipartite if and only if it is properly colored and ${\mathfrak {T}}(G) = \emptyset$.

#### Proof

The equivalence between the statements can be seen by observing that *G* is a complete multipartite graph if and only if *G* does not contain an induced $K_2+K_1$ (cf. Proposition 5). By definition of ${\mathfrak {T}}(G)$, this is the case if and only if ${\mathfrak {T}}(G)=\emptyset$. $\square$

#### Definition 16

(*rs-Fitch graph*) Let ${\mathcal {S}}= (T,S,\sigma ,\mu ,\tau _{T},\tau _{S})$ be a relaxed scenario with HGT-labeling $\lambda _{{\mathcal {S}}}$. We call the vertex colored graph $(\digamma ({\mathcal {S}}),\sigma ) :=(\digamma (T,\lambda _{{\mathcal {S}}}),\sigma )$ the *Fitch graph of the scenario* ${\mathcal {S}}$.

A vertex colored graph $(G,\sigma )$ is a *relaxed scenario Fitch graph* (*rs-Fitch graph*) if there is a relaxed scenario ${\mathcal {S}}= (T,S,\sigma ,\mu ,\tau _{T},\tau _{S})$ such that $G = \digamma ({\mathcal {S}})$.

Figure 5 shows that rs-Fitch graphs are not necessarily properly colored. A subtle difficulty arises from the fact that Fitch graphs of 0/1-edge-labeled trees are defined without a reference to the vertex coloring $\sigma$, while the rs-Fitch graph is vertex colored.

#### Observation 1

If $(G,\sigma )$ is an rs-Fitch graph then *G* is a complete multipartite graph.

The “converse” of Observation 1 is not true in general, as we shall see in Theorem 6 below. If, however, the coloring $\sigma$ can be chosen arbitrarily, then every complete multipartite graph *G* can be turned into an rs-Fitch graph $(G,\sigma )$ as shown in Proposition 6.

#### Proposition 6

If *G* is a complete multipartite graph, then there exists a relaxed scenario ${\mathcal {S}}=(T,S,\sigma ,\mu ,\tau _{T},\tau _{S})$ such that $(G,\sigma )$ is an rs-Fitch graph.

#### Proof

Let *G* be a complete multipartite graph and set $L:=V(G)$ and $R:=E(G)$. If $R=\emptyset$, then the relaxed scenario ${\mathcal {S}}$ constructed in the proof of Lemma 4 shows that $E(G)=E(\digamma ({\mathcal {S}})) = \emptyset$. Hence, we assume that $R\ne \emptyset$ and explicitly construct a relaxed scenario ${\mathcal {S}}= (T,S,\sigma ,\mu ,\tau _{T},\tau _{S})$ such that $(G,\sigma )$ is an rs-Fitch graph.

We start by specifying the coloring $\sigma :L\rightarrow M$. Since *G* is a complete multipartite graph it is determined by its independent sets $I_1,\dots ,I_k$, which form a partition of *L*. We set $M:=\{1,2,\ldots ,k\}$ and color every $x\in I_j$ with color $\sigma (x)=j$, $1\le j\le k$. By construction, $(G,\sigma )$ is properly colored, and $\sigma (x)=\sigma (y)$ whenever $xy\notin R$, i.e., whenever *x* and *y* lie in the same independent set. Therefore, we have ${\mathfrak {S}}(G,\sigma ) = \emptyset$. Let *S* be the planted star tree with leaf set $L(S)=\{1,\dots ,k\} = M$ and ${{\,\mathrm{child}\,}}_S(\rho _S)=M$. Since $R\ne \emptyset$, we have $k\ge 2$, and thus, $\rho _S$ has at least two children and is, therefore, phylogenetic. We choose the time map $\tau _{S}$ by putting $\tau _{S}(0_S)=2$, $\tau _{S}(\rho _S)=1$ and $\tau _{S}(x)=0$ for all $x\in L(S)$.

Finally, we construct the planted phylogenetic tree *T* with planted root $0_T$ and root $\rho _T$ as follows: Vertex $\rho _T$ has *k* children $u_1,\dots , u_k$. If $I_j=\{x_j\}$ consists of a single element, then we put $u_j:=x_j$ as a leaf or *T*, and otherwise, vertex $u_j$ has exactly $|I_j|$ children where ${{\,\mathrm{child}\,}}(u_j)=I_j$. Now label, for all $i\in \{2,\dots , k\}$, the edge $(\rho _T,u_i)$ with “1”, and all other edges with “0”. Since $k\ge 2$, the tree *T* is also phylogenetic by construction.

We specify the time map $\tau _{T}$ and the reconciliation map $\mu$ by defining, for every $v\in V(T)$,

$$\begin{aligned} \tau _{T}(v) :={\left\{ \begin{array}{ll} 2=\tau _{S}(0_S) \\ 0 \\ 1/2 \\ 1/4 \end{array}\right. } \mu (v) :={\left\{ \begin{array}{ll} 0_S &{}\text {if } v=0_T,\\ \sigma (v) &{}\text {if } v\in L(T),\\ (\rho _S,1) &{}\text {if } v = \rho _T, \text { and}\\ (\rho _S,i) &{}\text {if } v=u_i\not \in L(T), 1\le i\le k. \end{array}\right. } \end{aligned}$$

With the help of Fig. 17, it is now easy to verify that (i) $\tau _{T}$ is a time map for *T*, (ii) the reconciliation map $\mu$ is time-consistent, and (iii) $\lambda _{{\mathcal {S}}} = \lambda$. In summary, ${\mathcal {S}}= (T,S,\sigma ,\mu ,\tau _{T},\tau _{S})$ is a relaxed scenario, and $(G,\sigma ) = (\digamma ({\mathcal {S}}),\sigma )$ is an rs-Fitch graph. $\square$

Although every complete multipartite graph can be colored in such a way that it becomes an rs-Fitch graph (cf. Proposition 6), there are colored, complete multipartite graphs $(G,\sigma )$ that are not rs-Fitch graphs, i.e., that do not derive from a relaxed scenario (cf. Theorem 6). We summarize this discussion in the following

#### Observation 2

There are (planted) 0/1-edge labeled trees $(T,\lambda )$ and colorings $\sigma :L(T)\rightarrow M$ such that there is no relaxed scenario ${\mathcal {S}}= (T,S,\sigma ,\mu ,\tau _{T},\tau _{S})$ with $\lambda =\lambda _{{\mathcal {S}}}$.

A subtle—but important—observation is that trees $(T,\lambda )$ with coloring $\sigma$ for which Observation 2 applies may still encode an rs-Fitch graph $(\digamma (T,\lambda ),\sigma )$, see Example 1 and Fig. 6. The latter is due to the fact that $\digamma (T,\lambda ) = \digamma (T',\lambda ')$ may be possible for a different tree $(T',\lambda ')$ for which there is a relaxed scenario ${\mathcal {S}}' = (T',S,\sigma ,\mu ,\tau _{T},\tau _{S})$ with $\lambda ' = \lambda _{{\mathcal {S}}}$. In this case, $(\digamma (T,\lambda ),\sigma ) = (\digamma ({\mathcal {S}}'),\sigma )$ is an rs-Fitch graph. We shall briefly return to these issues in the discussion Sect. 8.

### LDT graphs and rs-Fitch graphs

We proceed to investigate to what extent an LDT graph provides information about an rs-Fitch graph. As we shall see in Theorem 5 there is indeed a close connection between rs-Fitch graphs and LDT graphs. We start with a useful relation between the edges of rs-Fitch graphs and the reconciliation maps $\mu$ of their scenarios.

#### Lemma 13

Let $\digamma ({\mathcal {S}})$ be an rs-Fitch graph for some relaxed scenario ${\mathcal {S}}$. Then, $ab\notin E(\digamma ({\mathcal {S}}))$ implies that ${{\,\mathrm{lca}\,}}_S(\sigma (a),\sigma (b)) \preceq _S \mu ({{\,\mathrm{lca}\,}}_T(a,b))$.

#### Proof

Assume first that $ab\notin E(\digamma ({\mathcal {S}}))$ and denote by $P_{xy}$ the unique path in *T* that connects the two vertices *x* and *y*. Clearly, $u:={{\,\mathrm{lca}\,}}_T(a,b)$ is contained in $P_{ab}$, and this path $P_{ab}$ can be subdivided into the two paths $P_{u,a}$ and $P_{u,b}$ that have only vertex *u* in common. Since $ab\notin E(\digamma ({\mathcal {S}}))$, none of the edges (*v*, *w*) along the path $P_{ab}$ in *T* is a transfer edge, and thus, the images $\mu (v)$ and $\mu (w)$ are comparable in *S*. This implies that the images of any two vertices along the path $P_{u,a}$ as well as the images of any two vertices along $P_{u,b}$ are comparable. In particular, therefore, $\mu (u)$ is comparable with both $\mu (a)=\sigma (a)=:A$ and $\mu (b)=\sigma (b)=:B$, where we may have $A=B$. Together with the fact that *A* and *B* are leaves in *S*, this implies that $\mu (u)$ is an ancestor of *A* and *B*. Since ${{\,\mathrm{lca}\,}}_S(A,B)$ is the “last” vertex that is an ancestor of both *A* and *B*, we have ${{\,\mathrm{lca}\,}}_S(A,B) \preceq _S \mu (u)$. $\square$

The next result shows that a subset of transfer edges can be inferred immediately from LDT graphs:

#### Theorem 4

If $(G,\sigma )$ is an LDT graph, then $G\subseteq \digamma ({\mathcal {S}})$ for all relaxed scenarios ${\mathcal {S}}$ that explain $(G,\sigma )$.

#### Proof

Let ${\mathcal {S}}= (T,S,\sigma ,\mu ,\tau _{T},\tau _{S})$ be a relaxed scenario that explains $(G,\sigma )$, i.e., $G = G_{_{<}}({\mathcal {S}})$. By definition, $V(G) = V(\digamma ({\mathcal {S}})) = L(T)$. Hence it remains to show that $E(G) \subseteq E(\digamma ({\mathcal {S}}))$. To this end, consider $ab \in E(G)$ and assume, for contradiction, that $ab\notin E(\digamma ({\mathcal {S}}))$. Let $A :=\sigma (a)$ and $B:=\sigma (b)$. By Lemma  13, ${{\,\mathrm{lca}\,}}_S(A,B) \preceq _S \mu ({{\,\mathrm{lca}\,}}_T(a,b))$. But then, by Definitions 3 and 4, $\tau _{S}({{\,\mathrm{lca}\,}}_S(A,B)) \le \tau _{S}({{\,\mathrm{lca}\,}}_T(a,b))$, implying $ab\notin E(G)$, a contradiction. $\square$

Since we only have that *xy* is an edge in $\digamma ({\mathcal {S}})$ if the path connecting *x* and *y* in the tree *T* of ${\mathcal {S}}$ contains a transfer edge, Theorem 4 immediately implies

#### Corollary 6

For every relaxed scenario ${\mathcal {S}}=(T,S,\sigma ,\mu ,\tau _{T},\tau _{S})$ without transfer edges, it holds that $E(G_{_{<}}({\mathcal {S}})) = \emptyset$.

Theorem 4 provides the formal justification for indirect phylogenetic approaches to HGT inference that are based on the work of Lawrence and Hartl (1992), Clarke et al. (2002), and Novichkov et al. (2004) by showing that $xy\in E(G_{_{<}}({\mathcal {S}}))$ can be explained only by HGT, irrespective of how complex the true biological scenario might have been. However, it does not cover all HGT events. Figure 7 shows that there are relaxed scenarios ${\mathcal {S}}$ for which $G_{_{<}}({\mathcal {S}}) \ne \digamma ({\mathcal {S}})$ even though $\digamma ({\mathcal {S}})$ is properly colored. Moreover, it is possible that an rs-Fitch graph $(G,\sigma )$ contains edges $xy\in E(G)$ with $\sigma (x)=\sigma (y)$. In particular, therefore, an rs-Fitch graph is not always an LDT graph.

It is natural, therefore, to ask whether for every properly colored Fitch graph there is a relaxed scenario ${\mathcal {S}}$ such that $G_{_{<}}({\mathcal {S}}) = \digamma ({\mathcal {S}})$. An affirmative answer is provided by

#### Theorem 5

The following statements are equivalent.

1. $(G,\sigma )$ is a properly colored complete multipartite graph.
2. There is a relaxed scenario ${\mathcal {S}}=(T,S,\sigma ,\mu ,\tau _{T},\tau _{S})$ with coloring $\sigma$ such that $G=G_{_{<}}({\mathcal {S}}) = \digamma ({\mathcal {S}})$.
3. $(G,\sigma )$ is complete multipartite and an LDT graph.
4. $(G,\sigma )$ is properly colored and an rs-Fitch graph.

In particular, for every properly colored complete multipartite graph $(G,\sigma )$ the triple set ${\mathfrak {S}}(G,\sigma )$ is compatible.

#### Proof

*(1) implies (2)*. We assume that $(G,\sigma )$ is a properly colored multipartite graph and set $L:=V(G)$ and $E:=E(G)$. If $E=\emptyset$, then the relaxed scenario ${\mathcal {S}}$ constructed in the proof of Lemma 4 satisfies $G=G_{_{<}}({\mathcal {S}}) = \digamma ({\mathcal {S}})$, i.e., the graphs are edgeless. Hence, we assume that $E\ne \emptyset$ and explicitly construct a relaxed scenario ${\mathcal {S}}= (T,S,\sigma ,\mu ,\tau _{T},\tau _{S})$ with coloring $\sigma$ such that $G=G_{_{<}}({\mathcal {S}}) = \digamma ({\mathcal {S}})$.

The graph $(G,\sigma )$ is properly colored and complete multipartite by assumption. Let $I_1,\dots , I_k$ denote the independent sets of *G*. Since $E\ne \emptyset$, we have $k>1$. Since all $x\in I_i$ are adjacent to all $y\in I_j$, $i\ne j$ and $(G,\sigma )$ is properly colored, it must hold that $\sigma (I_i)\cap \sigma (I_j)=\emptyset$. For a fixed *i* let $v_i^1,\dots v_i^{|I_i|}$ denote the elements in $I_i$.

We first start with the construction of the species tree *S*. First we add a planted root $0_S$ with child $\rho _S$. Vertex $\rho _S$ has children $w_1,\dots , w_k$ where each $w_j$ corresponds to one $I_j$. Note, $\sigma :L\rightarrow M$ may not be surjective, in which case we would add one additional child *x* to $\rho _S$ for each color $x\in M{\setminus } \sigma (L)$.

If $|\sigma (I_j)| = 1$, then we identify the single color $x\in \sigma (I_j)$ with $w_j$. Otherwise, i.e., if $|\sigma (I_j)| > 1$, vertex $w_j$ has as children the set ${{\,\mathrm{child}\,}}_S(w_j)=\sigma (I_j)$ which are leaves in *S*. See Fig. 18 for an illustrative example. Now we can choose the time map $\tau _{S}$ for *S* such $\tau _{S}(0_S)=3$, $\tau _{S}(\rho _S)=2$, $\tau _{S}(x)=0$ for all $x\in L(S)$ and $\tau _{S}(x)=1$ for all $x\in V^0(S){\setminus }\{\rho _S\}$.

We now construct *T* as follows. The tree *T* has planted root $0_T$ with child $\rho _T$. Vertex $\rho _T$ has *k* children $u_1,\dots , u_k$ where each $u_j$ corresponds to one $I_j$. Vertex $u_j$ is a leaf if $|I_j|=1$, and, otherwise, has exactly $|I_j|$ children that are uniquely identified with the elements in $I_j$.

We now define the time map $\tau _{T}$ and reconciliation map $\mu$ for $v\in V(T)$:

$$\begin{aligned} \tau _{T}(v) :={\left\{ \begin{array}{ll} 3=\tau _{S}(0_S) \\ 0 \\ 1.5 \\ 1.25 \end{array}\right. } \mu (v) :={\left\{ \begin{array}{ll} 0_S &{}\text {if } v=0_T,\\ \sigma (v) &{}\text {if } v\in L(T),\\ (\rho _S,w_1) &{}\text {if } v = \rho _T, \text { and}\\ (\rho _S,w_i) &{}\text {if } v=u_i\not \in L(T), 1\le i\le k. \end{array}\right. } \end{aligned}$$

With the help of Fig. 18 it is now easy to verify that (i) $\tau _{T}$ is a time map for *T*, and that (ii) the reconciliation map $\mu$ is time-consistent. In summary the constructed ${\mathcal {S}}=(T,S,\sigma ,\mu ,\tau _{T},\tau _{S})$ is a relaxed scenario.

We continue with showing that $E=E(G_{_{<}}({\mathcal {S}}))=E(\digamma ({\mathcal {S}}))$. To this end, let $a,b\in L$ be two vertices. Note, $ab\in E$ if and only if $a\in I_i$ and $b\in I_j$ for distinct $i,j\in [k]:=\{1,2,\ldots ,k\}$.

First assume that $ab\in E$ and thus, $a\in I_i$ and $b\in I_j$ for distinct $i,j\in [k]$. By construction, $a\preceq _{T}u_i\ne u_j\succeq _{T} b$ with ${{\,\mathrm{lca}\,}}_{T}(u_i,u_j)=\rho _{T}$. In particular, we have ${{\,\mathrm{par}\,}}_T(u_i)={{\,\mathrm{par}\,}}_T(u_j)=\rho _{T}$ and the path from *a* to *b* contains the two edges $(\rho _{T},u_i)$ and $(\rho _{T},u_j)$. By construction, we have $\mu (\rho _T)=(\rho _{S},w_1)$, and for all $1\le l\le k$, $\mu (u_l)=\sigma (u_l)=w_l$ if $u_l$ is a leaf, and $\mu (u_l)=(\rho _S,w_l)$ otherwise. These two arguments imply that $\mu (\rho _T)$ and $\mu (u_l)$ are comparable if and only if $u_l=u_1$. Now, since $u_i\ne u_j$, they cannot both be equal to $u_1$ and thus, at least one of the edges $(\rho _{T},u_i)$ and $(\rho _{T},u_j)$ is a transfer edge. Hence, $ab\in E(\digamma ({\mathcal {S}}))$. By construction, $ab\in E$ implies ${{\,\mathrm{lca}\,}}_T(a,b)=\rho _T$. Hence, we have $\mu ({{\,\mathrm{lca}\,}}_T(a,b)) = \mu (\rho _T)=(\rho _S,w_1)\prec _S\rho _S = {{\,\mathrm{lca}\,}}_S(\sigma (a),\sigma (b))$, and thus $ab\in E(G_{_{<}}({\mathcal {S}}))$.

Now assume that $ab\notin E$, and thus, $a,b\in I_i$ for some $i\in [k]$. It clearly suffices to consider the case $a\ne b$, and thus, $a,b\in {{\,\mathrm{child}\,}}_T(u_i)$ and $u_i\notin L(T)$ holds by construction. In particular, the path between *a* and *b* only consists of the edges $(u_i,a)$ and $(u_i,b)$. Moreover, we have $\sigma (a),\sigma (b)\preceq _{S} w_i$ and $\mu (u_i)=(\rho _S,w_i)$. Hence, none of the edges $(u_i,a)$ and $(u_i,b)$ is a transfer edge, and $ab\notin E(\digamma ({\mathcal {S}}))$. We have $\mu ({{\,\mathrm{lca}\,}}_{T}(a,b))=(\rho _S,w_i)\succ _T w_i \succeq _{T} {{\,\mathrm{lca}\,}}_{S}(\sigma (a),\sigma (b))$, and thus $\tau _{T}({{\,\mathrm{lca}\,}}_{T}(a,b))> \tau _{S}({{\,\mathrm{lca}\,}}_{S}(\sigma (a),\sigma (b)))$. Hence, $ab\notin E(G_{_{<}}({\mathcal {S}}))$.

In summary, $ab\in E$ if and only if $ab\in E(\digamma ({\mathcal {S}}))$ if and only if $ab\in E(G_{_{<}}({\mathcal {S}}))$, and consequently, $G=G_{_{<}}({\mathcal {S}}) = \digamma ({\mathcal {S}})$.

*(2) implies (1).* Thus, suppose that there is a relaxed scenario ${\mathcal {S}}=(T,S,\sigma ,\mu ,\tau _{T},\tau _{S})$ with coloring $\sigma$ such that $G=G_{_{<}}({\mathcal {S}}) = \digamma ({\mathcal {S}})$. Proposition 3 implies that $(G,\sigma )=(G_{_{<}}({\mathcal {S}}),\sigma )$ is properly colored. Moreover, $(G,\sigma )=(\digamma ({\mathcal {S}}),\sigma )$ is an rs-Fitch graph and thus, by Observation 1, *G* is complete multipartite.

Statements (1) and (2) together with Proposition 5 imply (3). Conversely, if (3) is satisfied then Proposition 3 implies that $(G,\sigma )$ is properly colored. This and the fact that *G* is complete multipartite implies (1). Therefore, Statements (1), (2) and (3) are equivalent.

Furthermore, (4) implies (1) by Observation 1. Conversely, $(G,\sigma )$ in Statement (2) is an rs-Fitch graph and an LDT graph. Hence it is properly colored by Proposition 3. Thus (2) implies (4).

Statement (3), in particular, implies that every properly colored complete multipartite $(G,\sigma )$ is an LDT graph and, thus, there is a relaxed scenario ${\mathcal {S}}$ such that $G=G_{_{<}}({\mathcal {S}})$. Now, we can apply Lemma 6 to conclude that ${\mathfrak {S}}(G,\sigma )$ is compatible, which completes the proof. $\square$

#### Corollary 7

A colored graph $(G,\sigma )$ is an LDT graph and an rs-Fitch graph if and only if $(G,\sigma )$ is a properly colored complete multipartite graph (and thus, a properly colored Fitch graph for some 0/1-edge-labeled tree).

#### Proof

If $(G,\sigma )$ is an rs-Fitch graph then, by Observation 1, *G* is a complete multipartite graph. Moreover, since $(G,\sigma )$ is an LDT graph, $(G,\sigma )$ is properly colored (cf. Proposition 3). Conversely, if $(G,\sigma )$ is a properly colored complete multipartite graph it is, by Theorem 5(2), an rs-Fitch graph and an LDT graph. Now the equivalence between Statements (1) and (3) in Theorem 5 shows that $(G,\sigma )$ is an LDT graph. $\square$

#### Corollary 8

Let $(G,\sigma )$ be a vertex-colored graph. If ${\mathfrak {T}}(G) = \emptyset$ and ${\mathfrak {S}}(G,\sigma )$ is incompatible, then *G* is a complete multipartite graph (and thus, a Fitch graph for some 0/1-edge-labeled tree), but $\sigma$ is not a proper vertex coloring of *G*.

#### Proof

By definition, if ${\mathfrak {T}}(G)=\emptyset$, then *G* cannot contain an induced $K_2+K_1$. By Proposition 5, *G* is a Fitch graph. Contraposition of the last statement in Theorem 5 and *G* being a Fitch graph for some $(T,\lambda )$ implies that $\sigma$ is not a proper vertex coloring of *G*. $\square$

As outlined in the main part of this paper, LDT graphs are sufficient to describe replacing HGT. They fail, however, to describe additive HGT in full detail.

### rs-Fitch graphs with general colorings

In scenarios with additive HGT, the rs-Fitch graph is no longer properly colored and no-longer coincides with the LDT graph. Since not every vertex-colored complete multipartite graphs $(G,\sigma )$ is an rs-Fitch graph (cf. Theorem 6), we ask whether an LDT graph $(G,\sigma )$ that is not itself already an rs-Fitch graph imposes constraints on the rs-Fitch graphs $(\digamma ({\mathcal {S}}),\sigma )$ that derive from relaxed scenarios ${\mathcal {S}}$ that explain $(G,\sigma )$. As a first step towards this goal, we aim to characterize rs-Fitch graphs, i.e., to understand the conditions imposed by the existence of an underlying scenario ${\mathcal {S}}$ on the compatibility of the collection of independent sets ${\mathscr {I}}$ of *G* and the coloring $\sigma$. As we shall see, these conditions can be explained in terms of an auxiliary graph that we introduce in a very general setting:

#### Definition 17

Let *L* be a set, $\sigma :L\rightarrow M$ a map and ${\mathscr {I}}=\{I_1,\dots , I_k\}$ a set of subsets of *L*. Then the graph ${\mathcal {A}}_{\digamma }(\sigma ,{\mathscr {I}})$ has vertex set *M* and edges *xy* if and only if $x\ne y$ and $x,y\in \sigma (I')$ for some $I'\in {\mathscr {I}}$. We define an edge labeling $\ell : E({\mathcal {A}}_{\digamma }) \rightarrow 2^{{\mathscr {I}}}$ such that $\ell (e) :=\{I\in {\mathscr {I}}\mid \exists x,y\in I \text { s.t. } \sigma (x)\sigma (y)=e\}$.

By construction ${\mathcal {A}}_{\digamma }(\sigma ,\mathscr {I'})$ is a subgraph of ${\mathcal {A}}_{\digamma }(\sigma ,{\mathscr {I}})$ whenever $\mathscr {I'}\subseteq {\mathscr {I}}$. The labeling of an edge *e* records the sets $I\in {\mathscr {I}}$ that imply the presence of the edge.

#### Theorem 6

A graph $(G,\sigma )$ is an rs-Fitch graph if and only if (i) it is complete multipartite with independent sets ${\mathscr {I}}=\{I_1,\dots , I_k\}$, and (ii) if $k>1$, there is an independent set $I'\in {\mathscr {I}}$ such that ${\mathcal {A}}_{\digamma }(\sigma ,{\mathscr {I}}{\setminus }\{I'\})$ is disconnected.

#### Proof

Let $G=(L,E)$ be a graph with coloring $\sigma :L\rightarrow M$. Suppose first that *G* satisfies (i) and (ii). To show that $(G,\sigma )$ is an rs-Fitch graph, we will construct a relaxed scenario ${\mathcal {S}}=(T,S,\sigma ,\mu ,\tau _{T},\tau _{S})$ such that $G = \digamma ({\mathcal {S}})$. If $k=1$, or equivalently $E=\emptyset$, then the relaxed scenario ${\mathcal {S}}$ constructed in the proof of Lemma 4 satisfies $G=\digamma ({\mathcal {S}})$, i.e., both graphs are edgeless. Now assume that $k>1$ and thus, $E\ne \emptyset$. Hence, we can choose an independent set $I'\in {\mathscr {I}}$ such that ${\mathcal {A}}_{\digamma }':={\mathcal {A}}_{\digamma }(\sigma ,{\mathscr {I}}{\setminus }\{I'\})$ is disconnected. Note that ${\mathscr {I}}{\setminus }\{I'\}$ is non-empty since $k>1$. Moreover, since ${\mathcal {A}}_{\digamma }'$ is a disconnected graph on the color set *M*, there is a connected component *C* of ${\mathcal {A}}_{\digamma }'$ such that $(M{\setminus } C) \cap \sigma (I')\ne \emptyset$. Hence $M_1:=M{\setminus } C$ and $M_2:=C$ form a bipartition of *M* such that neither $M_1$ nor $M_2$ are empty sets.

We continue by showing that every $I\in {\mathscr {I}}{\setminus } \{I'\}$ satisfies either $\sigma (I)\subseteq M_1$ or $\sigma (I)\subseteq M_2$. To see this, assume, for contradiction, that there are colors $A\in \sigma (I)\cap M_1$ and $B\in \sigma (I)\cap M_2$ for some $I\in {\mathscr {I}}{\setminus } \{I'\}$. Thus, $B\in C$ and, by definition, $AB\in E({\mathcal {A}}_{\digamma }')$. Therefore, *A* and *B* must lie in the connected component *C*; a contradiction. Therefore, we can partition ${\mathscr {I}}{\setminus } \{I'\}$ into ${\mathscr {I}}_1:=\{I\in {\mathscr {I}}{\setminus } \{I'\} \mid \sigma (I)\subseteq M_1\}$ and ${\mathscr {I}}_2:=\{I\in {\mathscr {I}}{\setminus } \{I'\} \mid \sigma (I)\subseteq M_2\}$. Note that one of the sets ${\mathscr {I}}_1$ and ${\mathscr {I}}_2$, but not both of them, may be empty. This may be the case, for instance, if $\sigma$ is not surjective.

Now, we construct a relaxed scenario ${\mathcal {S}}= (T,S,\sigma ,\mu ,\tau _{T},\tau _{S})$ with coloring $\sigma$ such that $G=\digamma ({\mathcal {S}})$. We first define the species tree *S* as the planted tree where $\rho _{S}$ (i.e. the single child of $0_S$) hast two children $w_1$ and $w_2$. If $|M_1|=1$, we identify $w_1$ with the single element in $M_1$, and otherwise, we set ${{\,\mathrm{child}\,}}_S(w_1)=L(S(w_1)):=M_1$. We proceed analogously for $w_2$ and $M_2$. Thus, *S* is phylogenetic by construction. We choose the time map $\tau _{S}$ by putting $\tau _{S}(0_S)=2$, $\tau _{S}(\rho _S)=1$, $\tau _{S}(w_1)=\tau _{S}(w_2)=0.5$ and $\tau _{S}(x)=0$ for all $x\in L(S)$. This completes the construction of *S* and $\tau _{S}$.

We proceed with the construction of the gene tree *T*, its time map $\tau _{T}$ and the reconciliation map $\mu$. This tree *T* has leaf set *L*, planted root $0_T$, and root $\rho _T$. We set $\mu (0_T)=0_S$ and $\tau _{T}(0_T)=\tau _{S}(0_S)=2$, and moreover $\mu (x)=\sigma (x)$ and $\tau _{T}(x)=0$ for all $x\in L$.

For each $I_j\in {\mathscr {I}}{\setminus }\{I'\}$, we add a vertex $u_j$. We will later specify how these vertices are connected (via paths) to $\rho _T$. If $|I_j|=1$, $u_j$ becomes a leaf of *T* that is identified with the unique element in $I_j$. Otherwise, we add exactly $|I_j|$ children to $u_j$, each of which is identified with one of the elements in $I_j$. If $u_j$ is a leaf, we already defined $\mu (u_j)=\sigma (u_j)$ and $\tau _{T}(u_j)=0$.

Otherwise, we set $\tau _{T}(u_j)=0.6$ and $\mu (u_j)=(\rho _S,w_1)$ if $I_j\in {\mathscr {I}}_1$ and $\mu (u_j)=(\rho _S,w_2)$ if $I_j\in {\mathscr {I}}_2$. Recall that $M_1\cap \sigma (I')\ne \emptyset$. However, both $M_2\cap \sigma (I')\ne \emptyset$ and $M_2\cap \sigma (I')=\emptyset$ are possible. The latter case appears e.g. whenever ${\mathcal {A}}_{\digamma }(\sigma ,{\mathscr {I}})$ was already disconnected. To connect the vertices $u_j$ to $\rho _T$, we distinguish the three mutually exclusive cases:

*Case (a):* $$M_2\cap \sigma (I')=\emptyset $$ *and* $${\mathscr {I}}_1\ne \emptyset $$.

We set $\mu (\rho _T)=(\rho _S,w_2)$ and $\tau _{T}(\rho _T)=0.9$. We attach all $u_j$ that correspond to elements $I_j\in {\mathscr {I}}_1$ as children of $\rho _T$. If $|I'|> 1$ or ${\mathscr {I}}_2\ne \emptyset$, we create a vertex $u'$ to which all elements in $I'$ and all $u_j$ such that $I_j\in {\mathscr {I}}_2$ are attached as children, attach $u'$ as a child of $\rho _T$, and set $\mu (u')=(\rho _S,w_1)$ and $\tau _{T}(u')=0.75$. Otherwise, we simply attach the single element $x'$ in $I'$ as a child of $\rho _T$. Clearly, the so constructed tree *T* is phylogenetic. Note that the edges $(\rho _T, u_j)$ with $I_j\in {\mathscr {I}}_1$ as well as the edges $(u',u_j)$ with $I_j\in {\mathscr {I}}_2$ are transfer edges. Together with $(\rho _T,u')$ or $(\rho _T,x)$, respectively, these are the only transfer edges.

*Case (b):* $$M_2\cap \sigma (I')=\emptyset $$ *and* $${\mathscr {I}}_1=\emptyset $$.

By the arguments above, the latter implies ${\mathscr {I}}_2\ne \emptyset$. Hence, we can set $\mu (\rho _{T})=(\rho _S,w_1)$ and $\tau _{T}(\rho _T)=0.9$ and attach all elements of $I'$ as well as the vertices $u_j$ corresponding to the independent sets $I_j\in {\mathscr {I}}_2={\mathscr {I}}{\setminus } \{I'\}$ as children of $\rho _T$. Since $|I'|\ge 1$ and ${\mathscr {I}}_2\ge 1$, the tree *T* obtained in this manner is again phylogenetic. Moreover, note that the transfer edges are exactly the edges $(\rho _T,u_j)$.

*Case (c):* $$M_2\cap \sigma (I')\ne \emptyset $$.

In this case, the sets $I'_1:=\{x\in I'\mid \sigma (x)\in M_1\}$ and $I'_2:=\{x\in I'\mid \sigma (x)\in M_2\}$ must be non-empty. We set $\mu (\rho _T)=(0_T,\rho _T)$ and $\tau _{T}(\rho _T)=1.5$. If $|I'_1|> 1$ or ${\mathscr {I}}_2\ne \emptyset$, we create a vertex $u'$ to which all elements in $I'_1$ and all $u_j$ such that $I_j\in {\mathscr {I}}_2$ are attached as children, and set $\mu (u')=(\rho _S,w_1)$ and $\tau _{T}(u')=0.75$. Otherwise, we simply attach the single element in $I'_1$ as a child of $\rho _T$. For the “other side”, we proceed analogously: If $|I'_2|> 1$ or ${\mathscr {I}}_1\ne \emptyset$, we create a vertex $u''$ to which all elements in $I'_2$ and all $u_j$ such that $I_j\in {\mathscr {I}}_1$ are attached as children, and set $\mu (u')=(\rho _S,w_2)$ and $\tau _{T}(u'')=0.75$. Otherwise, we simply attach the single element in $I'_2$ as a child of $\rho _T$. By construction, the so constructed tree is again phylogenetic. Moreover, the transfer edges are exactly the edges $(u',u_j)$ and $(u'',u_j)$.

Using Fig. 19, one can easily verify that, in all three Cases (a)-(c), the reconciliation map $\mu$ is time-consistent with $\tau _{T}$ and $\tau _{S}$. Thus, ${\mathcal {S}}$ is a relaxed scenario. Moreover, Fig. 19 together with the fact that $\sigma (I)\subseteq M_1$ holds for all $I\in {\mathscr {I}}_1$, and $\sigma (I)\subseteq M_2$ holds for all $I\in {\mathscr {I}}_2$, shows that $G=\digamma ({\mathcal {S}})$ in all three cases. Hence, $(G,\sigma )$ is an rs-Fitch graph.

For the *only-if*-direction, assume that $(G=(V,E),\sigma )$ is an rs-Fitch graph. Hence, there exists a relaxed scenario ${\mathcal {S}}=(T,S,\sigma ,\mu ,\tau _{T},\tau _{S})$ such that $G = \digamma ({\mathcal {S}})$. By Observation 1 and Proposition 5, $(G,\sigma )$ is a complete multipartite graph that is determined by its set of independent sets ${\mathscr {I}}=\{I_1,\dots ,I_k\}$. Hence, Condition (i) is satisfied.

Now assume, for contradiction, that Condition (ii) is violated. Thus $k\ge 2$ and there is no independent set $I'\in {\mathscr {C}}$ such that ${\mathcal {A}}_{\digamma }(\sigma ,{\mathscr {I}}{\setminus }\{I'\})$ is disconnected. If $|M|=1$, then the species tree *S* only consists of the planted root $0_S$ and the root $\rho _S$, which in this case is identified with the single element in *M*. Clearly, all vertices and edges are comparable in such a tree *S*, and hence, there is no transfer edges in ${\mathcal {S}}$, implying $E = \emptyset$ and thus $|{\mathscr {I}}| = 1$; a contradiction to $k\ge 2$.

Thus we have $|M|\ge 2$ and the root $\rho _S$ of the species tree *S* has at least two children. Since ${\mathcal {A}}_{\digamma }(\sigma ,{\mathscr {I}}{\setminus }\{I'\})$ is connected for every $I'\in {\mathscr {C}}$, the graph ${\mathcal {A}}_{\digamma }(\sigma ,{\mathscr {I}})$ is also connected. Since each color appears at most once as a leaf of *S*, $\sigma (L(S(v_1))) \cap \sigma (L(S(v_2)))=\emptyset$ holds for any two distinct children $v_1,v_2\in {{\,\mathrm{child}\,}}_S (\rho _S)$. These three assertions, together with the definition of the auxiliary graph ${\mathcal {A}}_{\digamma }(\sigma ,{\mathscr {I}})$, imply that there are two distinct colors $A, B\in M$ such that *AB* is an edge in ${\mathcal {A}}_{\digamma }(\sigma ,{\mathscr {I}})$, $A\preceq _S v_1$ and $B\prec _{S} v_2$ for distinct children $v_1,v_2\in {{\,\mathrm{child}\,}}_S (\rho _S)$. By definition of ${\mathcal {A}}_{\digamma }(\sigma ,{\mathscr {I}})$ there is an independent set $I'\in {\mathscr {I}}$ containing a vertex $a\in I'$ with $\sigma (a)=A$ and a vertex $b\in I'$ with $\sigma (b)=B$. Since *a* and *b* lie in the same independent set, we have $ab\notin E$. By Lemma 13, $\mu ({{\,\mathrm{lca}\,}}_T(a,b)) \succeq _S {{\,\mathrm{lca}\,}}_S(A,B)=\rho _S$. Since, by assumption, ${\mathcal {A}}_{\digamma }(\sigma ,{\mathscr {I}}{\setminus }\{I'\})$ is also connected, we find two distinct colors *C* and *D* (not necessarily distinct from *A* and *B*) such that *CD* is an edge in ${\mathcal {A}}_{\digamma }(\sigma ,{\mathscr {I}})$, $C\preceq _S v_3$ and $D\prec _{S} v_4$ for distinct children $v_3,v_4\in {{\,\mathrm{child}\,}}_S (\rho _S)$ (but not necessarily distinct from $v_1$ and $v_2$), and in particular, an independent set $I''\in {\mathscr {I}}{\setminus } \{I'\}$ containing a vertex $c\in I''$ with $\sigma (c)=C$ and a vertex $d\in I''$ with $\sigma (d)=D$. By construction, $I'\ne I''$, and thus, all edges between $I'$ and $I''$ exist in *G*, in particular the edges *ac*, *ad*, *bc*, *bd*. Since $c,d\in I''$, we have $cd\notin E$ and thus, by Lemma 13, $\mu ({{\,\mathrm{lca}\,}}_T(c,d)) \succeq _S {{\,\mathrm{lca}\,}}_S(C,D)=\rho _S$.

We now consider the unique path *P* in *T* that connects ${{\,\mathrm{lca}\,}}_T(a,b)$ and ${{\,\mathrm{lca}\,}}_T(c,d)$. Since $\mu$ is time-consistent and $\mu ({{\,\mathrm{lca}\,}}_T(a,b)), \mu ({{\,\mathrm{lca}\,}}_T(c,d)) \succeq _S \rho _S$, we conclude that, for every edge *uv* along this path *P*, we have $\mu (u), \mu (v)\succeq _S \rho _S$ and thus $\mu (u), \mu (v)\in \{\rho _S, (0_S,\rho _S)\}$. But then, $\mu (u)$ and $\mu (v)$ are comparable in *S*. Therefore, *P* does not contain any transfer edge. Since $ab\notin E$, the path connecting *a* and ${{\,\mathrm{lca}\,}}_{T}(a,b)$ does not contain any transfer edges. Likewise, $cd\notin E$ implies that the path connecting *c* and ${{\,\mathrm{lca}\,}}_{T}(c,d)$ does not contain any transfer edges. Thus, the path connecting *a* and *c* also does not contain any transfer edge, which implies that $ac\notin E(\digamma ({\mathcal {S}}))=E$; a contradiction since *a* and *c* belong to two distinct independent sets.

Hence, we conclude that for $k>1$ there exists an independent set $I'\in {\mathscr {C}}$ such that ${\mathcal {A}}_{\digamma }(\sigma ,{\mathscr {I}}{\setminus }\{I'\})$ is disconnected. $\square$

#### Corollary 9

rs-Fitch graphs can be recognized in polynomial time.

#### Proof

Every rs-Fitch graph $(G,\sigma )$ must be complete multipartite, which can be verified in polynomial time. In this case, the set of independent sets ${\mathscr {I}}=\{I_1,\dots , I_k\}$ of *G* can also be determined and the graph ${\mathcal {A}}_{\digamma }(\sigma ,{\mathscr {I}})$ can be constructed in polynomial time. Finally, we need to find an independent set $I'\in {\mathscr {I}}$, such that ${\mathcal {A}}_{\digamma }(\sigma ,{\mathscr {I}}{\setminus }\{I'\})$ is disconnected. Clearly, checking whether ${\mathcal {A}}_{\digamma }(\sigma ,{\mathscr {I}}{\setminus }\{I'\})$ is disconnected can be done in polynomial time and since there are at most |*V*(*G*)| independent sets in ${\mathscr {I}}$, finding an independent set $I'$ such that ${\mathcal {A}}_{\digamma }(\sigma ,{\mathscr {I}}{\setminus }\{I'\})$ is disconnected (if one exists) can be done in polynomial time as well. $\square$

#### Corollary 10

Let $(G,\sigma )$ be a complete multipartite graph with coloring $\sigma :V(G) \rightarrow M$ and set of independent sets ${\mathscr {I}}$. Then, $(G,\sigma )$ is an rs-Fitch graph if and only if ${\mathcal {A}}_{\digamma }(\sigma ,{\mathscr {I}})$ is disconnected or there is a cut $Q\subseteq E({\mathcal {A}}_{\digamma }(\sigma ,{\mathscr {I}}))$ such that all edges $e\in Q$ have the same label $\ell (e)=\{I\}$ for some $I\in {\mathscr {I}}$.

#### Proof

If ${\mathcal {A}}_{\digamma }(\sigma ,{\mathscr {I}})$ is disconnected, then ${\mathcal {A}}_{\digamma }(\sigma ,{\mathscr {I}}{\setminus } \{I\})$ remains disconnected for all $I\in {\mathscr {I}}$ and, by Theorem 6, $(G,\sigma )$ is an rs-Fitch graph.

If there is a cut $Q\subseteq E({\mathcal {A}}_{\digamma }(\sigma ,{\mathscr {I}}))$ such that all edges $e\in Q$ have the same label $\ell (e)=\{I\}$ for some $I\in {\mathscr {I}}$, then, by definition, $E({\mathcal {A}}_{\digamma }(\sigma ,{\mathscr {I}}{\setminus } \{I\}))\subseteq E':=E({\mathcal {A}}_{\digamma }(\sigma ,{\mathscr {I}})){\setminus } Q$. Since *Q* is a cut in ${\mathcal {A}}_{\digamma }(\sigma ,{\mathscr {I}})$, the resulting graph ${\mathcal {A}}_{\digamma }'= (M,E')$ is disconnected. By the latter arguments, ${\mathcal {A}}_{\digamma }(\sigma ,{\mathscr {I}}{\setminus } \{I\})$ is a subgraph of ${\mathcal {A}}_{\digamma }'$, and thus, disconnected as well. By Theorem 6, $(G,\sigma )$ is an rs-Fitch graph.

Conversely, if $(G,\sigma )$ is an rs-Fitch graph, then Theorem 6 implies that ${\mathcal {A}}_{\digamma }(\sigma ,{\mathscr {I}}{\setminus } \{I\})$ is disconnected for some $I\in {\mathscr {I}}$. If ${\mathcal {A}}_{\digamma }(\sigma ,{\mathscr {I}})$ was already disconnected, then there is nothing to show. Hence assume that ${\mathcal {A}}_{\digamma }(\sigma ,{\mathscr {I}}) = (M,E)$ is connected and let ${\mathcal {A}}_{\digamma }(\sigma ,{\mathscr {I}}{\setminus } \{I\}) = (M,E')$. Moreover, let $F\subseteq E$ be the subset of edges $e\in E$ with $I\in \ell (e)$. Note, *F* contains all edges of *E* that have potentially been removed from *E* to obtain $E'$. However, all edges $e=xy$ in *F* with $|\ell (e)|>1$ must remain in ${\mathcal {A}}_{\digamma }(\sigma ,{\mathscr {I}}{\setminus } \{I\})$, since there is another independent set $I'\in \ell (e){\setminus } \{I\}$ such that $x,y\in \sigma (I')$. Hence, only those edges *e* in *F* for which $|\ell (e)|=1$ are removed from *E*. Hence, there is a cut $Q\subseteq F\subseteq E$ such that all edges $e\in Q$ have the same label $\ell (e)=\{I\}$ for some $I\in {\mathscr {I}}$. $\square$

#### Corollary 11

If $(G,\sigma )$ with coloring $\sigma :V(G) \rightarrow M$ is an rs-Fitch graph, then there are no two disjoint independent sets *I* and $I'$ of *G* with $\sigma (I)=\sigma (I')= M$.

#### Proof

Let ${\mathscr {I}}$ be the set of independent sets of *G*. If $|{\mathscr {I}}|=1$, there is nothing to show and thus, we assume that $|{\mathscr {I}}|>1$. Assume, for contradiction, that there are two distinct independent sets $I, I' \in {\mathscr {I}}$ such that $\sigma (I)=\sigma (I')= M$. For every $I''\in {\mathscr {I}}$, the set ${\mathscr {I}}{\setminus } \{I''\}$ clearly contains at least one of the two sets *I* and $I'$, both of which contain all colors in *M*. Therefore, ${\mathcal {A}}_{\digamma }(\sigma , {\mathscr {I}}{\setminus } \{I''\})$ is the complete graph by construction and, thus, connected for every $I''\in {\mathscr {I}}$. This together with Theorem 6 implies that $(G,\sigma )$ is not an rs-Fitch graph; a contradiction. $\square$

#### Corollary 12

Every complete multipartite graph $(G,\sigma )$ with a vertex coloring $\sigma :V(G) \rightarrow M$ that is not surjective is an rs-Fitch graph.

#### Proof

If $\sigma :V(G) \rightarrow M$ is not surjective, then ${\mathcal {A}}_{\digamma }(\sigma ,{\mathscr {I}})$ is disconnected, where ${\mathscr {I}}$ denotes the set of independent sets of *G*. Hence, if $k>1$, then ${\mathcal {A}}_{\digamma }(\sigma ,{\mathscr {I}}{\setminus } \{I\})$ remains disconnected for all $I\in {\mathscr {I}}$. By Theorem 6, $(G,\sigma )$ is an rs-Fitch graph. $\square$

Corollary 12 may seem surprising since it implies that the property of being an rs-Fitch graph can depend on species (colors *M*) for which we have no genes *L* in the data. The reason is that an additional lineage in the species tree provides a place to “park” interior vertices in the gene tree from which HGT-edges can emanate that could not always be accommodated within lineages that have survivors—where they may force additional HGT edges.

#### Corollary 13

Every Fitch graph $(G,\sigma )$ that contains an independent set *I* and a vertex $x\in I$ with $\sigma (x)\notin \sigma (I')$ for all other independent sets $I'\ne I$, is an rs-Fitch graph.

#### Proof

Let ${\mathscr {I}}$ denote the set of independent sets of *G*. If there is an independent set $I\in {\mathscr {I}}$ that contains a vertex $x\in I$ with $\sigma (x)\notin \sigma (I')$ for all other independent sets $I'\ne I$, then the vertex $\sigma (x)$ in ${\mathcal {A}}_{\digamma }(\sigma ,{\mathscr {I}}{\setminus } \{I\})$ is an isolated vertex and thus, ${\mathcal {A}}_{\digamma }(\sigma ,{\mathscr {I}}{\setminus } \{I\})$ is disconnected. By Theorem 6, $(G,\sigma )$ is an rs-Fitch graph. $\square$

As for LDT graphs, the property of being an rs-Fitch graph is hereditary.

#### Corollary 14

If $(G=(L,E),\sigma )$ is an rs-Fitch graph, then the colored vertex induced subgraph $(G[W],\sigma _{|W})$ is an rs-Fitch graph for all non-empty subsets $W\subseteq L$.

#### Proof

It suffices to show the statement for $W = L{\setminus }\{x\}$ for an arbitrary vertex $x\in L$. If $G=(L,E)$ is edgeless, then *G*[*W*] is edgeless and thus, by Theorem 6, an rs-Fitch graph.

Thus, assume that $E\ne \emptyset$ and thus, for the set ${\mathscr {I}}$ of independent sets of *G* it holds that $|{\mathscr {I}}|>1$. Since *G* does not contain an induced $K_2+K_1$, it is easy to see that *G*[*W*] cannot contain an induced $K_2+K_1$ and thus, *G*[*W*] is a complete multipartite graph. Hence, Theorem 6(i) is satisfied. Moreover, if for the set ${\mathscr {I}}'$ of independent sets of *G*[*W*] it holds that $|{\mathscr {I}}'|=1$ then, Theorem 6 already shows that $(G[W],\sigma _{|W})$ is an rs-Fitch graph.

Thus, assume that $|{\mathscr {I}}'|>1$. Now compare the labeling $\ell$ of the edges in ${\mathcal {A}}_{\digamma }= {\mathcal {A}}_{\digamma }(\sigma , {\mathscr {I}})$ and the labeling $\ell '$ of the edges in ${\mathcal {A}}_{\digamma }' = {\mathcal {A}}_{\digamma }(\sigma _{|W}, {\mathscr {I}}')$. Note, ${\mathcal {A}}_{\digamma }$ and ${\mathcal {A}}_{\digamma }'$ have still the same vertex set *M*. Let $I\in {\mathscr {I}}$ with $x\in I$. For all vertices $y\in I$ with $\sigma (x)\ne \sigma (y)$, we have an edge $e =\sigma (x)\sigma (y)$ in ${\mathcal {A}}_{\digamma }$ and $I\in \ell (e)$. Consequently, for all edges *e* of ${\mathcal {A}}_{\digamma }$ that are present in ${\mathcal {A}}_{\digamma }'$ we have $\ell '(e)\subseteq \ell (e)$. In particular, ${\mathcal {A}}_{\digamma }'$ cannot have edges that are not present in ${\mathcal {A}}_{\digamma }$, since we reduced for one independent set the size by one. Therefore, ${\mathcal {A}}_{\digamma }'$ is a subgraph of ${\mathcal {A}}_{\digamma }$.

By Theorem 6, there is an independent set $I'\in {\mathscr {I}}$, not necessarily distinct from *I*, such that ${\mathcal {A}}_{\digamma }(\sigma ,{\mathscr {I}}{\setminus }\{I'\})$ is disconnected. If $I' = \{x\}$, then ${\mathscr {I}}' = {\mathscr {I}}{\setminus } \{I'\}$ and ${\mathcal {A}}_{\digamma }' ={\mathcal {A}}_{\digamma }$ must be disconnected as well. Otherwise, ${\mathcal {A}}_{\digamma }'\subseteq {\mathcal {A}}_{\digamma }$ and similar arguments as above show that ${\mathcal {A}}_{\digamma }(\sigma ,\mathscr {I'}{\setminus }\{I'\}) \subseteq {\mathcal {A}}_{\digamma }(\sigma ,{\mathscr {I}}{\setminus }\{I'\})$. Therefore, in both of the latter cases, ${\mathcal {A}}_{\digamma }(\sigma ,\mathscr {I'}{\setminus }\{I'\})$ is disconnected and Theorem 6 implies that $(G[W],\sigma _{|W})$ is an rs-Fitch graph. $\square$

As outlined in the main part of this paper, Corollary 14 is usually not satisfied if we restrict the codomain of $\sigma$ to the observable part of colors, even if $\sigma$ is surjective.

### Least resolved trees for Fitch graphs

It is important to note that the characterization of rs-Fitch graphs in Theorem 6 does not provide us with a characterization of rs-Fitch graphs that share a common relaxed scenario with a given LDT graph. As a potential avenue to address this problem we investigate the structure of least-resolved trees for Fitch graphs as possible source of additional constraints.

*All trees considered in this Appendix* B.4*are rooted and phylogenetic but not planted unless stated differently.* This is no loss of generality, since we are interested in Fitch-least-resolved trees, which are never be planted because the edge incident with the planted root can be contracted without affecting the paths between the leaves.

#### Definition 18

The edge-labeled tree $(T,\lambda )$ is *Fitch-least-resolved* w.r.t. $\digamma (T,\lambda )$, if for all trees $T'\ne T$ that are displayed by *T* and every labeling $\lambda '$ of $T'$ it holds that $\digamma (T,\lambda )\ne \digamma (T',\lambda ')$.

#### Definition 19

Let $(T,\lambda )$ be an edge-labeled tree and let $e=(x,y)\in E(T)$ be an inner edge. The tree $(T_{/e}, \lambda _{/e})$ with $L(T_{/e})=L(T)$, is obtained by contraction of the edge *e* in *T* and by keeping the edge labels of all non-contracted edges.

Note, if *e* is an inner edge of a phylogenetic tree *T*, then the tree $T_{/e}$ is again phylogenetic.

#### Definition 20

An edge *e* in $(T,\lambda )$ is *relevantly-labeled in* $(T,\lambda )$ if, for the tree $(T,\lambda ')$ with $\lambda '(f)=\lambda (f)$ for all $f\in E(T){\setminus }\{e\}$ and $\lambda '(e)\ne \lambda (e)$, it holds that $\digamma (T,\lambda )\ne \digamma (T,\lambda ')$.

#### Lemma 14

An outer 0-edge $e=(v,x)$ in $(T,\lambda )$ is *relevantly-labeled in* $(T,\lambda )$ if and only if $zx\notin E(\digamma (T,\lambda ))$ for some $z\in L(T){\setminus } \{x\}$.

#### Proof

Assume that $e=(v,x)$ is a relevantly-labeled outer 0-edge. Hence, for $(T,\lambda ')$ with $\lambda '(f)=\lambda (f)$ for all $f\in E(T){\setminus }\{e\}$ and $\lambda '(e)=1$, it holds that $\digamma (T,\lambda )\ne \digamma (T,\lambda ')$. Since we only changed the label of the outer edge (*v*, *x*), it still holds that $yy'\in E(\digamma (T,\lambda '))$ if and only if $yy'\in E(\digamma (T,\lambda ))$ for all distinct $y,y'\in L(T){\setminus } \{x\}$. Moreover, since $\lambda '(e)=1$ and $e=(v,x)$ is an outer edge, we have $xz\in E(\digamma (T,\lambda '))$ for all $z\in L(T){\setminus } \{x\}$. Thus, $\digamma (T,\lambda )\ne \digamma (T,\lambda ')$ implies that $xz\notin E(\digamma (T,\lambda ))$ for at least one $z\in L(T){\setminus } \{x\}$.

Now, suppose that $zx\notin E(\digamma (T,\lambda ))$ for some $z\in L(T){\setminus } \{x\}$. Clearly, this implies that the outer edges $e=(v,x)$ and $f=(w,z)$ must be 0-edges and changing one of them to a 1-edge would imply that *xz* becomes an edge in the Fitch graph. Hence, *e* is relevantly-labeled in $(T,\lambda )$. $\square$

#### Lemma 15

For every tree $(T,\lambda )$ and every inner 0-edge *e* of *T*, it holds $\digamma (T,\lambda )=\digamma (T_{/e},\lambda _{/e})$.

#### Proof

Suppose that $(T,\lambda )$ contains an inner 0-edge $e=(u,v)$. The contraction of this edge does not change the number of 1-edges along the paths connecting any two leaves. It affects the least common ancestor of *x* and *y*, if ${{\,\mathrm{lca}\,}}_T(x, y) = u$ or ${{\,\mathrm{lca}\,}}_T (x, y) = v$. In either case, however, the number of 1-edges between ${{\,\mathrm{lca}\,}}_T (x, y)$ and the leaves *x* and *y* remains unchanged. Hence, we have $\digamma (T,\lambda ) = \digamma (T_{/e},\lambda _{/e})$. $\square$

#### Lemma 16

If $(T,\lambda )$ is a Fitch-least-resolved tree w.r.t. $\digamma (T,\lambda )$, then it does neither contain inner 0-edges nor inner 1-edges that are not relevantly-labeled.

#### Proof

Suppose first, by contraposition, that $(T,\lambda )$ contains an inner 0-edge $e=(u,v)$. By Lemma 15, $\digamma (T,\lambda ) = \digamma (T_{/e},\lambda _{/e})$, and thus, $(T,\lambda )$ is not Fitch-least-resolved.

Assume now, by contraposition, that $(T,\lambda )$ contains an inner 1-edge *e* that is not relevantly-labeled. Hence, we can put $\lambda '(e)=0$ and $\lambda (f)=\lambda (f')$ for all $f\in E(T){\setminus } \{e\}$ and obtain $\digamma (T,\lambda ) = \digamma (T,\lambda ')$. Since $(T,\lambda ')$ contains an inner 0-edge, it cannot be Fitch-least-resolved. Therefore and by definition, $(T,\lambda )$ cannot be Fitch-least-resolved as well. $\square$

The converse of Lemma 16 is, however, not always satisfied. To see this, consider the Fitch graph $G \simeq K_3$ with vertices *x*, *y* and *z*. Now, consider the tree $(T,\lambda )$ where *T* is the triple *xy*|*z*, the two outer edges incident to *y* and *z* are 0-edges while the remaining two edges in *T* are 1-edges. It is easy to verify that $G=\digamma (T,\lambda )$. In particular, the inner edge *e* is relevantly-labeled, since if $\lambda '(e) = 0$ we would have $yz\notin E(\digamma (T,\lambda '))$. However, $(T,\lambda )$ is not Fitch-least-resolved w.r.t. *G*, since the star tree $T'$ on the three leaves *x*, *y*, *z* is displayed by *T*, and the labeling $\lambda '$ with $\lambda '(e)=1$ for all $e\in E(T')$ provides a tree $(T',\lambda ')$ with $G=\digamma (T',\lambda ')$.

#### Lemma 17

A tree $(T,\lambda )$ is a Fitch-least-resolved tree w.r.t. $\digamma (T,\lambda )$ if and only if $\digamma (T,\lambda ) \ne \digamma (T_{/e},\lambda ')$ holds for all labelings $\lambda '$ of $T_{/e}$ and all inner edges *e* in *T*.

#### Proof

Let $(T,\lambda )$ be an edge-labeled tree. Suppose first that $(T,\lambda )$ is Fitch-least-resolved w.r.t. $\digamma (T,\lambda )$. For every inner edge *e* in *T*, the tree $T_{/e}\ne T$ is displayed by *T*. By definition of Fitch-least-resolved trees, we have $\digamma (T,\lambda )\ne \digamma (T_{/e},\lambda ')$ for every labeling $\lambda '$ of $T_{/e}$.

For the converse, assume, for contraposition, that $(T,\lambda )$ is not Fitch-least-resolved w.r.t. $\digamma (T,\lambda )$. Hence, there is a tree $(T',\lambda ')$ such that $T'\ne T$ is displayed by *T* and $\digamma (T,\lambda ) = \digamma (T',\lambda ')$. Clearly, *T* and $T'$ must have the same leaf set. Therefore and since $T'<T$, the tree $T'$ can be obtained from *T* by a sequence of contractions of inner edges $e_1,\dots ,e_{\ell }$ (in this order) where $\ell \ge 1$. If $\ell =1$, then we have $T'=T_{/e_1}$ and, by assumption, $\digamma (T,\lambda ) = \digamma (T_{/e_1},\lambda ')$. Thus, we are done. Now assume $\ell \ge 2$. We consider the tree $(T_{/e_1},\lambda '')$ where $\lambda ''(f)=\lambda '(f)$ if $f \in E(T')$ and $\lambda ''(f)=0$ otherwise. Hence, $(T',\lambda ')$ can be obtained from $(T_{/e_1},\lambda '')$ by stepwise contraction of the 0-edges $e_2,\dots ,e_{\ell }$, and by keeping the labeling of $\lambda ''$ for the remaining edges in each step. Hence, we can repeatedly apply Lemma 15 to conclude that $\digamma (T_{/e_1},\lambda '')=\digamma (T',\lambda ')$. Together with $\digamma (T,\lambda ) = \digamma (T',\lambda ')$, we obtain $\digamma (T,\lambda ) = \digamma (T_{/e_1},\lambda '')$, which completes the proof. $\square$

As a consequence of Lemma 17, it suffices to show that $\digamma (T,\lambda ) = \digamma (T_{/e},\lambda ')$ for some inner edge $e\in E(T)$ and some labeling $\lambda '$ for $T_{/e}$ to show that $(T,\lambda )$ is not Fitch-least-resolved tree w.r.t. $\digamma (T,\lambda )$. The next result characterizes Fitch-least-resolved trees and is very similar to the results for “directed” Fitch graphs of 0/1-edge-labeled trees (cf. Lemma 11(1,3) in Geiß et al. 2018). However, we note that we defined Fitch-least-resolved in terms of all possible labelings $\lambda '$ for trees $T'$ displayed by *T*, whereas Geiß et al. (2018) call $(T,\lambda )$ least-resolved whenever $(T_{/e},\lambda _{/e})$ results in a (directed) Fitch graph that differs from the one provided by $(T,\lambda )$ for every $e\in E(T)$.

#### Theorem 7

Let *G* be a Fitch graph, and $(T,\lambda )$ be a tree such that $G=\digamma (T,\lambda )$. If all independent sets of *G* are of size one (except possibly for one independent set), then $(T,\lambda )$ is Fitch-least-resolved for *G* if and only if it is a star tree.

If *G* has at least two independent sets of size at least two, then $(T,\lambda )$ is Fitch-least-resolved for *G* if and only if

- every inner edge of $(T,\lambda )$ is a 1-edge,
- for every inner vertex $v\in V^0(T)$ there are (at least) two relevantly-labeled outer 0-edges (*v*, *x*), (*v*, *y*) in $(T,\lambda )$

In particular, if distinct $x, y\in L(T)$ are in the same independent set of *G*, then they have the same parent in *T* and $({{\,\mathrm{par}\,}}(x), x)$, $({{\,\mathrm{par}\,}}(x), y)$ are relevantly-labeled outer 0-edges.

#### Proof

Suppose that every independent set of *G* is of size one (except possibly for one). Let $(T,\lambda )$ be the star tree where $\lambda ((\rho _T,v)) =1$ if and only if *v* is the single element in an independent set of size one. It is now a simple exercise to verify that $G=\digamma (T,\lambda )$. Since $(T,\lambda )$ is a star tree, it is clearly Fitch-least-resolved. The converse follows immediately from this construction together with fact that the star tree is displayed by all trees with leaf set *V*(*G*). In the following we assume that *G* contains at least two independent sets of size at least two.

First suppose that $(T,\lambda )$ is Fitch-least resolved w.r.t. $\digamma (T,\lambda )$. By Lemma 16, Condition (a) is satisfied. We continue with showing that Condition (b) is satisfied. In particular, we show first that every inner vertex $v\in V^0(T)$ is incident to at least one relevantly-labeled outer 0-edge. To this end, assume, for contradiction, that $(T,\lambda )$ contains an inner vertex $v\in V^0(T)$ for which this property is not satisfied.

That is, *v* is either (i) incident to 1-edges only (incl. $\lambda (({{\,\mathrm{par}\,}}_T(v),v))=1$ in case $v\ne \rho _T$ by Condition (a)) or (ii) there is an outer 0-edge (*v*, *x*) that is not relevantly-labeled. In Case (i), we put $\lambda '=\lambda$. In Case (ii), we obtain a new labeling $\lambda '$ by changing the label of every outer 0-edge (*v*, *x*) with $x\in {{\,\mathrm{child}\,}}_T(v) \cap L(T)$ to “1” while keeping the labels of all other edges. This does not affect the Fitch graph, since every such 0-edge is not relevantly-labeled, and thus, $zx\in E(\digamma (T,\lambda ))$ for all $z\in L(T){\setminus } \{x\}$ by Lemma 14. Hence, for both Cases (i) and (ii), for the labeling $\lambda '$ *all* outer edges (*v*, *x*) with $x\in {{\,\mathrm{child}\,}}(v)\cap L(T)$ are labeled as 1-edges, *v* is incident to 1-edges only (by Condition (a)) and $\digamma (T,\lambda ) = \digamma (T,\lambda ')$. We thus have $xy\in E(\digamma (T,\lambda ')) =E(\digamma (T,\lambda ))$ for all $x\in L(T(v))$ and $y\in L(T){\setminus } L(T(v))$. Now, if $v\ne \rho _T$ let $e=(u:={{\,\mathrm{par}\,}}_T(v),v)$. Otherwise, if $v=\rho _T$ then let $e=(v,u)$ for some inner vertex $u\in {{\,\mathrm{child}\,}}_T(v)$. Note, such an inner edge $(\rho _T,u)$ exists since *G* contains at least two independent sets of size at least two and *T* is not a star tree as shown above. Now consider the tree $(T_{/e},\lambda '_{/e})$, and denote by *w* the vertex obtained by contraction of the inner edge *e*. By construction, every path in $T_{/e}$ connecting any $x\in L(T(v))$ and $y\in L(T){\setminus } L(T(v))$ must contain some 1-edge $(w,w')$ with $w'\in {{\,\mathrm{child}\,}}_{T_{/e}}(w)={{\,\mathrm{child}\,}}_{T}(v)$ implying $xy\in E(\digamma (T_{/e},\lambda '_{/e}))$. Moreover, the edge contraction does not affect whether or not the path between any vertices within *L*(*T*(*v*)) or within $L(T){\setminus } L(T(v))$ contains a 1-edge. Hence, $\digamma (T,\lambda ) = \digamma (T,\lambda ') = \digamma (T_{/e},\lambda '_{/e})$, and $(T,\lambda )$ is not Fitch-least-resolved; a contradiction. In summary, every inner vertex *v* must be incident to at least one relevantly-labeled outer 0-edge (*v*, *x*). By Lemma 14, (*v*, *x*) is a relevantly-labeled outer 0-edge if and only if there is a vertex $z\in L(T){\setminus } \{x\}$ such that $zx\notin E(\digamma (T,\lambda ))$. By Condition (a), all inner edges in $(T,\lambda )$ are 1-edges, and thus, there is only one place where the leaf *z* can be located in *T*, namely as a leaf adjacent to *v*. In particular, the outer edge (*v*, *z*) is a relevantly-labeled 0-edge, since $zx\notin E(\digamma (T,\lambda ))$. Therefore, Condition (b) is satisfied for every inner vertex *v* of *T*.

The latter arguments also show that all distinct vertices $x,y\in L(T)$ that are contained in the same independent set must have the same parent. Clearly, $({{\,\mathrm{par}\,}}(x), x)$, $({{\,\mathrm{par}\,}}(x), y)$ must be outer 0-edges, since otherwise $xy\in E(\digamma (T,\lambda ))$. Hence, the final statement of the theorem is satisfied.

Now let $(T,\lambda )$ be such that Conditions (a) and (b) are satisfied. First observe that none of the outer edges can be contracted without changing *L*(*T*). Now let $e = (u,v)$ be an inner edge. By Condition (a), *e* is a 1-edge. Moreover, by Condition (b), vertex *u* and *v* are both incident to at least two relevantly-labeled outer 0-edges. Hence, there are outer 0-edges $(u,x),(u,x'),(v,y),(v,y')$ with pairwise distinct leaves $x,x',y,y'$ in *T*. Since (*u*, *v*) is a 1-edge, we have $xy,xy',x'y,x'y' \in E(\digamma (T,\lambda ))$. Moreover, we have $xx',yy'\notin E(\digamma (T,\lambda ))$. Now consider the tree $(T_{/e}, \lambda ')$ with an arbitrary labeling $\lambda '$ and denote by *w* the vertex obtained by contraction of the inner edge (*u*, *v*). In this tree, $x,x',y,y'$ all have the same parent *w*. If $\lambda '((w,x))=1$ *or* $\lambda '((w,y))=1$, we have $xx'\in \digamma (T_{/e}, \lambda ')$ or $yy'\in E(\digamma (T_{/e}, \lambda '))$, respectively. If $\lambda '((w,x))=0$ *and* $\lambda '((w,y))=0$, we have $xy\notin E(\digamma (T_{/e}, \lambda '))$. Hence, it holds $\digamma (T_{/e}, \lambda ')\ne \digamma (T, \lambda )$ in both cases. Since the inner edge *e* and $\lambda '$ were chosen arbitrarily, we can apply Lemma 17 to conclude that $(T,\lambda )$ is Fitch-least-resolved. $\square$

As a consequence of Theorem 7, Fitch-least-resolved trees can be constructed in polynomial time. To be more precise, if a Fitch graph *G* contains only independent sets of size one (except possibly for one), we can construct a star tree *T* with edge labeling $\lambda$ as specified in the proof of Theorem 7 to obtain the 0/1-edge labeled tree $(T,\lambda )$ that is Fitch-least-resolved w.r.t. *G*. This construction can be done in *O*(|*V*(*G*)|) time.

Now, assume that *G* has at least two independent sets of size at least two. Let ${\mathscr {I}}$ be the set of independent sets of *G* and $I_1,\dots ,I_k\in {\mathscr {I}}$, $k\ge 2$ be all independent sets of size at least two. We now construct a tree $(T,\lambda )$ with root $\rho _T$ as follows: First we add *k* vertices $v_1 = \rho _T$ and $v_2,\dots ,v_{k}$, and add inner edges $e_i=(v_i,v_{i+1})$ with label $\lambda (e_i)=1$, $1\le i\le k-1$. Each vertex $v_i$ gets as children the leaves in $I_i$, $1\le i\le k$ and all these additional outer edges obtain label “0”. Finally, all elements in the remaining independent sets ${\mathscr {I}}{\setminus } \{I_1,\dots ,I_k\}$ are of size one and are connected as leaves via outer 1-edges to the root $v_1=\rho _T$. It is an easy exercise to verify that *T* is a phylogenetic tree and that $\digamma (T,\lambda )=G$. In particular, Theorem 7 implies that $(T,\lambda )$ is Fitch-least-resolved w.r.t. *G*. This construction can be done in *O*(|*V*(*G*)|) time. We summarize this discussion as

#### Proposition 7

For a given Fitch graph *G*, a Fitch-least-resolved tree can be constructed in *O*(|*V*(*G*)|) time.

Fitch-least-resolved trees, however, are only of very limited use for the construction of relaxed scenarios ${\mathcal {S}}=(T,S,\sigma ,\mu ,\tau _{T},\tau _{S})$ from an underlying Fitch graph. First note that we would need to consider *planted versions* of Fitch-least-resolved trees, i.e., Fitch-least-resolved trees to which a planted root is added, since otherwise, such trees cannot be part of an explaining scenario, which is defined in terms of planted trees. Even though $(G,\sigma )$ is an rs-Fitch graph, Example 3 shows that it is possible that there is no relaxed scenario ${\mathcal {S}}=(T,S,\sigma ,\mu ,\tau _{T},\tau _{S})$ with HGT-labeling $\lambda _{{\mathcal {S}}}$ such that $(T,\lambda ) = (T,\lambda _{{\mathcal {S}}})$ for the planted version $(T,\lambda )$ of *any* of its Fitch-least-resolved trees.

#### Example 3

Consider the rs-Fitch graph $(G,\sigma )$ with $V(G)=\{a,b,b',c\}$, $E(G)=\{ab',ac,bb',bc\}$ and surjective coloring $\sigma$ such that $\sigma (a)=A$, $\sigma (b)=\sigma (b')=B$, $\sigma (c)=C$ and *A*, *B*, *C* are pairwise distinct. The rs-Fitch graph $(G,\sigma )$, a Fitch tree $(T,\lambda )$ and relaxed scenario ${\mathcal {S}}$ with $(T,\lambda ) = (T,\lambda _{{\mathcal {S}}})$ as well as the planted versions $(T_1,\lambda _1)$ and $(T_2,\lambda _2)$ of its two Fitch-least-resolved trees are shown in Fig. 20.

Fitch-least-resolved trees for $(G,\sigma )$ must contain an inner 1-edge, since *G* has two independent sets of size two and by Theorem 7. Thus, it is easy to verify that there are no other Fitch-least-resolved trees for $(G,\sigma )$.

By Lemma 13, we obtain ${{\,\mathrm{lca}\,}}_S(A,B) \preceq _S \mu ({{\,\mathrm{lca}\,}}_{T_i}(a,b))$ and ${{\,\mathrm{lca}\,}}_S(B,C) \preceq _S \mu ({{\,\mathrm{lca}\,}}_{T_i}(b',c))$, $i\in \{1,2\}$, for both (planted versions of the) Fitch-least-resolved trees. However, for all of the possible species trees on three leaves *A*, *B*, *C*, this implies that the images $\mu ({{\,\mathrm{lca}\,}}_{T_i}(a,b))$ and $\mu ({{\,\mathrm{lca}\,}}_{T_i}(b',c))$ are the single inner edge or the edge $(0_T,\rho _T)$ in *S*. Therefore, $\mu ({{\,\mathrm{lca}\,}}_{T_i}(a,b))$ and $\mu ({{\,\mathrm{lca}\,}}_{T_i}(b',c))$ are always comparable in *S*. Hence, for all possible relaxed scenarios ${\mathcal {S}}$, we have $\lambda _{{\mathcal {S}}}(e)=0$ for the single inner edge *e*, whereas $\lambda _i(e)=1$ in $T_i$, $i\in \{1,2\}$. This implies that there is no relaxed scenario ${\mathcal {S}}$ with $(T_i,\lambda _i) = (T_i,\lambda _{{\mathcal {S}}})$, $i\in \{1,2\}$.

## Editing problems

### Editing colored graphs to LDT graphs and Fitch graphs

We consider the following two edge modification problems for completion, deletion, and editing.

#### Problem 7

(LDT-Graph-Modification (LDT-M))

*Input:*: A colored graph $(G =(V,E),\sigma )$ and an integer *k*.
*Question:*: Is there a subset $F\subseteq E$ such that $|F|\le k$ and $(G'=(V,E\star F),\sigma )$ is an LDT graph where $\star \in \{{\setminus }, \cup , \varDelta \}$?

#### Problem 8

(rs-Fitch Graph-Completion/Editing (rsF-D/E))

*Input:*: A colored graph $(G =(V,E),\sigma )$ and an integer *k*.
*Question:*: Is there a subset $F\subseteq E$ such that $|F|\le k$ and $(G'=(V,E\star F),\sigma )$ is an rs-Fitch graph where $\star \in \{{\setminus }, \cup , \varDelta \}$?

NP-completeness of LDT-M be shown by reduction from

#### Problem 9

(Maximum Rooted Triple Compatibility (MaxRTC))

*Input:*: A set of (rooted) triples ${\mathcal {R}}$ and an integer *k*.
*Question:*: Is there a compatible subset ${\mathcal {R}}^*\subseteq {\mathcal {R}}$ such that $|{\mathcal {R}}^*|\ge |{\mathcal {R}}|-k$?

#### Theorem 8

(Jansson 2001, Thm. 1) MaxRTC is NP-complete.

#### Theorem 9

LDT-M is NP-complete.

#### Proof

Since LDT graphs can be recognized in polynomial time (cf. Corollary 2), a given solution can be verified in polynomial time. Thus, LDT-M is contained in NP.

We now show NP-hardness by reduction from MaxRTC. Let $({\mathcal {R}},k)$ be an instance of this problem, i.e., ${\mathcal {R}}$ is a set of triples and *k* is a non-negative integer. We construct a colored graph $(G_{\mathcal {R}}=(L,E),\sigma )$ as follows: For each triple $r_i = xy|z\in {\mathcal {R}}$, we add three vertices $x_i,y_i,z_i$, two edges $x_iz_i$ and $y_iz_i$, and put $\sigma (x_i) = x$, $\sigma (y_i) = y$ and $\sigma (z_i) = z$. Hence, $(G_{\mathcal {R}},\sigma )$ is properly colored and the disjoint union of paths on three vertices $P_3$. In particular, therefore, $(G_{\mathcal {R}},\sigma )$ does not contain an induced $P_4$, and is therefore a properly colored cograph (cf. Proposition 2). By definition and construction, we have ${\mathcal {R}} = {\mathfrak {S}}(G_{\mathcal {R}},\sigma )$.

First assume that MaxRTC with input $({\mathcal {R}}, k)$ has a yes-answer. In this case let ${\mathcal {R}}^*\subseteq {\mathcal {R}}$ be a compatible subset such that $|{\mathcal {R}}^*| \ge |{\mathcal {R}}| - k$. For each of the triples $r_i= xy|z\in {\mathcal {R}}{\setminus }{\mathcal {R}}^*$, we add the edge $x_iy_i$ to $G_{\mathcal {R}}$ or remove the edge $x_iz_i$ from $G_{\mathcal {R}}$ for LDT-E/C and LDT-D, respectively, to obtain the graph $G^*$. In both cases, we eliminate the corresponding triple *xy*|*z* from ${\mathfrak {S}}(G^*,\sigma )$. By construction, therefore, we observe that ${\mathfrak {S}}(G^*,\sigma ) = {\mathcal {R}}^*$ is compatible. Moreover, since we have never added edges between distinct $P_3$s, all connected components of $G^*$ are of size at most three. Therefore, $G^*$ does not contain an induced $P_4$, and thus remains a cograph. By Theorem 3, the latter arguments imply that $(G^*,\sigma )$ is an LDT graph. Since $(G^*,\sigma )$ was obtained from $(G_{\mathcal {R}},\sigma )$ by using $|{\mathcal {R}}{\setminus }{\mathcal {R}}^*| \le k$ edge modifications, we conclude that LDT-M with input $(G_{\mathcal {R}},\sigma , k)$ has a yes-answer.

For the converse, suppose that LDT-M with input $(G_{\mathcal {R}},\sigma , k)$ has a yes-answer with a solution $(G^* = (L,E\star F),\sigma )$, i.e., $(G^*,\sigma )$ is an LDT graph and $|F|\le k$. By Theorem 3, ${\mathfrak {S}}(G^*,\sigma )$ is compatible. Let ${\mathcal {R}}^*$ be the subset of ${\mathcal {R}} = {\mathfrak {S}}(G_{\mathcal {R}},\sigma )$ containing all triples of ${\mathcal {R}}$ for which the corresponding induced $P_3$ in $G_{\mathcal {R}}$ remains unmodified and thus, is still an induced $P_3$ in $G^*$. By construction, we have ${\mathcal {R}}^*\subseteq {\mathfrak {S}}(G^*,\sigma )$. Hence, ${\mathcal {R}}^*$ is compatible. Moreover, since $|F|\le k$, at most *k* of the vertex-disjoint $P_3$s have been modified. Therefore, we conclude that $|{\mathcal {R}}^*|\ge |{\mathcal {R}}|-k$.

In summary, LDT-M is NP-hard. $\square$

#### Theorem 10

rsF-C and rsF-E are NP-complete.

#### Proof

Since rs-Fitch graphs can be recognized in polynomial time, a given solution can be verified as being a yes- or no-answer in polynomial time. Thus, rsF-C/E$\in NP$.

Consider an arbitrary graph *G* and an integer *k*. We construct an instance $(G,\sigma ,k)$ of rsF-C/E by coloring all vertices distinctly. Then condition (ii) in Theorem 6 is always satisfied. To see this, we note that for $k>1$ there are no edges between colors in the auxiliary graph ${\mathcal {A}}_{\digamma }(\sigma ,{\mathscr {I}})$ such that their corresponding unique vertices are in distinct independent sets $I, I'\in {\mathscr {I}}$. The problem therefore reduces to completion/editing of $(G,\sigma )$ to a complete multipartite graph, which is equivalent to a complementary deletion/editing of the complement of (*G*, *k*) to a disjoint union of cliques, i.e., a cluster graph. Both Cluster Deletion and Cluster Editing are NP-hard (Shamir et al. 2004). $\square$

Although Cluster Completion is polynomial (it is solved by computing the transitive closure), rsF-D remains open: Consider a colored complete multipartite graph $(G,\sigma )$ that is not an rs-Fitch graph. Then solving Cluster Completion on the complement returns $(G,\sigma )$, which by construction is not a solution to rsF-D.

### Editing LDT graphs to Fitch graphs

#### Lemma 18

There is a linear-time algorithm to solve Problem 3 for every cograph *G*.

#### Proof

Instead of inserting in the cograph *G* the minimum number of edges necessary to reach a complete multipartite graph, we consider the equivalent problem of *deleting* a minimal set *Q* of edges from its complement ${\overline{G}}$, which is also a cograph, to obtain the complement of a complete multipartite graph, i.e., the disjoint union of complete graphs. This problem is known as the Cluster Deletion problem (Shamir et al. 2004), which is known to have an polynomial-time solution for cographs (Gao et al. 2013): A greedy maximum clique partition of *G* is obtained by recursively removing a maximum clique *K* from *G*, see also Dessmark et al. (2007). For cographs, the greedy maximum clique partitions are the solutions of the Cluster Deletion problem (Gao et al. 2013, Thm. 1). The Maximum Clique problem on cographs can be solved in linear time using the co-tree of *G* (Corneil et al. 1981a), which can also be obtained in linear time (Corneil et al. 1981a). $\square$

An efficient algorithm to solve the Cluster Deletion problem for cographs can be devised by making use of the recursive construction of a cograph along its discriminating cotree (*T*, *t*). For all $u\in V(T)$, we have

Denote by ${\mathcal {P}}(u)$ the optimal clique partition of the cograph implied by the subtree *T*(*u*) of the discriminating cotree (*T*, *t*). We think of ${\mathcal {P}}(u) := [Q_1(u),Q_2(u),\dots ]$ as an ordered list, such that $|Q_i(u)|\ge |Q_j(u)|$ if $i<j$. It will be convenient to assume that the list contains an arbitrary number of empty sets acting as an identity element for the join and disjoint union operation. With this convention, the optimal clique partitions ${\mathcal {P}}(u)$ satisfy the recursion:

$$\begin{aligned} {\mathcal {P}}(u) = {\left\{ \begin{array}{ll} \displaystyle \bigcup _{v\in {{\,\mathrm{child}\,}}(u)} {\mathcal {P}}(v) &{} \text { if } t(u)=0 \\ \displaystyle \left[ \bigcup _{v\in {{\,\mathrm{child}\,}}(u)} Q_i(v) \quad \Big | \; i=1,2,\dots \right] &{} \text { if } t(u)=1 \\ \displaystyle [\{u\},\emptyset ,\dots ] &{} \text { if } u \text { is a leaf } \end{array}\right. } \end{aligned}$$

In the first case, where $t(u)=0$, we assume that the union operation to obtain ${\mathcal {P}}(u) = [Q_1(u),Q_2(u),\dots ]$ maintains the property $|Q_i(u)|\ge |Q_j(u)|$ if $i<j$. In an implementation, this can e.g. be achieved using *k*-way merging where $k=|{{\,\mathrm{child}\,}}(u)|$.

To see that the recursion is correct, it suffices to recall that the greedy clique partition is optimal for cographs as input (Gao et al. 2013) and to observe the following simple properties of cliques in cographs (Corneil et al. 1981a): (i) a largest clique in a disjoint union of graphs is also a largest clique in any of its components. The optimal clique partition of a disjoint union of graphs is, therefore, the union of the optimal clique partitions of the constituent connected components. (ii) For a join of two or more graphs $G_i$, each maximum size clique *Q* is the join of a maximum size clique of each constituent. The next largest clique disjoint from  is, thus, the join of a largest cliques disjoint from $Q_i$ in each constituent graph $G_i$. Thus a greedy clique partition of *G* is obtained by size ordering the clique partitions of $G_i$ and joining the *k*-largest cliques from each.

The recursive construction of ${\mathcal {P}}(\rho _T)$ operates directly on the discriminating cotree (*T*, *t*) of the cograph *G*. For each node *u*, the effort is proportional to $|L(T(u))| \log (\deg (u))$ for the $\deg (u)$-wise merge sort step if $t(u)=0$ and proportional to |*L*(*T*(*u*))| for the merging of the *k*-th largest clusters for $t(u)=1$. Using $\sum _u \deg (u)|L(T(u))|\le |L(T)|\sum _u \deg (u)\le |L(T)|2|E(T)|$ together with $|E(T)|=|V(T)|-1$ and $|V(T)|\le 2 |L(T)|-1$ (cf. Hellmuth et al. 2015, Lemma 1), we obtain $\sum _u \deg (u)|L(T(u))| \in {\mathscr {O}}(|L(T)|^2) = {\mathscr {O}}(|V(G)|^2)$, that is, a quadratic upper bound on the running time.
